# 1-(*N*-Acylamino)alkyltriarylphosphonium Salts with Weakened C_α_-P^+^ Bond Strength—Synthetic Applications

**DOI:** 10.3390/molecules23102453

**Published:** 2018-09-25

**Authors:** Jakub Adamek, Anna Węgrzyk, Justyna Kończewicz, Krzysztof Walczak, Karol Erfurt

**Affiliations:** 1 Department of Organic Chemistry, Bioorganic Chemistry and Biotechnology, Silesian University of Technology, B. Krzywoustego 4, 44-100 Gliwice, Poland; anna.wegrzyk@polsl.pl (A.W.); j.konczewicz@gmail.com (J.K.); krzysztof.walczak@polsl.pl (K.W.); 2 Biotechnology Center of Silesian University of Technology, B. Krzywoustego 8, 44-100 Gliwice, Poland; 3 Department of Chemical Organic Technology and Petrochemistry, Silesian University of Technology, B. Krzywoustego 4, 44-100 Gliwice, Poland; karol.erfurt@polsl.pl

**Keywords:** organophosphorus chemistry, phosphonium salts, α-amidoalkylating agents, *N*-acyliminium cation, *N*-acylimine

## Abstract

The α-amidoalkylating properties of 1-(*N*-acylamino)alkyltriarylphosphonium salts with weakened C_α_-P^+^ bond strength are discussed and examined. It is demonstrated that such type of phosphonium salts reacts smoothly with a diverse array of carbon- and heteroatom-based nucleophiles, including 1-morpholinocyclohexene, 1,3-dicarbonyl compounds, benzotriazole sodium salt, *p*-toluenesulfinate sodium salt, benzylamine, triarylphosphines, and other P-nucleophiles. Reactions are conducted at room temperature, in a short time (5–15 min) and mostly without catalysts. Simple work-up procedures result in good or very good yields of products. The structures of known compounds were established by spectroscopic methods and all new compounds have been fully characterized using ^1^H-, ^13^C-, ^31^P-NMR, IR spectroscopy, and high-resolution mass spectrometry. Mechanistic aspects of described transformations are also performed and discussed. It was demonstrated that unique properties make 1-(*N*-acylamino)alkyl-triarylphosphonium salts with weakened C_α_-P^+^ bond strength interesting building blocks with great potential, especially in α-amidoalkylation reactions.

## 1. Introduction

Specific structural features of 1-(*N*-acylamino)alkylphosphonium salts make them very interesting reagents. The presence of a positively charged nucleofugal phosphonium moiety in the close surroundings of the *N*-acyl group determines its unique chemical properties such as high reactivity in α-amidoalkylations [1,2,3,4,5,6,7,8,9,10]. This type of reactions has enjoyed unflagging interest for years as a synthetic method with great potential, especially valuable for C-C and C-heteroatom bond formation [1,11,12,13,14,15,16,17,18,19,20,21,22,23,24,25,26,27,28,29,30,31,32,33,34,35,36,37].

**Scheme 1:**
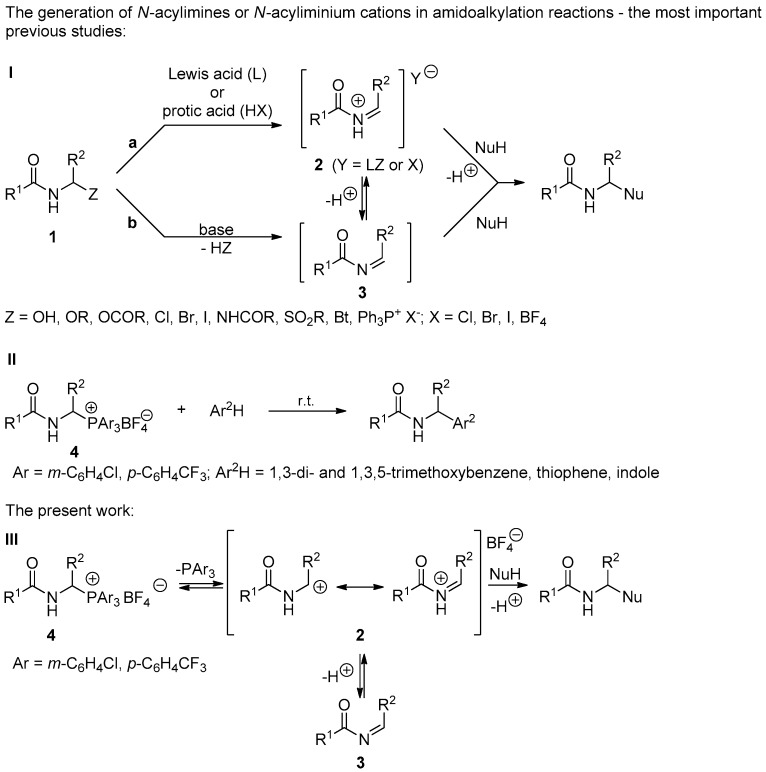
α-Amidoalkylation reactions—various synthetic routes.

In general, the reactivity of α-amidoalkylating agents **1** depends on the efficiency of the generation of *N*-acyliminium cation **2** or *N*-acylimine **3** from its precursor and the equilibrium constant of this reaction. Of course, the reactivity of *N*-acyliminium cation **2** or *N*-acylimine **3** toward a nucleophile is also significant [1,2,3]. To produce the proper α-amidoalkylating agents, for instance *N*-acyliminium cation **2** or *N*-acylimine **3** from the most popular precursors such as α-amido sulfones (Z = SO_2_Ar), *N*-(1-benzotriazolil)alkylamides (Z = Bt), and *N*-(1-alkoxyalkyl)amides (Z = OR), it is necessary to use catalysts, mainly Lewis acids (route **a**, Scheme 1 (I)) [18,19,20,21,22,23,24,25,29,30,31,32,33,34,35,36,37]. In contrast, 1-(*N*-acylamino)alkyltriphenylphosphonium salts **1** (Z = Ph_3_P^+^ X^−^) do not require the use of acidic catalysts because of the permanent positive charge on the phosphonium group. However, the relatively high stability of C_α_-P^+^ bond forces the use of a base catalyst (e.g., Hünig’s base, DBU, TBD; route **b**, Scheme 1 (I)) and sometimes microwave radiation [1,4,5,6,7,8,9,10].

Recently, we have proven that the use of 1-(*N*-acylamino)alkyltriarylphosphonium salts **4** derived from the EWG-substituted triarylphosphines facilitates the cleavage of C_α_-P^+^ bond and thereby the generation of *N*-acyliminium cation (Scheme 1 (II)) [2,3]. This phenomenon significantly increases the reactivity of 1-(*N*-acylamino)alkyltriarylphosphonium salts with weakened C_α_-P^+^ bond strength and allows us to conduct α-amidoalkylation without the need for any catalyst. In this work, we demonstrate that the abovementioned phosphonium salts **4** react smoothly with various nucleophiles in a short time under mild conditions and create new carbon-carbon or carbon-heteroatom bonds with good or very good yields (Scheme 1 (III)).

## 2. Results and Discussion

**Scheme 2:**
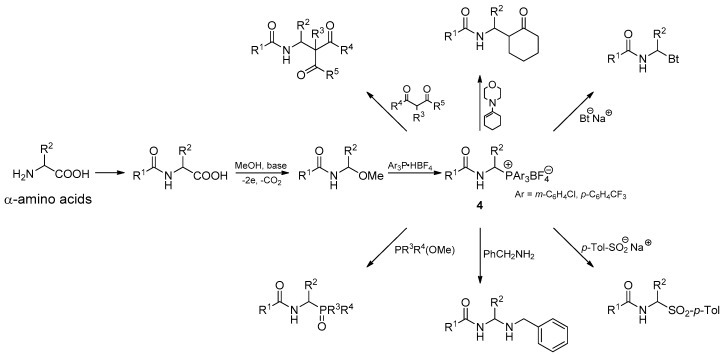
1-(*N*-Acylamino)alkyltriarylphosphonium salts with weakened C_α_-P^+^ bond strength **4**—preparation and synthetic application.

In this section, we focus our studies on the reactivity of phosphonium salts **4** with weakened C_α_-P^+^ bond strength in α-amidoalkylation of various types of carbon- and heteronucleophiles, as shown in Scheme 2. 1-Morpholinocyclohexene and 1,3-dicarbonyl compounds, such as dimethyl malonate, diethyl malonate, and ethyl acetoacetate, are used as carbon nucleophiles. In the case of heteronucleophiles, the reaction toward benzotriazole sodium salt, *p*-toluenesulfinate sodium salt, benzylamine, triarylphosphines, trimethyl phosphite, dimethyl phenylphosphonite, and methyl diphenylphosphinite is examined.

The synthesis of 1-(*N*-acylamino)alkyltriarylphosphonium salts **4** from α-amino acids was performed according to our previously described procedure, for which the electrochemical decarboxylative α-methoxylation of *N*-acyl-α-amino acids and substitution of the methoxy group by triarylphosphonium moiety are crucial steps (see Scheme 2) [2,9].

### 2.1. 1-(N-Acylamino)alkyltriarylphosphonium Salts with Weakened C_α_-P^+^ Bond Strength in the Selected Reaction of C-C Bond Formation

One of the crucial issues of organic synthesis is the formation of new C-C bonds. The possibility of an effective extension of the carbon skeleton is important in many fields, especially in medicinal chemistry, agrochemical synthesis, or in the synthesis of natural products.

Several years ago, we had proved that 1-(*N*-acylamino)alkyltriphenylphosphonium salts **4** (Ar = Ph) reacted quite easily with 1,3-dicarbonyl compounds in the presence of a base such as DBU (1,8-diazabicyclo[5.4.0]undec-7-ene), DBN (1,5-diazabicyclo[4.3.0]non-5-ene), or TBD (1,5,7-triaza- bicyclo[4.4.0]dec-5-ene) under microwave irradiation at 60 °C. However, the use of organic bases (DBU, DBN, TBD) complicates the course of α-amidoalkylation reaction due to the formation of amidinium and guanidinium salts [10]. To avoid this, in the current work, we used lithium diisopropylamide (LDA) as a base to produce enolate anions from the corresponding 1,3-dicarbonyl compounds. In our protocol, α-amidoalkylation was conducted under argon, in THF, at room temperature. A THF solution of enolate anions generated using LDA was introduced into the THF solution of the phosphonium salt **4**. The reaction was performed for 15 min, and the use of microwave irradiation was not necessary. In addition, it was found that the most favorable molar ratio of phosphonium salt **4**: 1,3-dicarbonyl compound: LDA was 1:8:1 (compare experiments 1 and 2, Table 1).

Under the abovementioned conditions, phosphonium salts with weakened C_α_-P^+^ bond strength **4** react with 1,3-dicarbonyl compounds, including diethyl and dimethyl malonate and ethyl acetoacetate, to give the corresponding products **6** with good yields, regardless of whether tris(4-trifluoromethylphenyl)- or tris(3-chlorophenyl)phosphonium salts were used as substrates. Only in the case of phosphonium salts with the benzyloxy carbamate protective group, the yields were significantly lower and did not exceed 30% (entry 8, Table 1).

α-Amidoalkylation of carbon nucleophiles was successfully extended to enamines. Based on the recently described protocol [8], we have demonstrated that 1-(*N*-acylamino)alkyltriarylphosphonium salts **4** with weakened C_α_-P^+^ bond strength react with 1-morpholinocyclohexene in Stork-type enamination to give the expected products **8** with good yields. We have proved that this reaction can be conducted in acetonitrile, at room temperature and without using any base catalysts. The optimized reaction time was 60 min, and the molar ratio of phosphonium salt to 1-morpholinocyclohexene was 1:2. We were able to separate the major diastereoisomer from the mixture using column chromatography and crystallization technique. Furthermore, the use of phosphonium salts with the benzyloxy carbamate protective group resulted in the decrease in reaction efficiency, as already observed for reactions with 1,3-dicarbonyl compounds (see Table 2).

### 2.2. 1-(N-Acylamino)alkyltriarylphosphonium Salts with Weakened C_α_-P^+^ Bond Strength in α-Amidoalkylation of Selected Heteronucleophiles

Applications of 1-(*N*-acylamino)alkyltriphenylphosphonium salts **4** (Ar = Ph) in the α-amidoalkylation of heteronucleophiles have been reported many times in the literature [5,6,7,8,10]. Usually in these types of reactions, it was necessary to use base catalysts, elevated temperature, and microwave irradiation. The effect of the use of 1-(*N*-acylamino)alkyltriarylphosphonium salts with weakened C_α_-P^+^ bond strength **4** (R = *m*-C_6_H_4_Cl, *p*-C_6_H_4_CF_3_) on α-amidoalkylation has not been investigated so far. Therefore, we selected several heteronucleophiles such as benzotriazole sodium salt, *p*-toluenesulfinate sodium salt, benzylamine, and triphenylphosphine, and examined their α-amidoalkylation by phosphonium salts with weakened C_α_-P^+^ bond strength. As it was expected, reactions of salts with a modified structure of the phosphonium group occurred much faster and under mild conditions. In none of the described examples was it necessary to use a base catalyst. The reaction time was only 5–15 min. α-Amidoalkylation of benzotriazole sodium salt and sodium *p*-toluenesulfinate (entries 1–3, Table 3) was conducted at room temperature in CHCl_3_. Similar conditions were used for reaction with benzylamine (entries 4 and 5, Table 3). The obtained aminal derivatives **10d** and **10e** exhibited limited stability. Therefore, the use of excess amount of the nucleophile was required to make the alkaline environment safe for aminals.

**Scheme 3:**
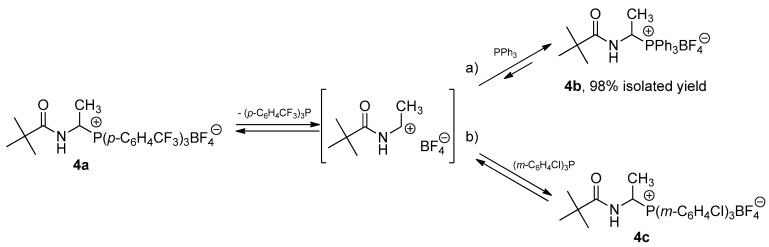
1-(*N*-Acylamino)alkyltriarylphosphonium salts with weakened C_α_-P^+^ bond strength **4**—reactions with triarylphosphines.

1-(*N*-Acylamino)alkyltriarylphosphonium salts **4** react also with triarylphosphines. For example, 1-(*N*-pivaloylamino)ethyltris(4-trifluoromethylphenyl)phosphonium tetrafluoroborate (**4a**) is completely transformed into 1-(*N*-pivaloylamino)ethyltriphenylphosphonium tetrafluoroborate (**4b**) during the reaction with triphenylphosphine. On the other hand, using tris(3-chlorophenyl)- phosphine as the nucleophile, we obtain a reaction mixture in which both 1-(*N*-pivaloylamino)ethyltris(4-trifluoromethylphenyl)phosphonium tetrafluoroborate (**4a**) and 1-(*N*-pivaloylamino)ethyltris(3-chlorophenyl)phosphonium tetrafluoroborate (**4c**) are present in a molar ratio of 1:3. A mixture of the same composition can also be obtained by the reaction of salt **4c** with tris(4-trifluoromethylphenyl)phosphine. These observations may suggest the existence of some equilibrium, as described by the equations in Scheme 3. The equilibrium of the reaction is shifted toward more stable and less reactive phosphonium salts, which is evident for the reaction with triphenylphosphine (equation a, Scheme 3).

**Scheme 4:**
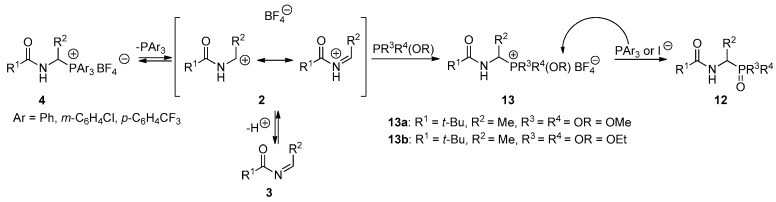
α-Amidoalkylation of P-nucleophiles by 1-(*N*-acylamino)alkyltriarylphosphonium salts **4**—a plausible mechanism.

**Figure 1:**
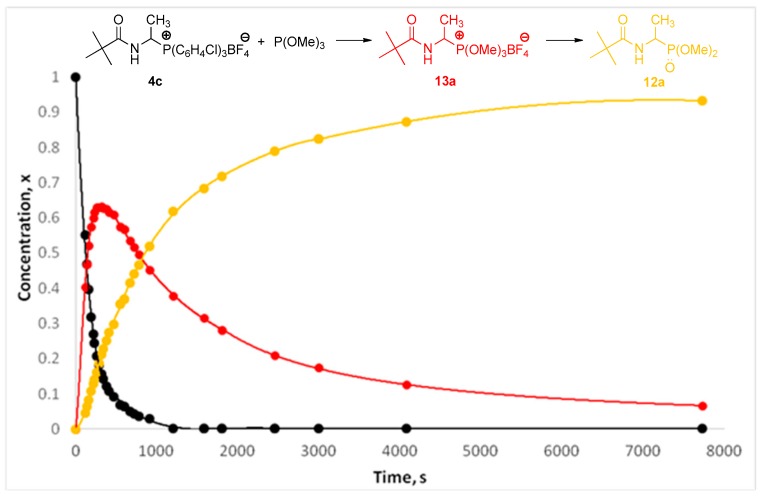
Concentration of the substrate **4c**, intermediate **13a**, and product **12a** as a function of time for the reaction of 1-(*N*-pivaloylamino)ethyltris(3-chlorophenyl)phosphonium tetrafluoroborate (**4c**) with trimethyl phosphite at 26 °C.

The results described above have encouraged us to extend the range of P-nucleophiles by trimethyl phosphite, dimethyl phenylphosphonite, and methyl diphenylphosphinite. It was expected that reactions with these types of phosphorus nucleophiles may occur quickly at room temperature in a non-catalytic environment, as for earlier tested nucleophiles. Surprisingly, the first experiments have shown that reactions are much slower. The analysis of reaction kinetics allowed us to explain these observations. In 2013, we proposed a plausible reaction mechanism wherein the *N*-acyliminium cation or *N*-acylimine, both generated from the 1-(*N*-acylamino)alkyltriphenylphosphonium salt **4** (Ar = Ph), reacts with phosphorus nucleophile to form alkoxyphosphonium salt **13**—the characteristic intermediate of the Michaelis–Arbuzov reaction. The final step of the reaction is the dealkylation of the alkoxyphosphonium salt **13** and may occur directly with triphenylphosphine (see Scheme 4) [7]. Although we have not yet been able to isolate or even observe the formation of postulated intermediate product, we assume that 1-(*N*-acylamino)alkyltriarylphosphonium salts with weakened C_α_-P^+^ bond strength react with phosphorus nucleophiles in an analogous manner. To prove this, we performed the reaction of 1-(*N*-pivaloylamino)ethyltris(3-chlorophenyl)phosphonium tetrafluoroborate (**4c**) with trimethyl phosphite. The reaction progress was monitored using NMR spectroscopy. In the reaction mixture, besides substrates, two products were also detected. Changes in their concentration as a function of time were measured, as shown in Figure 1. The concentration of the first product quickly reaches its maximum and then rapidly decreases, while the concentration of the second one at the beginning of the reaction is low and then increases. The induction period, which is characteristic for the formation of the final product in the consecutive-type reaction, is very clearly visible. Detailed NMR (^1^H- and ^31^P-NMR) and HR-MS analysis confirmed that the first, fast-growing product was 1-(*N*-acylamino)alkyltrimethoxyphosphonium tetrafluoroborate (**13a**) —the postulated intermediate of the Michaelis–Arbuzov reaction. As a result of its demethylation, dimethyl 1-(*N*-pivaloylamino)ethanephosphonate (**12a**) is formed with a much slower reaction. It seems that the second step determines the overall rate of the process.

A plausible mechanism that explains the described kinetic facts is shown in Scheme 4 and is consistent with the mechanism proposed earlier in 2013. The only difference is that the generation of *N*-acyliminium cation from 1-(*N*-acylamino)alkyltriphenylphosphonium salt **4** (Ar = Ph) is more difficult. On the other hand, the highnucleophilicity of triphenylphosphine facilitates dealkylation, which makes the first step crucial for the course of the entire reaction. In the case of 1-(*N*-acylamino)alkyltriarylphosphonium salts with weakened C_α_-P^+^ bond strength **4** (R = *m*-C_6_H_4_Cl, *p*-C_6_H_4_CF_3_), the first step is easier, which facilitates the formation of alkoxyphosphonium salts **13**. However, due to the lower nucleophilicity of phosphines with electron-withdrawing substituents [P(*m*-C_6_H_4_Cl)_3_, P(*p*-C_6_H_4_CF_3_)_3_], the rate of dealkylation drops. To overcome this drawback, we decided to add to the reaction mixture the substoichiometric amounts of methyltriphenylphosphonium iodide as the dealkylating agent (molar ratio of 1:0.25). This protocol allowed us to obtain a series of phosphorus analogs of α-amino acids **12** (*N*-acyl-α-aminoalkanephosphonates **12a–c**, *N*-acyl-α-aminoalkanephosphinate (**12d**) and *N*-acyl-α-aminoalkylphosphine oxide (**12e**)) with good or very good yields. Usually, reactions occur efficiently at room temperature. Only in the reaction with dimethyl phenylphosphonite, it was necessary to raise the temperature to 60 °C. At room temperature, the reaction is very slow even after the addition of methyltriphenylphosphonium iodide (see Table 4).

Finally, we tried to isolate and fully characterize the intermediate of the Michaelis–Arbuzov reaction **13**. To this end, we conducted the reaction of 1-(*N*-pivaloylamino)ethyltris(3-chlorophenyl)phosphonium tetrafluoroborate (**4c**) with trimethyl phosphite. Unfortunately, the expected 1-(*N*-pivaloylamino)ethyltrimethoxyphosphonium tetrafluoroborate (**13a**, Scheme 4) was too reactive and attempts to isolate the analytically pure sample failed. To increase the stability of the intermediate **13**, we used triethyl phosphite as the phosphorus nucleophile. This allowed us to isolate 1-(*N*-pivaloylamino)ethyltriethoxyphosphonium tetrafluoroborate (**13b**) and determine its basic physicochemical properties.

## 3. Experimental Section

### 3.1. General Information

Melting points were determined in capillaries and were uncorrected. IR spectra were measured on an FT-IR spectrophotometer (ATR method). ^1^H- and ^13^C-NMR were recorded at operating frequencies of 400 and 100 MHz, respectively, using TMS as the resonance shift standard. ^31^P-NMR spectra were recorded at an operating frequency of 161.9 MHz without the resonance shift standard, with respect to H_3_PO_4_ as zero ppm. All chemical shifts (δ) are reported in ppm and coupling constants (*J*) in Hz. High-resolution mass spectrometry (HR-MS) analyses were performed on a Xevo G2 Q-TOF mass spectrometer (Waters, Milford, MA, USA) equipped with an ESI source operating in the positive ion mode. The accurate mass and composition of molecular ion adducts were calculated using the MassLynx software incorporated within the instrument.

^1^H, ^13^C, and ^31^P NMR spectra of all new compounds **6**, **8**, **10**, **12**, **13** as well as the summary table in which we compare conditions and yields for reactions of 1-(*N*-acylamino)alkyltriphenylphosphonium salts (former studies) and 1-(*N*-acylamino)alkyltriarylphosphonium salts (the current work) with selected nucleophiles, are placed in the Supplementary data.

### 3.2. Syntheses

#### 3.2.1. Substrate Synthesis

*Electrochemical decarboxylative α-methoxylation of N-acyl-α-amino acids [2,9]*

The electrolysis was conducted in an undivided glass electrolyzer (85 cm^3^) equipped with a thermostatic jacket, a magnetic stirrer, a concentrically arranged, cylindrical Pt mesh anode (47 cm^2^) and cathode (44 cm^2^). To the thus prepared electrolyzer, *N*-acyl-α-amino acid (3.0 mmol), SiO_2_-Pip (200 mg), and methanol (30 cm^3^) were added. The electrochemical decarboxylative α-methoxylation was executed while stirring, at a constant current of 0.15 A, at 10 °C until a 3.75 F/mol charge had passed. Then, SiO_2_-Pip was filtered off, and methanol was evaporated under reduced pressure to obtain *N*-(1-methoxyalkyl)amide, which was used in the next reaction without further purification.

*Transformation of N-(1-methoxyalkyl)amides to 1-(N-acylamino)alkyltriarylphosphonium salts* **4** *[2,10]*

To a solution of triarylphosphine (1 mmol) in DCM (2 mL), a tetrafluoroboric acid diethyl ether complex (HBF_4_·Et_2_O, 136 µl, 161.9 mg, 1 mmol) was added at 0 °C. The reaction mixture was stirred for 2 h at room temperature. Thereafter, *N*-(1-methoxyalkyl)amide (1 mmol) was added and stirring was continued for 15 min. Evaporation of the solvent yielded the crude 1-(*N*-acylamino) alkyltriarylphosphonium salt **4**, which was used in the next reaction without further purification.

#### 3.2.2. Reactivity of 1-(*N*-acylamino)alkyltriarylphosphonium Salts **4** toward Carbon Nucleophiles

*Reaction of 1-(N-acylamino)alkyltriarylphosphonium salts* **4** *with C-H acids—general procedure*

A solution of a nucleophile (1.6 mmol), THF (0.5 mmol), and base LDA (2.0 M solution in THF, 0.1 cm^3^) was stirred under argon atmosphere. After a few minutes, this mixture was relegated to a solution of 1-(*N*-acylamino)alkyltriarylphosphonium salt (0.2 mmol) in THF (0.5 cm^3^) under argon atmosphere. The resulting reaction mixture was stirred for 15 min at room temperature, and then it was evaporated under reduced pressure. The product was separated using column chromatography [toluene/AcOEt 5:1 *v*/*v*].

*Diethyl 1-(pivaloylamino)etylpropanedioate* (**6a**) [10]. Colorless oil (38.5 mg, 67% yield). ^1^H-NMR (CDCl_3_) δ 6.80 (d, *J* = 7.5 Hz, 1H, NH), 4.74–4.62 (m, 1H, C_α_H), 4.29–4.21 (m, 2H, OCH_2_), 4.21–4.12 (m, 2H, OCH_2_), 3.57 (d, *J* = 3.4 Hz, 1H, CH), 1.32–1.25 (m, 9H, CH_3_ and 2 × OCH_2_CH_3_), 1.17 (s, 9H, *t*-Bu) ppm; ^13^C-NMR (CDCl_3_) δ 177.6 (NHC = O), 168.7 (C = O), 167.7 (C = O), 61.7 (OCH_2_), 61.5 (OCH_2_), 55.6 (C_α_H), 44.2 (CH), 38.6 (C(CH_3_)_3_), 27.4 (C(CH_3_)_3_), 19.0 (CH_3_), 14.0 (OCH_2_CH_3_) ppm; IR (ATR) 2977, 1731, 1647, 1510, 1175 cm^−1^.

*Ethyl 2-acetyl-3-(pivaloylamino)butanoate* (**6b**). A mixture of two disatereoisomers in a molar ratio of 1.4:1 (31.9 mg, 62% yield). ^1^H-NMR (CDCl_3_) δ 6.92 (d, *J* = 8.0 Hz, 1H, NH)^a^, 6.41 (d, *J* = 7.8 Hz, 1H, NH)^a^, 4.85–4.74 (m, 1H, C_α_H)^a^, 4.68–4.57 (m, 1H, C_α_H)^a^, 4.33–4.10 (m, 2H, OCH_2_)^b^, 3.72 (d, *J* = 4.9 Hz, 1H, CH)^a^, 3.65 (d, *J* = 3.6 Hz, 1H, CH)^a^, 2.26 (s, 3H, CH_3_CO)^a^, 2.25 (s, 3H, CH_3_CO)^a^, 1.35–1.20 (m, 6H, CH_3_ and OCH_2_CH_3_)^b^, 1.16 (s, 9H, *t*-Bu)^a^, 1.15 (s, 9H, *t*-Bu)^a^ ppm; ^13^C-NMR (CDCl_3_) δ 203.4^a^ (CH_3_C = O), 202.1^a^ (CH_3_C = O), 178.0^a^ (NHC = O), 177.7^a^ (NHC = O), 169.5^a^ (C = O), 168.2^a^ (C = O), 63.4^a^ (C_α_H), 62.2^a^ (C_α_H), 61.6^a^ (OCH_2_), 61.4^a^ (OCH_2_), 44.3^a^ (CH), 43.4^a^ (CH), 38.6^a^ (C(CH_3_)_3_), 38.6^a^ (C(CH_3_)_3_), 30.4^a^ (CH_3_CO), 29.0^a^ (CH_3_CO), 27.4^a^ (C(CH_3_)_3_), 27.3^a^ (C(CH_3_)_3_), 19.7^a^ (CH_3_), 18.7^a^ (CH_3_), 14.1^a^ (OCH_2_CH_3_), 14.1^a^ (OCH_2_CH_3_) ppm; IR (ATR) 3318, 2971, 1730, 1635, 1534, 1301, 1190 cm^−1^. HRMS (TOF-ESI) calcd for C_13_H_24_NO_4_ [M + H]^+^ 258.1705, found 258.1701. ^a^ Separate signals from both diastereoisomers. ^b^ Overlapping signals of both diastereoisomers.

*Ethyl 2-acetyl-5-methyl-3-(phenylacetylamino)hexanoate* (**6c**). A mixture of two disatereoisomers in a molar ratio of 1.25:1 (55.4 mg, 83% yield). ^1^H-NMR (CDCl_3_) δ 7.39–7.19 (m, 5H, Ph)^a^, 6.43 (d, *J* = 9.7 Hz, 1H, NH)^b^, 6.02 (d, *J* = 9.5 Hz, 1H, NH)^b^, 4.77–4.68 (m, 1H, C_α_H)^b^, 4.63–4.54 (m, 1H, C_α_H)^b^, 4.21–4.07 (m, 2H, OCH_2_)^a^, 3.64 (d, *J* = 4.9 Hz, 1H, CH)^b^, 3.60 (d, *J* = 3.7 Hz, 1H, CH)^b^, 3.51 (s, 2H, PhCH_2_)^a^, 2.23 (s, 3H, CH_3_CO)^b^, 2.19 (s, 3H, CH_3_CO)^b^, 1.56–1.37 (m, 2H, CH_2_)^a^, 1.28–1.14 (m, 4H, CH and OCH_2_CH_3_)^a^, 0.95–0.80 (m, 6H, 2 × CH_3_)^a^ ppm; ^13^C-NMR (CDCl_3_) δ 203.2^b^ (CH_3_C = O), 202.2^b^ (CH_3_C = O), 170.6^b^ (NHC = O), 170.4^b^ (NHC = O), 169.1^b^ (C = O), 168.1^b^ (C = O), aromatic carbons: 134.7^b^, 134.6^b^, 129.3^b^, 129.3^b^, 128.9^b^, 128.8^b^, 127.2^b^, 127.2^b^, 62.4^b^ (C_α_H), 61.8^b^ (C_α_H), 61.6^b^ (OCH_2_), 61.3^b^ (OCH_2_), 47.1^b^ (PhCH_2_), 46.2^b^ (PhCH_2_), 43.9^b^ (CH), 43.8^b^ (CH), 42.7^b^ (CH_2_), 41.9^b^ (CH_2_), 30.3^b^ (CH_3_CO), 29.1^b^ (CH_3_CO), 25.1^b^ (CH), 25.1^b^ (CH), 23.1^b^(CH_3_), 22.7^b^ (CH_3_), 22.1^b^ (CH_3_), 21.7^b^ (CH_3_), 14.0^b^ (OCH_2_CH_3_), 14.0^b^ (OCH_2_CH_3_) ppm; IR (ATR) 3275, 2956, 1740, 1648, 1496, 1260, 1144 cm^−1^. HRMS (TOF-ESI) calcd for C_19_H_28_NO_4_ [M + H]^+^ 334.2018, found 334.2009. ^a^ Overlapping signals of both diastereoisomers. ^b^ Separate signals from both diastereoisomers.

*Dimethyl 3-methyl-1-(phenylacetylamino)butylpropanedioate* (**6d**)*.* Colorless crystals (42.3 mg, 63% yield), mp 78–80 °C. ^1^H-NMR (CDCl_3_) δ 7.41–7.18 (m, 5H, Ph), 6.25 (d, *J* = 9.6 Hz, 1H, NH), 4.68–4.59 (m, 1H, C_α_H), 3.69 (s, 3H, OCH_3_), 3.62 (s, 3H, OCH_3_), 3.55 (d, *J* = 3.9 Hz, 1H, CH), 3.52 (s, 2H, PhCH_2_), 1.54–1.39 (m, 2H, CH_2_), 1.28–1.19 (m, 1H, CH), 0.90 (d, *J* = 6.5 Hz, 3H, CH_3_), 0.86 (d, *J* = 6.6 Hz, 3H, CH_3_) ppm; ^13^C-NMR (CDCl_3_) δ 170.3 (NHC = O), 168.6 (C = O), 168.0 (C = O), aromatic carbons: 134.8, 129.3, 128.8, 127.1, 54.6 (C_α_H), 52.6 (OCH_3_), 52.3 (OCH_3_), 46.8 (PhCH_2_), 43.9 (CH), 42.1 (CH_2_), 25.0 (CH), 22.9 (CH_3_), 21.9 (CH_3_) ppm; IR (ATR) 3283, 2954, 1736, 1648, 1454, 1263 cm^−1^. HRMS (TOF-ESI) calcd for C_18_H_26_NO_5_ [M + H]^+^ 336.1811, found 336.1805.

*Diethyl 3-methyl-1-(phenylacetylamino)butylpropanedioate* (**6e**)*.* Colorless crystals (37.8 mg, 52% yield), mp 93.5–95.5 °C. ^1^H-NMR (CDCl_3_) δ 7.42–7.19 (m, 5H, Ph), 6.30 (d, *J* = 9.6 Hz, 1H, NH), 4.68–4.60 (m, 1H, C_α_H), 4.30–4.00 (m, 4H, 2 × OCH_2_), 3.52 (s, 2H, PhCH_2_), 3.52 (d, *J* = 2.8 Hz, 1H, CH), 1.57–1.37 (m, 2H, CH_2_), 1.36–1.06 (m, 7H, CH and 2 × OCH_2_CH_3_), 0.90 (d, *J* = 6.5 Hz, 3H, CH_3_), 0.86 (d, *J* = 6.6 Hz, 3H, CH_3_) ppm; ^13^C-NMR (CDCl_3_) δ 170.2 (NHC = O), 168.3 (C = O), 167.7 (C = O), aromatic carbons: 134.9, 129.3, 128.8, 127.1, 61.7 (OCH_2_), 61.4 (OCH_2_), 54.9 (C_α_H), 46.8 (PhCH_2_), 43.9 (CH), 42.2 (CH_2_), 25.0 (CH), 22.9 (CH_3_), 22.0 (CH_3_), 14.0 (OCH_2_CH_3_), 13.9 (OCH_2_CH_3_) ppm; IR (ATR) 3362, 2964, 1745, 1656, 1533, 1345, 1144 cm^−1^. HRMS (TOF-ESI) calcd for C_20_H_30_NO_5_ [M + H]^+^ 364.2124, found 364.2122.

*Reaction of 1-(N-acylamino)alkyltriarylphosphonium salts* **4** *with 1-morpholinocyclohexene—general procedure*

To a solution of 1-(*N*-acylamino)alkyltriarylphosphonium salt **4** (0.25 mmol) in MeCN (1 cm^3^), 1-morpholinocyclohexene (84.1 µL, 83.7 mg, 0.5 mmol) was added. After 1 h of stirring at room temperature, an aqueous solution of citric acid (20%) (1.125 cm^3^) was added. Stirring was continued for 45 min, and then a saturated solution of KHCO_3_ was added. Thereafter, the mixture was extracted with DCM (5 × 3 cm^3^), and the organic layer was combined and dried over MgSO_4_. Then, the solvent was evaporated under reduced pressure and the crude product was purified using column chromatography [toluene/EtOAc 2:1 *v*/*v* (**8a**), 5:1 (**8b**, **8c**, **8e**) or 10:1 *v*/*v* (**8d**)].

*N-[1-(2-oxocyclohexyl)ethyl]pivalamide* (**8a**)*.* A mixture of two disatereoisomers in a molar ratio of 3.5:1 (35.5 mg, 63% yield). The major diastereoisomers was isolated using column chromatography [toluene/EtOAc 2:1] and crystallization from toluene. Colorless crystals, mp 124–126 °C. ^1^H-NMR (CDCl_3_) δ 6.51 (d, *J* = 8.1 Hz, 1H, NH), 4.19–4.10 (m, 1H, C_α_H), 2.56–2.48 (m, 1H, CH), 2.39–2.30 (m, 2H, CH_2_), 2.11–2.01 (m, 2H, CH_2_), 1.91–1.83 (m, 1H, CHH), 1.74–1.57 (m, 3H, CHH and CH_2_), 1.22 (d, *J* = 7.0 Hz, 3H, CH_3_), 1.17 (s, 9H, *t*-Bu) ppm; ^13^C-NMR (CDCl_3_) δ 214.2 (C = O), 178.0 (NHC = O), 55.4 (C_α_H), 45.6 (CH), 43.1 (CH_2_), 38.7 (C(CH_3_)_3_), 32.7 (CH_2_), 28.4 (CH_2_), 27.6 (C(CH_3_)_3_), 24.8 (CH_2_), 20.0 (CH_3_) ppm; IR (ATR) 3339, 2961, 2869, 1706, 1627, 1526, 1305, 1209, 1117 cm^−1^. HRMS (TOF-ESI) calcd for C_13_H_24_NO_2_ [M + H]^+^ 226.1807, found 226.1800.

*N-[1-(2-oxocyclohexyl)-3-methylbutyl]phenylacetamide* (**8b**)*.* A mixture of two disatereoisomers in a molar ratio of 3.2:1 (46.7 mg, 62% yield). The major diastereoisomers was isolated using column chromatography [toluene/EtOAc 5:1] and crystallization from toluene. Colorless crystals, mp 115.5–117.5 °C. ^1^H-NMR (CDCl_3_) δ 7.42–7.09 (m, 5H, Ph), 6.13 (d, *J* = 9.7 Hz, 1H, NH), 4.10–3.99 (m, 1H, C_α_H), 3.51 (s, 2H, PhCH_2_), 2.49–2.40 (m, 1H, CH), 2.31–2.18 (m, 2H, CH_2_), 2.07–1.96 (m, 2H, CH_2_), 1.86–1.75 (m, 1H, CH), 1.65–1.37 (m, 5H, CH and 2 × CH_2_), 1.21–1.09 (m, 1H, CH), 0.86 (d, *J* = 6.5 Hz, 3H, CH_3_), 0.83 (d, *J* = 6.6 Hz, 3H, CH_3_) ppm; ^13^C-NMR (CDCl_3_) δ 213.5 (C = O), 170.7 (NHC = O), aromatic carbons: 135.3, 129.1, 128.8, 127.1, 54.8 (C_α_H), 48.7 (PhCH_2_), 44.2 (CH), 43.1 (CH_2_), 43.1 (CH_2_), 32.6 (CH_2_), 28.3 (CH_2_), 25.2 (CH), 25.0 (CH_2_), 23.1 (CH_3_), 22.0 (CH_3_) ppm; IR (ATR) 3273, 2954, 2865, 1702, 1633, 1552, 1455, 1315, 1138 cm^−1^. HRMS (TOF-ESI) calcd for C_19_H_28_NO_2_ [M + H]^+^ 302.2120, found 302.2115.

*Benzyl N-[1-(2-oxocyclohexyl)-3-methylbutyl]carbamate* (**8c**)*.* Only one disatereoisomer was detected and isolated. Colorless oil (31.7 mg, 40% yield). ^1^H-NMR (CDCl_3_) δ 7.42–7.27 (m, 5H, Ph), 5.42 (d, *J* = 10.1 Hz, 1H, NH), 5.09 (d, *J* = 12.5 Hz, 1H, PhCHHO), 5.06 (d, *J* = 12.4 Hz, 1H, PhCHHO), 3.83–3.69 (m, 1H, C_α_H), 2.54–2.44 (m, 1H, CH), 2.38–2.23 (m, 2H, CH_2_), 2.15–1.99 (m, 2H, CH_2_), 1.93–1.82 (m, 1H, CHH), 1.74–1.55 (m, 5H, CHH and 2 × CH_2_), 1.28–1.16 (m, 1H, CH), 0.91 (d, *J* = 6.4 Hz, 3H, CH_3_), 0.89 (d, *J* = 6.5 Hz, 3H, CH_3_) ppm; ^13^C-NMR (CDCl_3_) δ 213.1 (C = O), 156.5 (NHC = O), aromatic carbons: 136.8, 128.4, 127.9, 127.9, 66.5 (PhCH_2_O), 54.7 (C_α_H), 50.8 (CH), 43.4 (CH_2_), 43.2 (CH_2_), 32.3 (CH_2_), 28.1 (CH_2_), 25.1 (CH_2_), 25.1 (CH), 23.2 (CH_3_), 21.9 (CH_3_) ppm; IR (ATR) 3439, 3333, 2952, 2866, 1699, 1499, 1213, 1052 cm^−1^. HRMS (TOF-ESI) calcd for C_19_H_28_NO_3_ [M + H]^+^ 318.2069, found 318.2061.

*Benzyl N-[1-(2-oxocyclohexyl)-2-phenylethyl]carbamate* (**8d**)*.* A mixture of two disatereoisomers in a molar ratio of 13.4:1 (29.0 mg, 33% yield). The major diastereoisomers was isolated using column chromatography [toluene/EtOAc 10:1]. Colorless crystals, mp 99–101 °C. ^1^H-NMR (CDCl_3_) δ 7.42–7.06 (m, 10H, 2 × Ph), 5.65 (d, *J* = 10.0 Hz, 1H, NH), 5.07 (d, *J* = 12.3 Hz, 1H, PhCHHO), 5.00 (d, *J* = 12.4 Hz, 1H, PhCHHO), 3.95–3.87 (m, 1H, C_α_H), 2.99 (dd, *J*_1_ = 13.5, *J*_2_ = 7.3 Hz, 1H, PhCHH), 2.92 (dd, *J*_1_ = 13.5, *J*_2_ = 8.5 Hz, 1H, PhCHH), 2.51–2.41 (m, 1H, CH), 2.40–2.31 (m, 1H, CH), 2.29–2.17 (m, 1H, CH), 2.09–1.92 (m, 2H, CH_2_), 1.87–1.78 (m, 1H, CH), 1.77–1.51 (m, 3H, CH and CH_2_) ppm; ^13^C-NMR (CDCl_3_) δ 213.5 (C = O), 156.4 (NHC = O), aromatic carbons: 138.6, 136.7, 129.1, 128.5, 128.4, 127.9, 127.8, 126.4, 66.4 (PhCH_2_O), 54.2 (C_α_H), 51.9 (CH), 43.2 (CH_2_), 40.3 (PhCH_2_), 32.6 (CH_2_), 28.1 (CH_2_), 25.0 (CH_2_) ppm; IR (ATR) 3308, 2929, 1721, 1697, 1541, 1341, 1242, 1094, 1051 cm^−1^. HRMS (TOF-ESI) calcd for C_22_H_26_NO_3_ [M + H]^+^ 352.1913, found 352.1913.

*Benzyl N-[1-(2-oxocyclohexyl)-2-tert-butoxyethyl]carbamate* (**8e**)*.* Only one disatereoisomer was detected and isolated. Colorless oil (26.9 mg, 31% yield). ^1^H-NMR (CDCl_3_) δ 7.38–7.28 (m, 5H, Ph), 5.57 (d, *J* = 9.7 Hz, 1H, NH), 5.11 (d, *J* = 12.3 Hz, 1H, PhCHHO), 5.06 (d, *J* = 12.3 Hz, 1H, PhCHHO), 3.89–3.79 (m, 1H, C_α_H), 3.44 (d, *J* = 7.1 Hz, 2H, CH_2_O*t*-Bu), 2.90–2.82 (m, 1H, CH), 2.38–2.27 (m, 2H, CH_2_), 2.09–2.00 (m, 2H, CH_2_), 1.92–1.85 (m, 1H, CHH), 1.78–1.64 (m, 3H, CHH and CH_2_), 1.12 (s, 9H, *t*-Bu) ppm; ^13^C-NMR (CDCl_3_) δ 213.8 (C = O), 156.4 (NHC = O), aromatic carbons: 136.6, 128.5, 128.0, 73.1 (OC(CH_3_)_3_), 66.6 (PhCH_2_O), 62.1 (CH_2_O*t*-Bu), 52.5 (C_α_H), 50.0 (CH), 42.8 (CH_2_), 31.9 (CH_2_), 28.1 (CH_2_), 27.5 (OC(CH_3_)_3_), 24.9 (CH_2_) ppm; IR (ATR) 3432, 2972, 2935, 2867, 1701, 1499, 1363, 1195, 1057 cm^−1^. HRMS (TOF-ESI) calcd for C_20_H_30_NO_4_ [M + H]^+^ 348.2175, found 348.2173.

#### 3.2.3. Reactivity of 1-(*N*-Acylamino)alkyltriarylphosphonium Salts **4** toward Heteronucleophiles

*Reaction of 1-(N-acylamino)alkyltriarylphosphonium salts* **4** *with benzotriazole sodium salt—general procedure*

To a solution of 1-(*N*-acylamino)alkyltriarylphosphonium salt **4** (0.5 mmol) in CHCl_3_ (1 cm^3^), benzotriazole sodium salt (0.5 mmol) was added. The reaction mixture was stirred at room temperature for 15 min. Thereafter, it was filtered through a fluted filter and the filtrate was evaporated under reduced pressure. The residue was crystallized from toluene.

*N-[1-(Benzotriazol-1-yl)ethyl]pivalamide* (**10a**) [6]. Colorless crystals (121.9 mg, 99% yield), mp 142–144 °C. ^1^H-NMR (CDCl_3_) δ 8.06–8.02 (m, 1H, aromatic), 7.85–7.79 (m, 1H, aromatic), 7.54–7.48 (m, 1H, aromatic), 7.41–7.35 (m, 1H, aromatic), 6.88 (dq, *J*_1_ = 9.1, *J*_2_ = 6.7 Hz, 1H, C_α_H), 6.63 (d, *J* = 8.8 Hz, 1H, NH), 2.04 (d, *J* = 6.7 Hz, 3H, CH_3_), 1.15 (s, 9H, *t*-Bu) ppm; ^13^C-NMR (100 MHz, CDCl_3_) δ 178.2 (C = O), aromatic carbons: 145.5, 132.4, 127.7, 124.4, 119.5, 110.4, 58.7 (C_α_H), 38.7 (C(CH_3_)_3_), 27.2 (C(CH_3_)_3_), 20.7 (CH_3_) ppm; IR (ATR) 3346, 2969, 1668, 1512, 1193, 1152, 1065 cm^−1^.

*Benzyl N-[1-(benzotriazol-1-yl)-2-phenylethyl]carbamate* (**10b**) [6]. Colorless crystals (130.4 mg, 70% yield), mp 117.5–119.5 °C. ^1^H-NMR (CDCl_3_) δ 8.00 (d, *J* = 8.4 Hz, 1H, aromatic), 7.54 (d, *J* = 7.5 Hz, 1H, aromatic), 7.42–7.35 (m, 1H, aromatic), 7.33–7.27 (m, 4H, aromatic), 7.27–7.20 (m, 2H, aromatic), 7.19–7.12 (m, 3H, aromatic), 7.11–7.04 (m, 2H, aromatic), 6.71–6.58 (m, 1H, C_α_H), 6.02 (d, *J* = 8.9 Hz, 1H, NH), 5.10 (d, *J* = 12.4 Hz, 1H, PhCHHO), 4.97 (d, *J* = 12.2 Hz, 1H, PhCHHO), 3.80–3.68 (m, 1H, PhCHH), 3.63 (dd, *J*_1_ = 13.8, *J*_2_ = 6.6 Hz, 1H, PhCHH) ppm; ^13^C-NMR (CDCl_3_) δ 160.3 (C = O), aromatic carbons: 145.5, 136.0, 134.7, 131.9, 129.1, 128.7, 128.5, 128.4, 128.1, 127.7, 127.3, 124.1, 119.7, 109.7, 67.5 (PhCH_2_O), 65.8 (C_α_H), 41.1 (PHCH_2_) ppm; IR (ATR) 3177, 3008, 1712, 1548, 1280, 1261, 1244, 1195, 1046, 1022 cm^−1^.

*Reaction of 1-(N-pivaloylamino)ethyltris(4-trifluoromethylphenyl)phosphonium tetrafluoroborate* **4a** *with sodium p-toluenesulfinate*

To a solution of 1-(*N*-pivaloylamino)ethyltris(4-trifluoromethylphenyl)phosphonium tetrafluoroborate **4a** (340.7 mg, 0.5 mmol) in CHCl_3_ (1 cm^3^), sodium *p*-toluenesulfinate (89.1 mg, 0.5 mmol) was added. The reaction mixture was stirred at room temperature for 15 min. Thereafter, it was filtered through a fluted filter and the filtrate was evaporated under reduced pressure. The residue was crystallized from toluene.

*N-[1-(p-Toluenesulfonyl)ethyl]pivalamide* (**10c**) [5]. Colorless crystals (124.7 mg, 88% yield), mp 143–145 °C. ^1^H-NMR (CDCl_3_) δ 7.77 (d, *J* = 8.3 Hz, 2H, aromatic), 7.33 (d, *J* = 7.9 Hz, 2H, aromatic), 5.98 (d, *J* = 10.2 Hz, 1H, NH), 5.41 (dq, *J*_1_ = 10.2, *J*_2_ = 7.0 Hz, 1H, C_α_H), 2.42 (s, 3H, CH_3_), 1.62 (d, *J* = 7.0 Hz, 3H, CH_3_), 1.01 (s, 9H, *t*-Bu) ppm; ^13^C-NMR (CDCl_3_) δ 176.9 (C = O), aromatic carbons: 145.1, 133.5, 129.6, 129.1, 64.3 (C_α_H), 38.7 (C(CH_3_)_3_), 27.2 (C(CH_3_)_3_), 21.6 (CH_3_), 13.2 (CH_3_) ppm; IR (ATR) 3372, 2973, 1677, 1516, 1308, 1287, 1135, 1083, 1016, 724 cm^−1^.

*Reaction of 1-(N-acylamino)alkyltriarylphosphonium salts* **4** *with benzylamine—general procedure*

To a stirred solution of 1-(*N*-acylamino)alkyltriarylphosphonium salt **4** (0.25 mmol) in DCM (1 cm^3^), benzylamine (109.4 µl, 110 mg, 1 mmol) was added dropwise. Stirring was continued for 5 min at room temperature. Thereafter, the mixture was evaporated under reduced pressure and dried. The residue was purified using column chromatography [DCM/MeOH/Et_3_N, 5:1:0.2 *v*/*v*/*v*].

*N-[1-(Benzylamino)ethyl]pivalamide* (**10d**)*.* Yellow crystals (53.3 mg, 91% yield), mp 56.5–58.5 °C. ^1^H-NMR (CDCl_3_) δ 7.35–7.29 (m, 4H, aromatic), 7.26–7.21 (m, 1H, aromatic), 5.67 (d, *J* = 6.7 Hz, 1H, NH), 4.90 (qd, *J*_1_ = 6.2, *J*_2_ = 1.6 Hz, 1H, C_α_H), 3.77 (s, 2H, PhCH_2_), 1.92 (br s, 1H, NH), 1.32 (d, *J* = 6.2 Hz, 3H, CH_3_), 1.17 (s, 9H, *t*-Bu) ppm; ^13^C-NMR (CDCl_3_) δ 178.1 (C = O), aromatic carbons: 140.1, 128.4, 127.9, 126.9, 60.9 (C_α_H), 49.8 (PhCH_2_), 38.6 (C(CH_3_)_3_), 27.4 (C(CH_3_)_3_), 21.6 ppm (CH_3_); IR (ATR) 3328, 2971, 1622, 1527, 1475, 1144, 1097 cm^−1^. HRMS (TOF-ESI) calcd for C_14_H_23_N_2_O [M + H]^+^ 235.1810, found 235.1802.

*N-[1-(Benzylamino)-3-methylbutyl]phenylacetamide* (**10e**)*.* Colorless crystals (42.7 mg, 55% yield), mp 93–95 °C. ^1^H-NMR (CDCl_3_) δ 7.59–6.96 (m, 10H, 2 × Ph), 5.33 (d, *J* = 8.3 Hz, 1H, NH), 4.82–4.75 (m, 1H, C_α_H), 3.68 (s, 2H, PhCH_2_NH), 3.54 (s, 2H, PhCH_2_), 1.66 (br s, 1H, NH), 1.64–1.53 (m, 1H, CH), 1.45–1.28 (m, 2H, CH_2_), 0.87 (d, *J* = 6.6 Hz, 3H, CH_3_), 0.84 (d, *J* = 6.6 Hz, 3H, CH_3_) ppm; ^13^C-NMR (CDCl_3_) δ 170.9 (C = O), aromatic carbons: 140.3, 135.0, 129.3, 129.0, 128.4, 128.0, 127.3, 126.9, 63.3 (C_α_H), 49.6 (PhCH_2_), 44.5 (PhCH_2_), 44.1 (CH_2_), 24.9 (CH), 22.6 (CH_3_), 22.5 (CH_3_) ppm; IR (ATR) 3306, 2962, 2946, 1632, 1518, 1144, 1009 cm^−1^. HRMS (TOF-ESI) calcd for C_20_H_27_N_2_O [M + H]^+^ 311.2123, found 311.2121.

*Reaction of 1-(N-pivaloylamino)ethyltris(4-trifluoromethylphenyl)phosphonium tetrafluoroborate* **4a** *with triphenylphosphine*

To a solution of 1-(*N*-pivaloylamino)ethyltris(4-trifluromethylphenyl)phosphonium tetrafluoroborate **4a** (102.2 mg, 0.15 mmol) in DCM (1 cm^3^), triphenylphosphine (39.3 mg, 0.15 mmol) was added. The homogeneous mixture was allowed to react at room temperature for 5 min, and 1-(*N*-pivaloylamino)ethyltriphenylphosphonium tetrafluoroborate (**4b**) [9] was precipitated with Et_2_O, separated by decantation, and dried under reduced pressure.

1-(*N*-Pivaloylamino)ethyltriphenylphosphonium tetrafluoroborate (**4b**). Colorless crystals (70.2 mg, 98% yield), mp 160.5–162.5 °C. ^1^H-NMR (CDCl_3_) δ 7.89–7.64 (m, 16H, 3 × Ph and NH), 5.82–5.73 (m, 1H, C_α_H), 1.72 (dd, *J*_1_ = 17.8, *J*_2_ = 7.4 Hz, 3H, CH_3_), 0.91 (s, 9H, *t*-Bu) ppm; ^13^C-NMR (CDCl_3_) δ 179.7 (d, *J* = 2.3 Hz, C = O), aromatic carbons: 134.8 (d, *J* = 3.0 Hz), 134.5 (d, *J* = 9.2 Hz), 130.0 (d, *J* = 12.3 Hz), 118.4 (d, *J* = 82.5 Hz), 45.0 (d, *J* = 53.4 Hz, C_α_H), 38.5 (C(CH_3_)_3_), 26.8 (C(CH_3_)_3_), 17.4 (d, *J* = 4.6 Hz, CH_3_) ppm; ^31^P NMR (161.9 MHz, CDCl_3_) δ 29.2 ppm; IR (ATR) 3373, 1684, 1516, 1447, 1136, 1040 cm^−1^.

*Reaction of 1-(N-acylamino)alkyltriarylphosphonium salts* **4** *with triarylphosphines—NMR scale*

To a solution of 1-(*N*-acylamino)alkyltriarylphosphonium salt (0.025 mmol) in CDCl_3_ (0.65 cm^3^), the corresponding triarylphosphine (0.025 mmol) was added. Reactions were conducted in NMR tubes at 26 °C, and their course was monitored using NMR spectroscopy.

*Reaction of 1-(N-acylamino)alkyltriarylphosphonium salts* **4** *with phosphorus nucleophiles in Michaelis–Arbuzov type reaction—general procedure*

To a stirred solution of 1-(*N*-acylamino)alkyltriarylphosphonium salt **4** (0.25 mmol) and methyltriphenylphosphonium iodide (25.3 mg, 0.0625 mmol) in DCM (2 cm^3^), phosphorus nucleophile (0.375 mmol) was added, and the mixture was stirred for 3 h at room temperature (**12a**–**c** and **12e**) or for 2 h at 60 °C (**12d**). The mixture was evaporated under reduced pressure and the residue was extracted with toluene (3 × 1 cm^3^) at 50 °C. The solvent was evaporated under reduced pressure, and the product was isolated using column chromatography [DCM/MeOH 20:1 *v*/*v*].

*Dimethyl 1-(N-pivaloylamino)ethanephosphonate* (**12a**) [7]. Colorless crystals (50.4 mg, 85% yield), mp 125.5–127.5 °C. ^1^H-NMR (CDCl_3_) δ 5.96 (d, *J* = 8.1 Hz, 1H, NH), 4.65–4.49 (m, 1H, C_α_H), 3.78 (d, *J* = 4.4 Hz, 3H, OCH_3_), 3.75 (d, *J* = 4.3 Hz, 3H, OCH_3_), 1.38 (dd, *J*_1_ = 16.8, *J*_2_ = 7.4 Hz, 3H, CH_3_), 1.21 (s, 9H, *t*-Bu) ppm; ^13^C-NMR (CDCl_3_) δ 177.6 (d, *J* = 5.1 Hz, C = O), 53.1 (d, *J* = 7.0 Hz, OCH_3_), 52.9 (d, *J* = 6.5 Hz, OCH_3_), 40.1 (d, *J* = 156.4 Hz, C_α_H), 38.6 (C(CH_3_)_3_), 27.3 (C(CH_3_)_3_), 15.5 (CH_3_) ppm; ^31^P-NMR (CDCl_3_) δ 28.5 ppm; IR (ATR) 3281, 2956, 1659, 1529, 1224, 1205, 1057, 1026, 1007 cm^−1^.

*Dimethyl 1-(N-phenylcetylamino)-3-methylbutanephosphonate* (**12b**) [4]. Colorless crystals (60.3 mg, 77% yield), mp 117–119 °C. ^1^H-NMR (CDCl_3_) δ 7.39–7.23 (m, 5H, Ph), 5.72 (d, *J* = 9.9 Hz, 1H, NH), 4.62–4.48 (m, 1H, C_α_H), 3.73 (d, *J* = 10.6 Hz, 3H, OCH_3_), 3.63 (d, *J* = 10.6 Hz, 3H, OCH_3_), 3.60 (s, 2H, PhCH_2_), 1.61–1.44 (m, 3H, CH_2_ and CH), 0.88 (d, *J* = 5.9 Hz, 6H, 2 × CH_3_) ppm; ^13^C-NMR (CDCl_3_) δ 170.5 (d, *J* = 4.3 Hz, C = O), aromatic carbons: 134.6, 129.2, 128.9, 127.4, 53.1 (d, *J* = 7.2 Hz, OCH_3_), 53.0 (d, *J* = 6.6 Hz, OCH_3_), 43.7 (PhCH_2_), 43.2 (d, *J* = 155.5 Hz, C_α_H), 38.1 (d, *J* = 1.8 Hz, CH_2_), 24.5 (d, *J* = 13.4 Hz, CH), 23.2 (CH_3_), 21.1 (CH_3_) ppm; ^31^P-NMR (CDCl_3_) δ 27.6 ppm; IR (ATR) 3243, 2959, 1673, 1540, 1217, 1027, 836, 742 cm^−1^.

*Dimethyl 1-(N-benzyloxycarbonylamino)-2-tert-butoxyethanephosphonate* (**12c**) [4]. Colorless crystals (54.8 mg, 61% yield), mp 59.5–61.5 °C. ^1^H-NMR (CDCl_3_) δ 7.41–7.28 (m, 5H, Ph), 5.29 (d, *J* = 8.8 Hz, 1H, NH), 5.13 (s, 2H, PhCH_2_O), 4.33–4.19 (m, 1H, C_α_H), 3.76 (d, *J* = 10.6 Hz, 3H, OCH_3_), 3.75 (d, *J* = 10.3 Hz, 3H, OCH_3_), 3.61 (dd, *J*_1_ = 9.4, *J*_2_ = 3.8 Hz, 1H, CHHO*t*-Bu), 3.54 (dd, *J*_1_ = 9.4, *J*_2_ = 3.8 Hz, 1H, CHHO*t*-Bu), 1.18 (s, 9H, *t*-Bu) ppm; ^13^C-NMR (CDCl_3_) δ 155.7 (C = O), aromatic carbons: 136.2, 128.5, 128.2, 128.1, 73.7 (OC(CH_3_)_3_), 67.2 (PhCH_2_O), 60.6 (CH_2_O*t*-Bu), 53.3 (d, *J* = 6.0 Hz, OCH_3_), 52.6 (d, *J* = 5.8 Hz, OCH_3_), 48.3 (d, *J* = 156.0 Hz, C_α_H), 27.3 (OC(CH_3_)_3_) ppm; ^31^P-NMR (CDCl_3_) δ 26.1 ppm; IR (ATR) 3293, 2974, 1699, 1532, 1278, 1235, 1039, 1023, 756, 697 cm^−1^.

*Methyl phenyl(1-pivaloylaminoethyl)phosphinate* (**12d**). Colorless crystals (48.9 mg, 69% yield), mp 131–133 °C. ^1^H-NMR (CDCl_3_) δ 7.84–7.75 (m, 2H, aromatic), 7.59–7.43 (m, 3H, aromatic), 5.76 (d, *J* = 9.6 Hz, 1H, NH), 4.79–4.67 (m, 1H, C_α_H), 3.67 (d, *J* = 10.8 Hz, 3H, OCH_3_), 1.45 (dd, *J*_1_ = 14.6, *J*_2_ = 7.3 Hz, 3H, CH_3_), 0.92 (s, 9H, *t*-Bu) ppm; ^13^C-NMR (CDCl_3_) δ 177.3 (d, *J* = 4.7 Hz, C = O), aromatic carbons: 132.8 (d, *J* = 2.8 Hz), 132.4 (d, *J* = 9.4 Hz), 128.5 (d, *J* = 12.5 Hz), 127.5 (d, *J* = 124.2 Hz), 51.8 (d, *J* = 7.1 Hz, OCH_3_), 42.3 (d, *J* = 115.5 Hz, C_α_H), 38.5 (C(CH_3_)_3_), 27.1 (C(CH_3_)_3_), 14.4 (CH_3_) ppm; ^31^P-NMR (CDCl_3_) δ 42.9 ppm. IR (ATR) 3264, 2954, 1660, 1531, 1198, 1139, 1027, 799 cm^−1^; HRMS (TOF-ESI) calcd for C_14_H_23_NO_3_P [M + H]^+^ 284.1416, found 284.1405.

*Diphenyl 1-(N-benzyloxycarbonylamino)-3-methylbutylphosphine oxide* (**12e**) [8]. Colorless crystals (87.5 mg, 83% yield), mp 173.5–175.5 °C. ^1^H-NMR (CDCl_3_) δ 7.87–7.09 (m, 15H, 3 × Ph), 5.40 (d, *J* = 10.6 Hz, 1H, NH), 5.02 (d, *J* = 12.5 Hz, 1H, PhCHHO), 4.89 (d, *J* = 12.5 Hz, 1H, PhCHHO), 4.83–4.72 (m, 1H, C_α_H), 1.83–1.66 (m, 2H, CH_2_), 1.36–1.21 (m, 1H, CH), 0.90 (d, *J* = 6.5 Hz, 3H, CH_3_), 0.86 (d, *J* = 6.6 Hz, 3H, CH_3_) ppm; ^13^C-NMR (CDCl_3_) δ 156.1 (d, *J* = 4.5 Hz, C = O), aromatic carbons: 136.4, 132.0 (d, *J* = 2.8 Hz), 131.9 (d, *J* = 2.8 Hz), 131.5, 131.3, 131.1 (d, *J* = 9.2 Hz), 130.9 (d, *J* = 9.0 Hz), 128.8 (d, *J* = 11.4 Hz), 128.4 (d, *J* = 11.6 Hz), 128.4, 127.9, 127.6, 66.8 (PhCH_2_O), 48.1 (d, *J* = 79.5 Hz, C_α_H), 37.5 (d, *J* = 3.5 Hz, CH_2_), 24.5 (d, *J* = 11.0 Hz, CH), 23.4 (CH_3_), 21.0 (CH_3_) ppm; ^31^P-NMR (CDCl_3_) δ 33.5 ppm; IR (ATR) 3181, 2954, 1702, 1545, 1436, 1263, 1187, 1120 cm^−1^.

*Reaction of 1-(N-pivaloylamino)ethyltris(3-chlorophenyl)phosphonium tetrafluoroborate* **4c** *with trimethyl phosphite—measurement of changes in concentrations of the substrate* **4c** *, intermediate* **13a** *and product* **12a** *by NMR*

To a solution of 1-(*N*-pivaloylamino)ethyltris(3-chlorophenyl)phosphonium tetrafluoroborate **4c** (29.0 mg, 0.05 mmol) in CDCl_3_ (0.65 cm^3^), trimethyl phosphite (8.9 µL, 9.3 mg, 0.075 mmol) was added. Dimethyldiphenylsilane (5 mg) was used as the internal standard. The reaction mixture was placed directly into the NMR tube. Changes in the concentrations of substrate **4c**, intermediate **13a**, and product **12a** were monitored using ^1^H-NMR spectroscopy.

*Reaction of 1-(N-pivaloylamino)ethyltris(3-chlorophenyl)phosphonium tetrafluoroborate* **4c** *with triethyl phosphite–synthesis of 1-(N-pivaloylamino)ethyltriethoxyphosphonium tetrafluoroborate* **13b**

To a stirred solution of 1-(*N*-pivaloylamino)ethyltris(3-chlorophenyl)phosphonium tetrafluoroborate (**4c**, 145.2 mg, 0.25 mmol) in DCM (1 cm^3^), triethyl phosphite (64.6 µL, 62.3 mg, 0.375 mmol) was added. Stirring was continued for 30 min at room temperature. Thereafter the intermediate product **13b** was precipitated with Et_2_O, separated by decantation, and dried under reduced pressure. In order to obtain the triethoxyphosphonium salt **13** with a higher purity, the reaction should be carried out under argon atmosphere.

*1-(N-Pivaloylamino)ethyltriethoxyphosphonium tetrafluoroborate* (**13b**)*.* Colorless oil (47.6 mg, 50% yield). ^1^H-NMR (CDCl_3_) δ 7.53 (t, *J* = 8.2 Hz, 1H, NH), 4.69 (dq, *J*_1_ = 19.7, *J*_2_ = 7.0 Hz, 1H, C_α_H), 4.62–4.46 (m, 6H, 3 × OCH_2_), 1.56 (dd, *J*_1_ = 20.2, *J*_2_ = 7.3 Hz, 3H, CH_3_), 1.48 (td, *J*_1_ = 7.0, *J*_2_ = 0.9 Hz, 9H, 3 × OCH_2_CH_3_), 1.23 (s, 9H, *t*-Bu) ppm; ^13^C-NMR (CDCl_3_) δ 179.9 (C = O), 70.2 (d, *J* = 9.1 Hz, OCH_2_), 42.3 (d, *J* = 148.6 Hz, C_α_H), 38.5 (C(CH_3_)_3_), 27.1 (C(CH_3_)_3_), 15.9 (d, *J* = 6.0 Hz, OCH_2_CH_3_), 14.0 (CH_3_) ppm; ^31^P-NMR (CDCl_3_) δ 37.0 ppm; IR (ATR) 3375, 2973, 1664, 1515, 1030 cm^−1^. HRMS (TOF-ESI) calcd for C_13_H_29_NO_4_P [M]^+^ 294.1834, found 294.1829.

## 4. Conclusions

Modification of the phosphonium group by introducing electron-withdrawing substituents results in the weakening of the C_α_-P^+^ bond and makes it susceptible to cleavage. This phenomenon is the cause of the high reactivity of 1-(*N*-acylamino)alkyltriarylphosphonium salts with weakened C_α_-P^+^ bond strength. As we have demonstrated, these types of phosphonium salts react smoothly, usually at room temperature with various types of nucleophiles such as 1-morpholinocyclohexene, 1,3-dicarbonyl compounds, benzotriazole sodium salt, *p*-toluenesulfinate sodium salt, benzylamine, triarylphosphines, and other P-nucleophiles. Only in the case of 1,3-dicarbonyl compounds, it was necessary to use a strong base to generate enolate anions. Reactions with 1-morpholinocyclohexene, benzotriazole sodium salt, *p*-toluenesulfinate sodium salt, benzylamine, and triarylphosphines do not require the use of any catalysts and occur quite fast (5–60 min). Other examined P-nucleophiles also react efficiently with 1-(*N*-acylamino)alkyltriarylphosphonium salts. However, the quickly formed intermediate, in the absence of any dealkylating agent, slowly transforms into a final product. Therefore, to facilitate the reaction, we used a substoichiometric amount of the dealkylating agent in the form of methyltriphenylphosphonium iodide.

The use of 1-(*N*-acylamino)alkyltriarylphosphonium salts with weakened C_α_-P^+^ bond strength allowed us to discover interesting mechanistic aspects of the examined reactions. Detection, isolation, and characterization of 1-(*N*-acylamino)alkyltrialkoxyphosphonium salt **13b**—the reactive intermediate in the Michaelis–Arbuzov type reaction, were particularly important.

Futher studies on expanding the range of nucleophiles, which can be used in α-amidoalkylation by 1-(*N*-acylamino)alkyltriarylphosphonium salts with weakened C_α_-P^+^ bond strength, are in progress.

## Figures and Tables

**Table 1:**
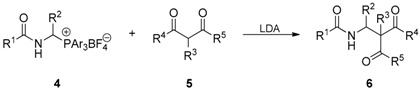
Conditions and yields for α-amidoalkylation of 1,3-dicarbonyl compounds by 1-(*N*-acylamino)alkyltriarylphosphonium salts **4**.

Entry	Phosphonium Salt 4			1,3-Dicarbonyl Compound, 5	Molar Ratio of 4:5:LDA	6	Yield, %
	R^1^	R^2^	Ar				
1	*t*-Bu	Me	*m*-C_6_H_4_Cl	diethyl malonate	1:2:1	**6a**	40
2	*t*-Bu	Me	*m*-C_6_H_4_Cl	diethyl malonate	1:8:1	**6a**	65
3	*t*-Bu	Me	*p*-C_6_H_4_CF_3_	diethyl malonate	1:8:1	**6a**	67
4	*t*-Bu	Me	*m*-C_6_H_4_Cl	ethyl acetoacetate	1:8:1	**6b**	62 ^a^
5	Bn	*i*-Bu	*m*-C_6_H_4_Cl	ethyl acetoacetate	1:8:1	**6c**	83 ^b^
6	Bn	*i*-Bu	*m*-C_6_H_4_Cl	dimethyl malonate	1:8:1	**6d**	63
7	Bn	*i*-Bu	*m*-C_6_H_4_Cl	diethyl malonate	1:8:1	**6e**	52
8	BnO	Bn	*m*-C_6_H_4_Cl	ethyl acetoacetate	1:8:1	**6f**	30 ^c^

^a^ A mixture of diastereoisomers in a molar ratio of 1.4:1. ^b^ A mixture of diastereoisomers in a molar ratio of 1.25:1. ^c^ Attempts to isolate an analytically pure sample failed.

**Table 2:**
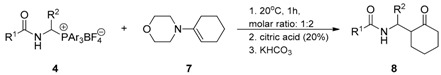
Conditions and yields for α-amidoalkylation of 1-morpholinocyclohexene by 1-(*N*-acylamino)alkyltriarylphosphonium salts **4**.

Entry	Phosphonium Salt 4			8	Yield, %
	R^1^	R^2^	Ar		
1	*t*-Bu	Me	*m*-C_6_H_4_Cl	**8a**	63 ^a^
2	Bn	*i*-Bu	*m*-C_6_H_4_Cl	**8b**	62 ^b^
3	BnO	*i*-Bu	*m*-C_6_H_4_Cl	**8c**	40 ^c^
4	BnO	Bn	*m*-C_6_H_4_Cl	**8d**	33 ^d^
5	BnO	CH_2_O*t*-Bu	*p*-C_6_H_4_CF_3_	**8e**	31 ^c^

^a^ A mixture of diastereoisomers in a molar ratio of 3.5:1. ^b^ A mixture of diastereoisomers in a molar ratio of 3.2:1. ^c^ Only one diastereoisomer was detected and isolated. ^d^ A mixture of diastereoisomers in a molar ratio of 13.4:1.

**Table 3:**
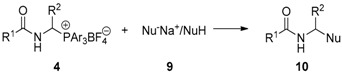
Conditions and yields for α-amidoalkylation of selected heteronucleophiles by 1-(*N*-acylamino)alkyltriarylphosphonium salts **4**.

Entry	Phosphonium Salt 4			Nu^−^Na^+^/NuH	Temp., °C	Time, Min.	Molar Ratio of 4:9	10	Yield, %
	R^1^	R^2^	Ar						
1	*t*-Bu	Me	*p*-C_6_H_4_CF_3_	Bt**^−^**Na^+^	20	15	1:1	**10a**	99
2	BnO	Bn	*m*-C_6_H_4_Cl	Bt**^−^**Na^+^	20	15	1:1	**10b**	70
3	*t*-Bu	Me	*p*-C_6_H_4_CF_3_	TolSO_2_**^−^**Na^+^	20	15	1:1	**10c**	88
4	*t*-Bu	Me	*m*-C_6_H_4_Cl	Bn-NH_2_	20	5	1:4	**10d**	91
5	Bn	*i*-Bu	*p*-C_6_H_4_CF_3_	Bn-NH_2_	20	5	1:4	**10e**	55

**Table 4:**
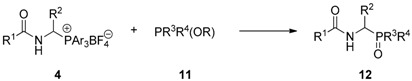
Conditions and yields for α-amidoalkylation of P-nucleophiles by 1-(*N*-acylamino)alkyltriarylphosphonium salts **4**.

Entry	Phosphonium Salt 4			P-Nu 11			Temp., °C	Time, h	Molar Ratio of 4:11	12	Yield, %
	R^1^	R^2^	Ar	R	R^3^	R^4^					
1	*t*-Bu	Me	*m*-C_6_H_4_Cl	Me	OMe	OMe	20	3	1:1.5	**12a**	47
2	*t*-Bu	Me	*m*-C_6_H_4_Cl	Me	OMe	OMe	20	3	1:1.5 ^a^	**12a**	85
3	Bn	*i*-Bu	*m*-C_6_H_4_Cl	Me	OMe	OMe	20	3	1:1.5 ^a^	**12b**	77
4	BnO	CH_2_O*t*-Bu	*p*-C_6_H_4_CF_3_	Me	OMe	OMe	20	3	1:1.5 ^a^	**12c**	61
5	*t*-Bu	Me	*m*-C_6_H_4_Cl	Me	Ph	OMe	60	2	1:1.5 ^a^	**12d**	69
6	BnO	*i*-Bu	*p*-C_6_H_4_CF_3_	Me	Ph	Ph	20	3	1:1.5 ^a^	**12e**	83

^a^ Substoichiometric amounts of methyltriphenylphosphonium iodide as a dealkylating agent (molar ratio of 1:0.25) was used.

## Supplementary Materials

The following are available online at http://www.mdpi.com/1420-3049/23/10/2453/s1, Supporting information includes a summary table (comparison of conditions and yields for reactions of 1-(*N*-acylamino)alkyltriphenylphosphonium salts (former studies) and 1-(*N*-acylamino)alkyltriarylphosphonium salts (the current work) with selected nucleophiles), ^1^H, ^13^C, and ^31^P NMR spectra of all new compounds **6**, **8**, **10**, **12**, **13**. Supplementary data associated with this article can be found in the online version.
