# Supplementary material for: 1-(N-Acylamino)alkyltriarylphosphonium Salts with Weakened Cα-P+ Bond Strength—Synthetic Applications
Source: Molecules. 2018 Sep 25;23(10):2453. doi: 10.3390/molecules23102453 (PMC6222910; doi:10.3390/molecules23102453)
Supplement: Supplementary file 1 [file molecules-23-02453-s001.pdf]

molecules
MDPI

Article

1-(*N*-Acylamino)alkyltriarylphosphonium salts with modulated C$_\alpha$-P$^+$ bond strength—synthetic application

Jakub Adamek[1,2,*], Anna Węgrzyk[1,2], Justyna Kończewicz[1,2], Krzysztof Walczak[1] and Karol Erfurt[3]

[1] Department of Organic Chemistry, Bioorganic Chemistry and Biotechnology, Silesian University of Technology, B. Krzywoustego 4, 44-100 Gliwice, Poland; jakub.adamek@polsl.pl, anna.wegrzyk@polsl.pl, j.konczewicz@gmail.com, krzysztof.walczak@polsl.pl
[2] Biotechnology Center of Silesian University of Technology, B. Krzywoustego 8, 44-100 Gliwice, Poland
[3] Department of Chemical Organic Technology and Petrochemistry, Silesian University of Technology, B. Krzywoustego 4, 44-100 Gliwice, Poland; karol.erfurt@polsl.pl
* Correspondence: jakub.adamek@polsl.pl; Tel.: +48 032-237-1080; fax: +48 032-237-2094

Supporting information

Table of contents

1. Comparison of conditions and yields for reactions of 1-(*N*-acylamino)alkyltriphenylphosphonium salts (former studies) and 1-(*N*-acylamino)alkyltriarylphosphonium salts (the current work) with selected nucleophiles – a summary table……………………………………………………………......S2
2. ^{1}H NMR, ^{13}C NMR and ^{31}P NMR of all new compounds **6, 8, 10, 12** and **13**...……..........S3-S30
3. IR spectra for selected compounds…………………….…………….....….…….……….......S31-S37
4. Examples of the measurements of the changes in concentrations for the reaction of 1-(*N*-pivaloylamino)ethyltris(3-chlorophenyl)phosphonium tetrafluoroborate **4c** with trimethylphosphite at 26°C...S38-S41

Table S1. Comparison of conditions and yields for reactions of 1-(*N*-acylamino)alkyltriphenylphosphonium salts (former studies) and 1-(*N*-acylamino)alkyltriarylphosphonium salts (the current work) with selected nucleophiles

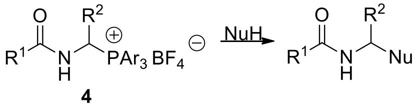

Ar = Ph, *m*-C_6H_4Cl, *p*-$C_6H_4CF_3$

Entry	Phosphonium salt 4				Nucleophile	Solvent	Catalyst	Temp., °C	MW	Time, min	Product	Yield, %	References[a]
	R¹	R²	Ar	X									
1	*t*-Bu	Me	*m*-C_6H_4Cl	BF_4	diethyl malonate	THF	LDA	20	-	15	**6a**	65	this work
2	*t*-Bu	Me	*p*-$C_6H_4CF_3$	BF_4	diethyl malonate	THF	LDA	20	-	15	**6a**	67	this work
3	*t*-Bu	Me	Ph	BF_4	diethyl malonate	THF	LDA	20	-	15	**6a**	21	this work
4	*t*-Bu	Me	Ph	I	diethyl malonate	CH_3CN	DBU	60	10-12 W[b]	90	**6a**	61	[8]
5[c]	*t*-Bu	Me	*m*-C_6H_4Cl	BF_4	1-morpholinocyclohexene	CH_3CN		20	-	60	**8a**	63	this work
6[c]	Ph	Me	Ph	I	1-morpholinocyclohexene	CH_3CN	(*i*-Pr)$_2$EtN	60	8W[b]	60	**8f**	76	[8]
7	*t*-Bu	Me	*p*-$C_6H_4CF_3$	BF_4	Bt^-Na^+	$CHCl_3$	-	20	-	15	**10a**	99	this work
8	*t*-Bu	Me	Ph	BF_4	Bt^-Na^+	$CHCl_3$	-	20	-	120	**10a**	90	[6]
9	BnO	Bn	*m*-C_6H_4Cl	BF_4	Bt^-Na^+	$CHCl_3$	-	20	-	15	**10b**	70	this work
10	BnO	Bn	Ph	BF_4	Bt^-Na^+	$CHCl_3$	-	20	-	120	**10b**	74	[6]
11	*t*-Bu	Me	*p*-$C_6H_4CF_3$	BF_4	$TolSO_2^-Na^+$	$CHCl_3$	-	20	-	15	**10c**	88	this work
12	*t*-Bu	Me	Ph	BF_4	$TolSO_2^-Na^+$	$CHCl_3$	-	20	-	120	**10c**	90	[5]
13	*t*-Bu	Me	*m*-C_6H_4Cl	BF_4	$P(OMe)_3$	$CHCl_3$	-	20	-	180	**12a**	85	this work
14	*t*-Bu	Me	Ph	BF_4	$P(OMe)_3$	CH_2Cl_2	(*i*-Pr)$_2$EtN	60	-	120	**12a**	89	[7]
15	Bn	*i*-Bu	*m*-C_6H_4Cl	BF_4	$P(OMe)_3$	$CHCl_3$	-	20	-	180	**12b**	77	this work
16	Bn	*i*-Bu	Ph	BF_4	$P(OMe)_3$	CH_2Cl_2	(*i*-Pr)$_2$EtN	60	-	240	**12b**	83	[4]
17	BnO	*i*-Bu	*p*-$C_6H_4CF_3$	BF_4	Ph_2POMe	$CHCl_3$	-	20	-	180	**12e**	**83**	this work
18	BnO	*i*-Bu	Ph	BF_4	Ph_2POMe	CH_2Cl_2	(*i*-Pr)$_2$EtN	60	-	120	**12e**	56	[8]

[a]See *References* in the main text of the publication. [b]The average microwave power that provides the desired reaction temperature. [c]Substrates differ slightly in structure. More accurate data are not available.

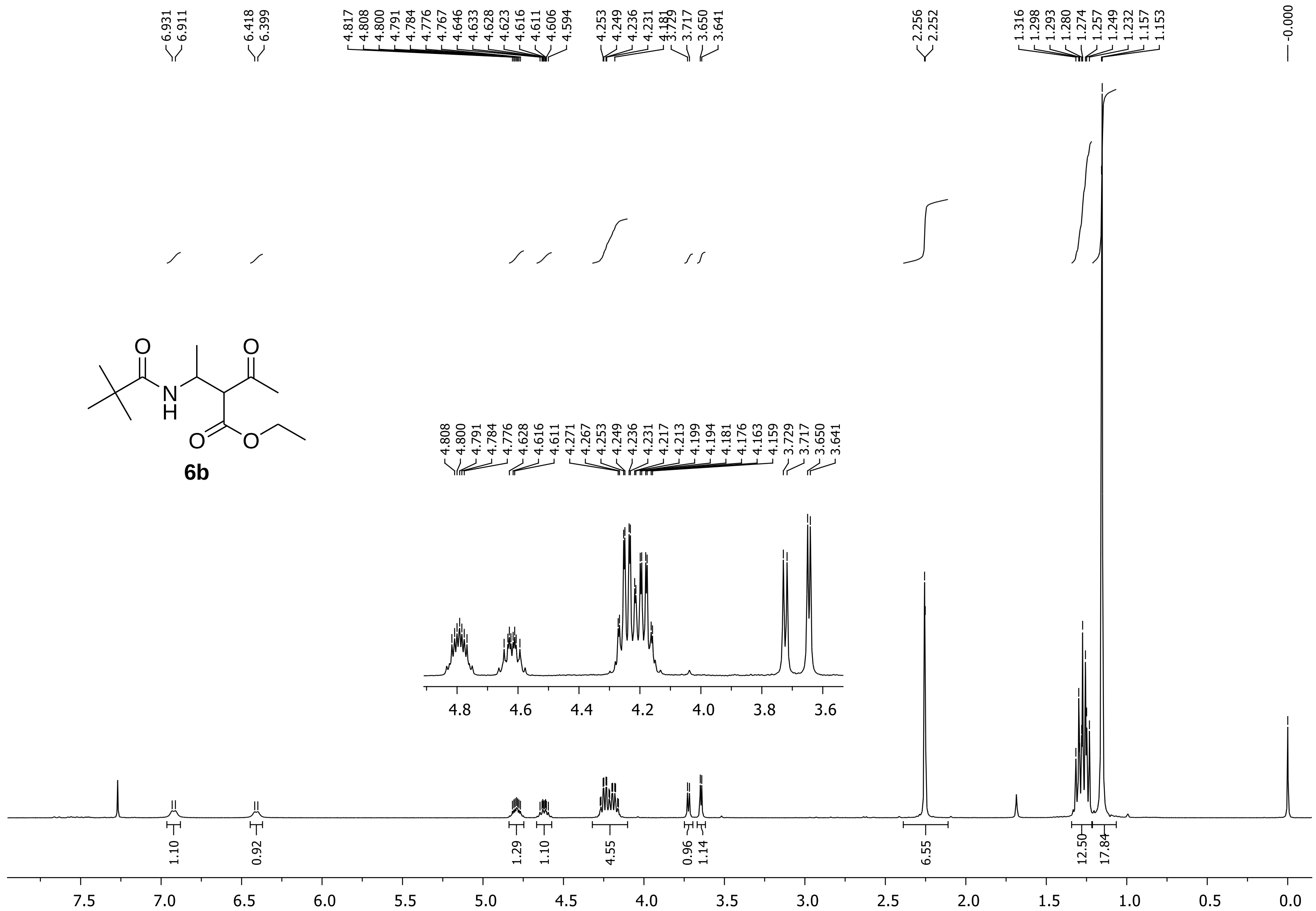

^{1}H NMR spectrum of ethyl 2-acetyl-3-(pivaloylamino)butanoate (**6b**) – the mixture of two diastereoisomers; 400 MHz/$CDCl_3$/TMS; δ (ppm).

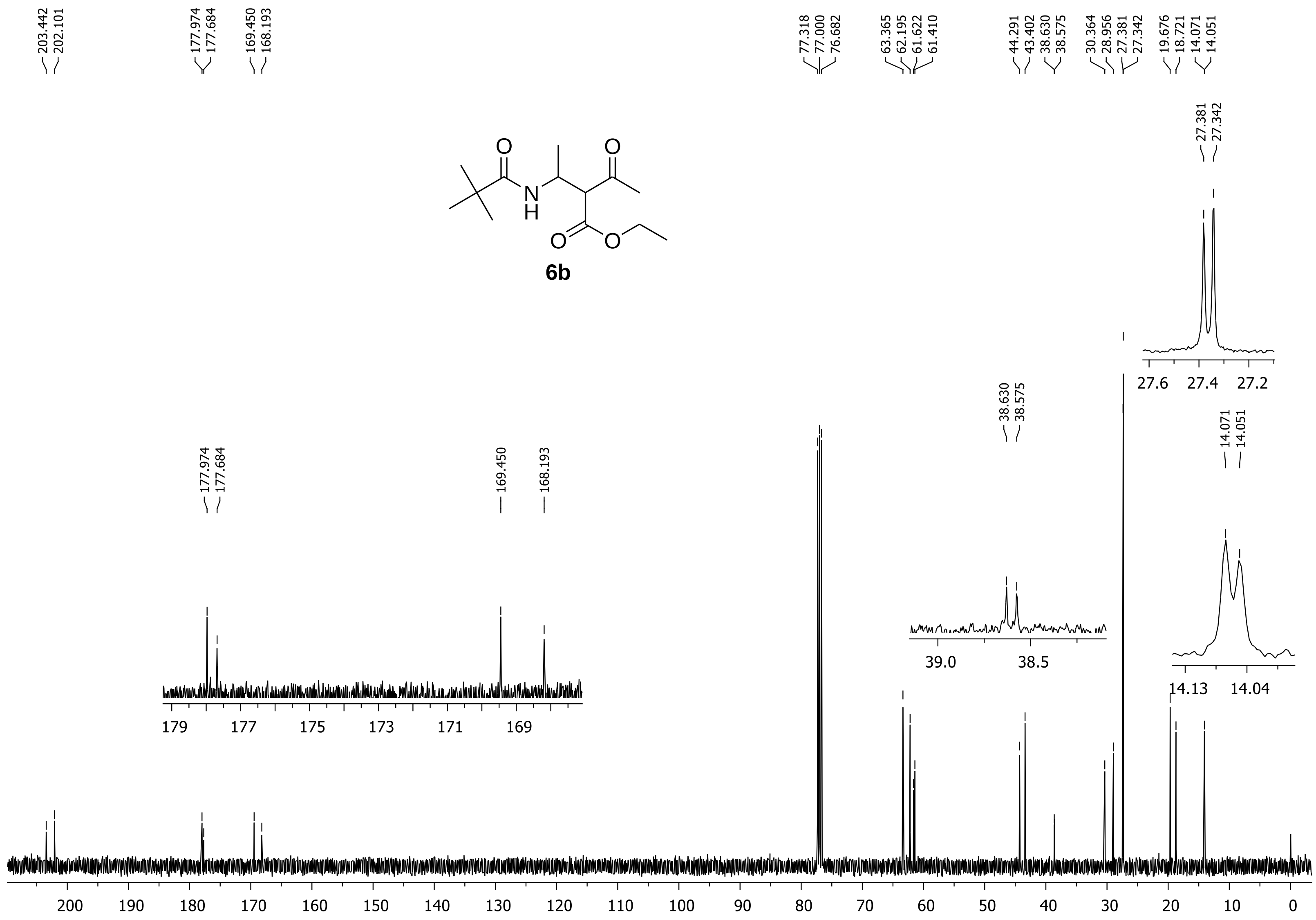

^{13}C NMR spectrum of ethyl 2-acetyl-3-(pivaloylamino)butanoate (**6b**) - the mixture of two diastereoisomers; 100 MHz/$CDCl_3$/TMS; δ (ppm).

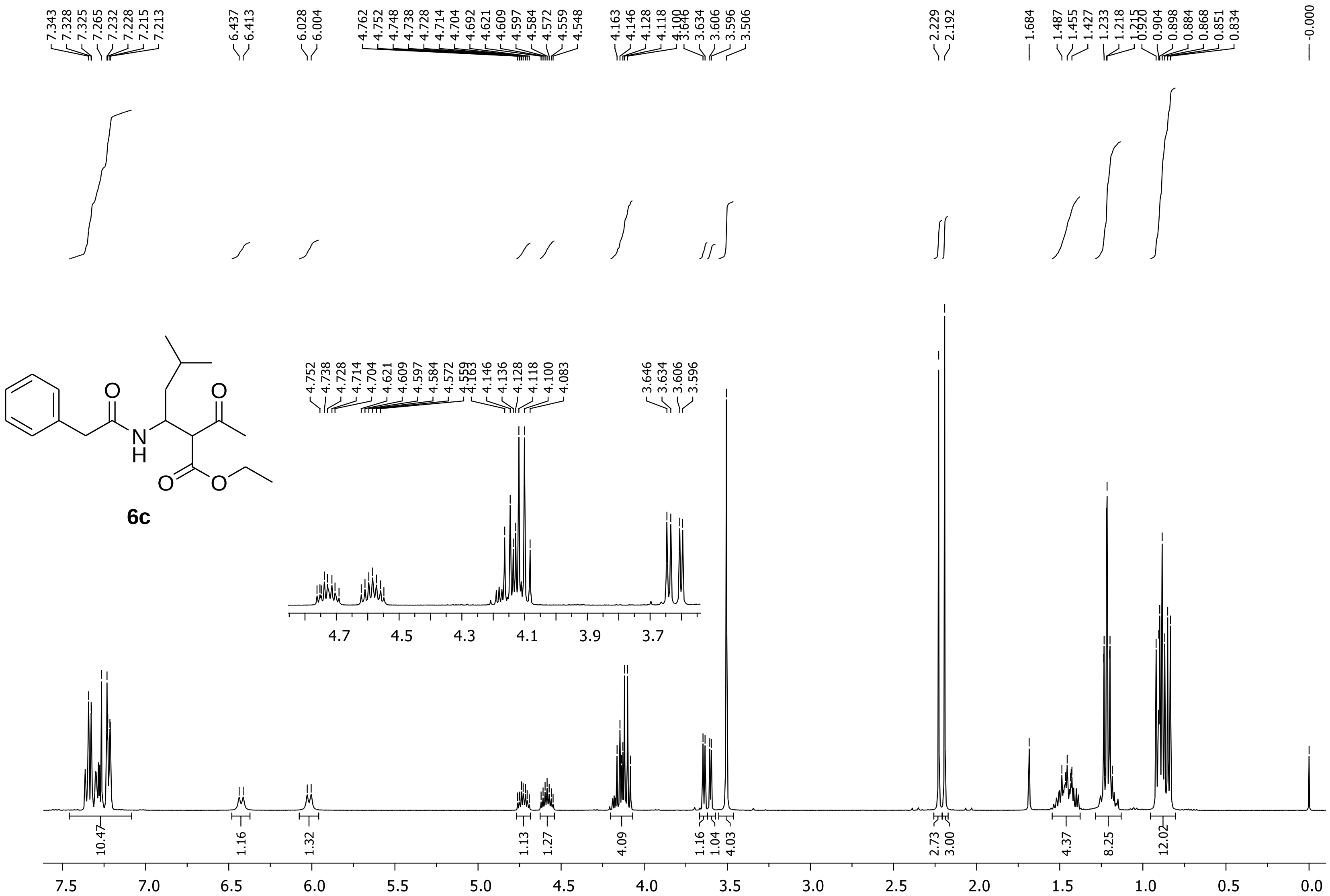

^{1}H NMR spectrum of ethyl 2-acetyl-5-methyl-3-(phenylacetylamino)hexanoate (**6c**) - the mixture of two diastereoisomers; 400 MHz/$CDCl_3$/TMS; δ (ppm).

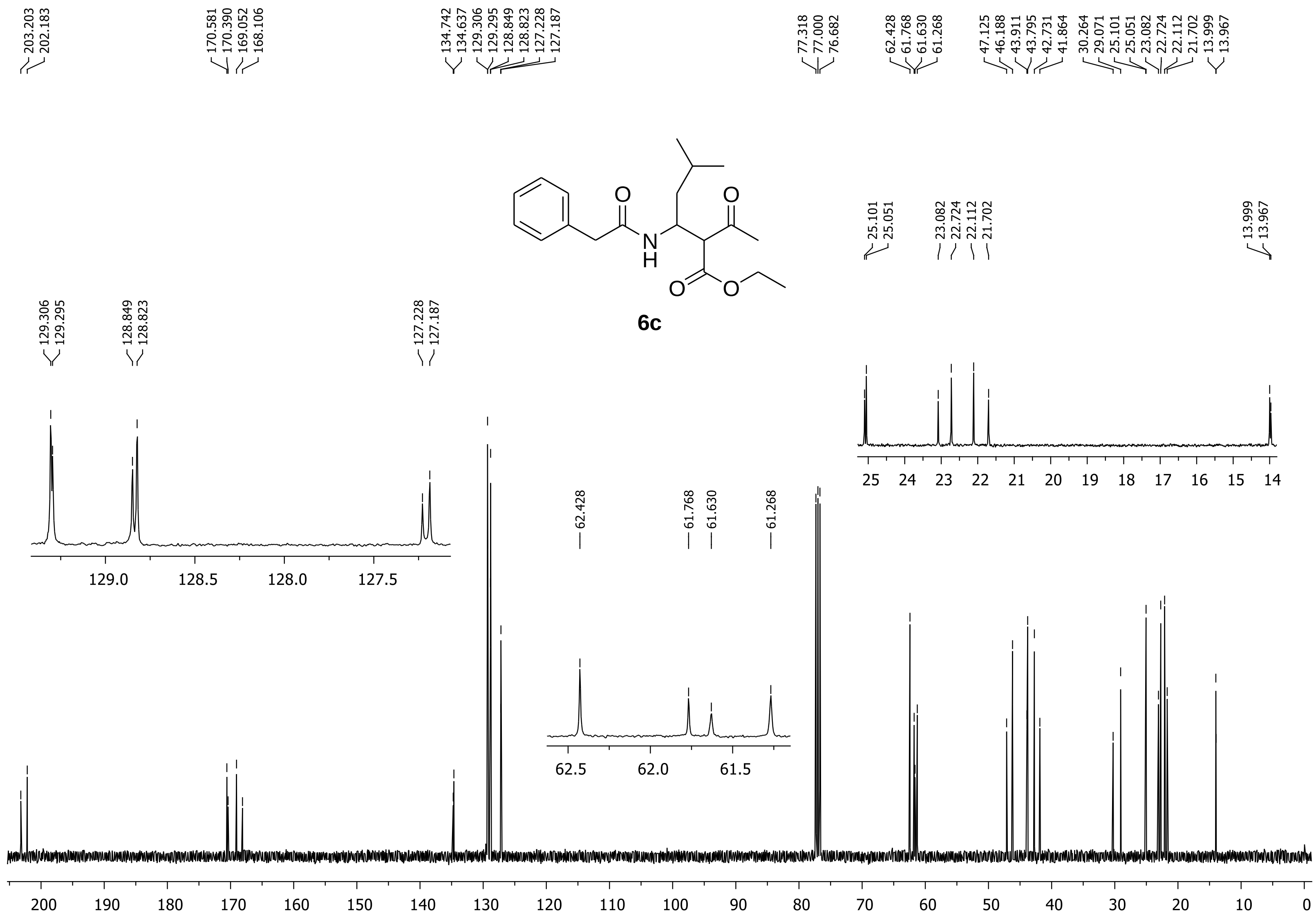

^{13}C NMR spectrum of ethyl 2-acetyl-5-methyl-3-(phenylacetylamino)hexanoate (**6c**) - the mixture of two diastereoisomers; 100 MHz/$CDCl_3$/TMS; δ (ppm).

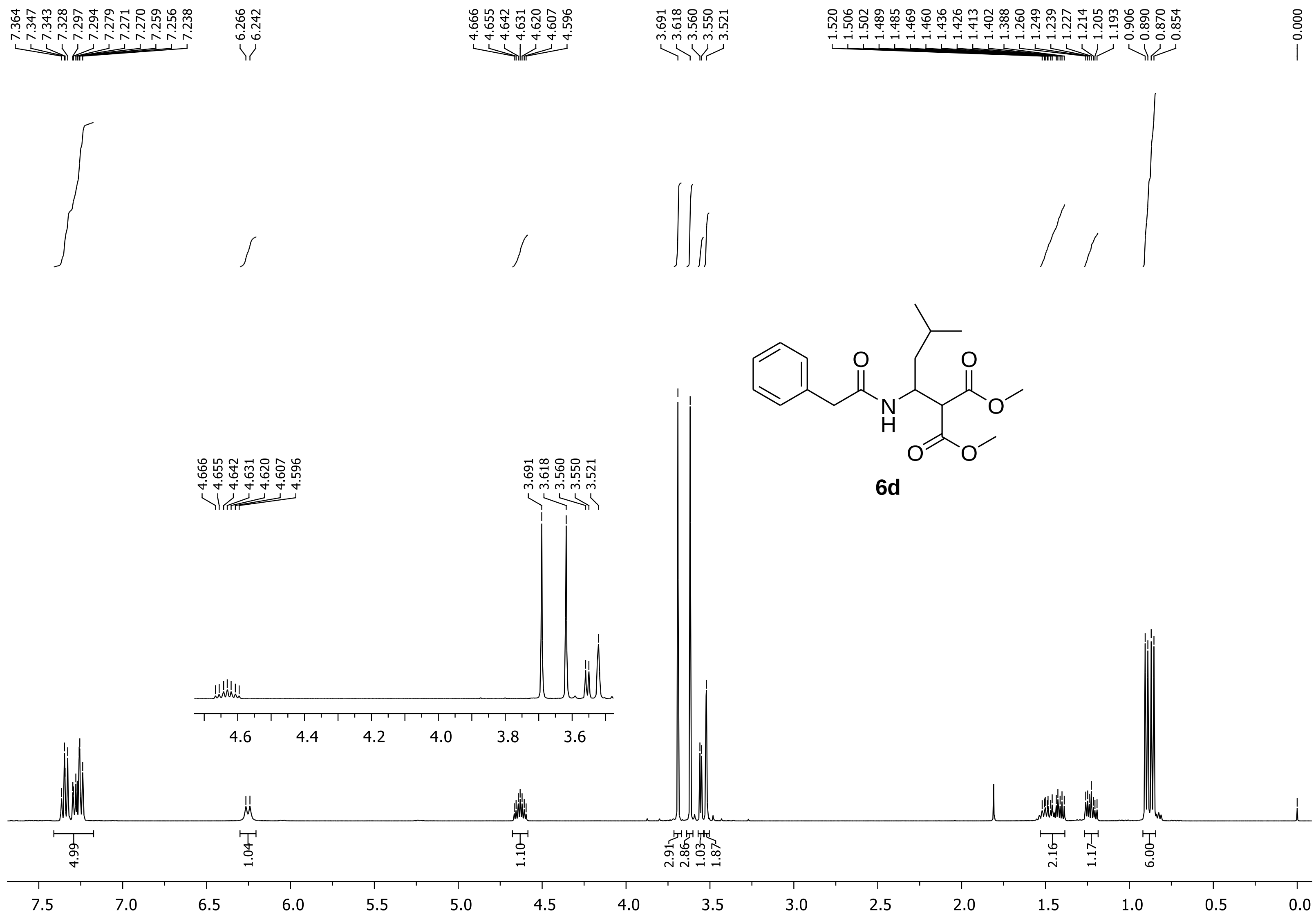

^{1}H NMR spectrum of dimethyl 3-methyl-1-(phenylacetylamino)butylpropanedioate (**6d**); 400 MHz/$CDCl_3$/TMS; δ (ppm).

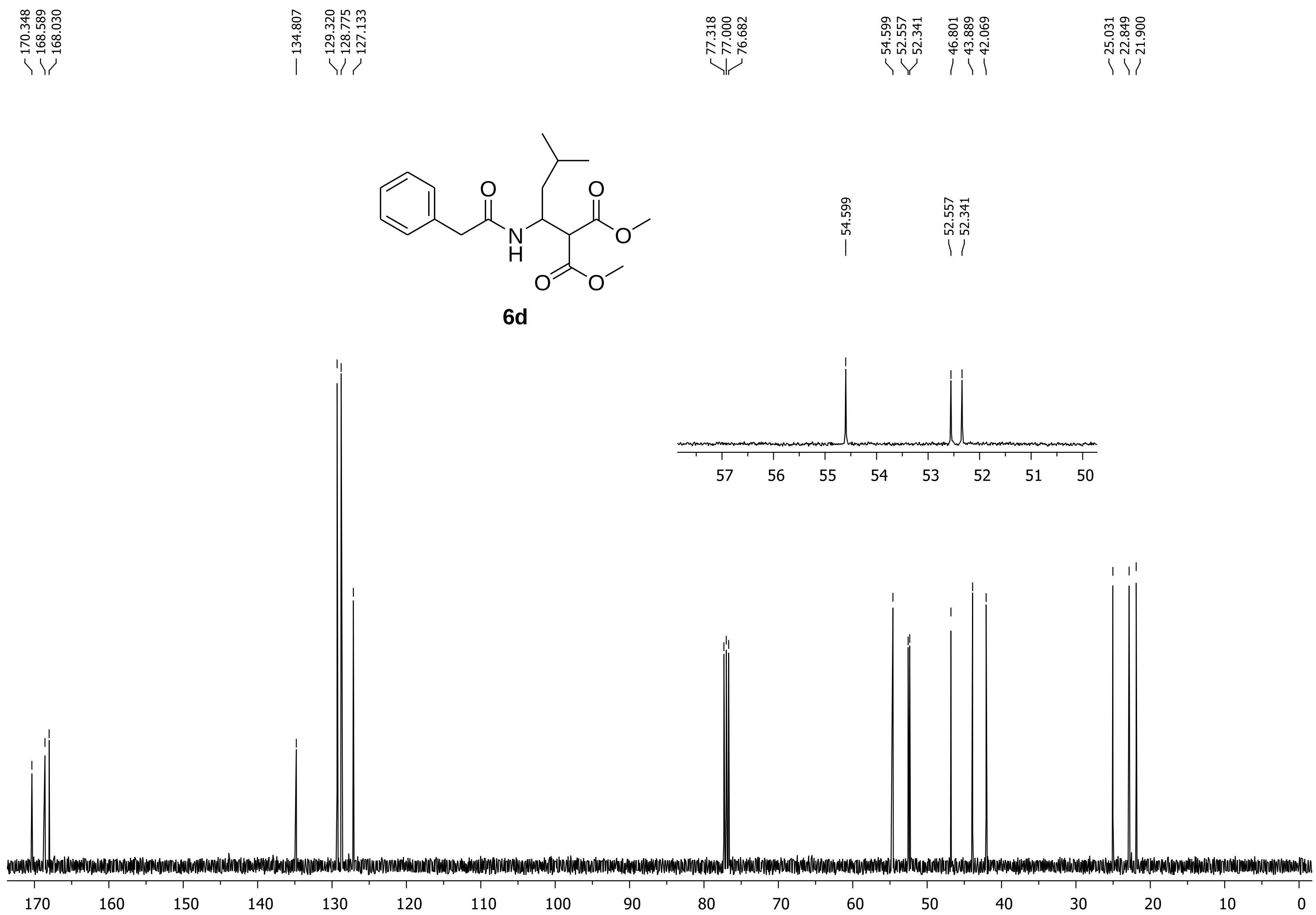

^{13}C NMR spectrum of dimethyl 3-methyl-1-(phenylacetylamino)butylpropanedioate (**6d**); 100 MHz/$CDCl_3$/TMS; δ (ppm).

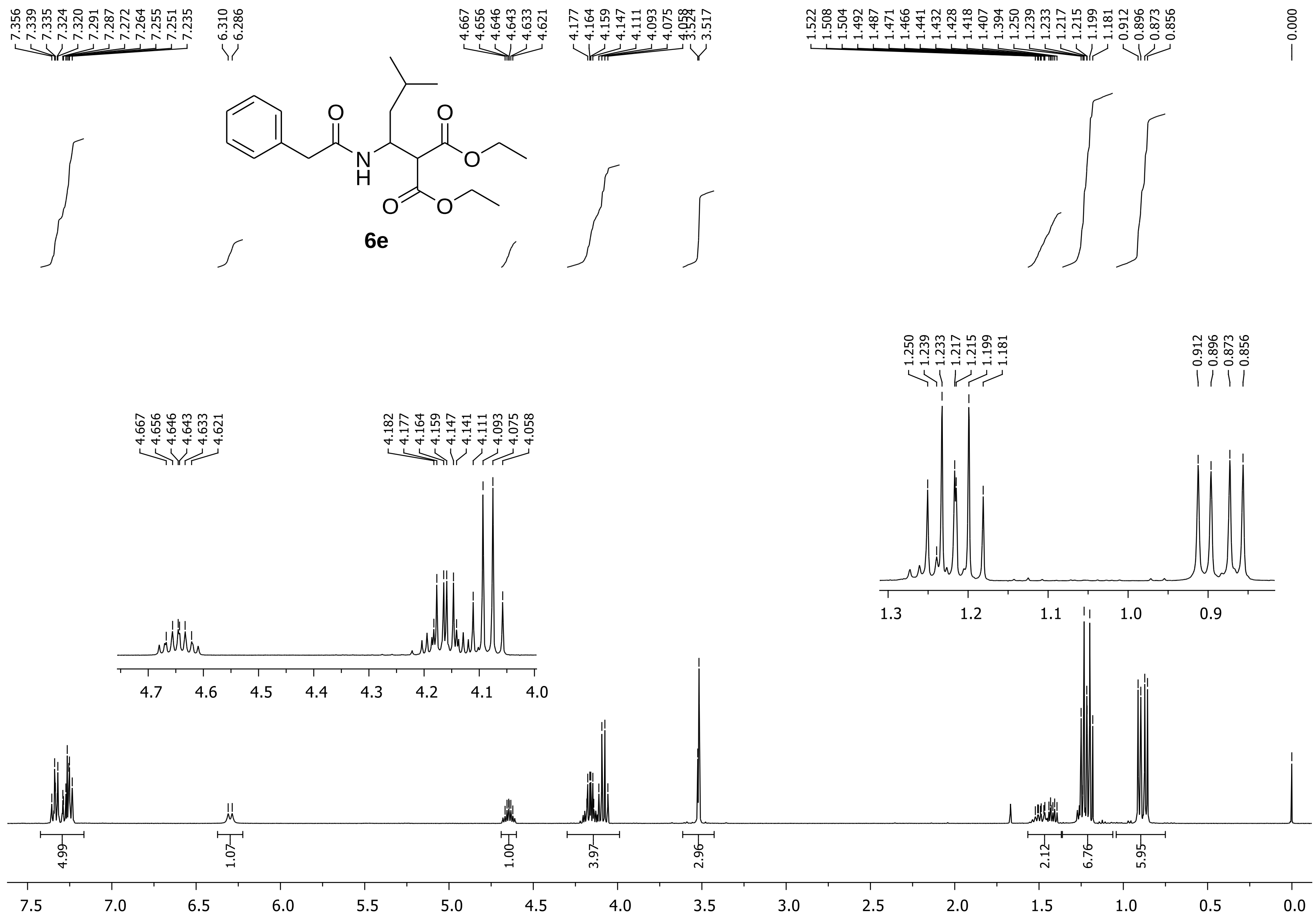

^{1}H NMR spectrum of diethyl 3-methyl-1-(phenylacetylamino)butylpropanedioate (**6e**); 400 MHz/$CDCl_3$/TMS; δ (ppm).

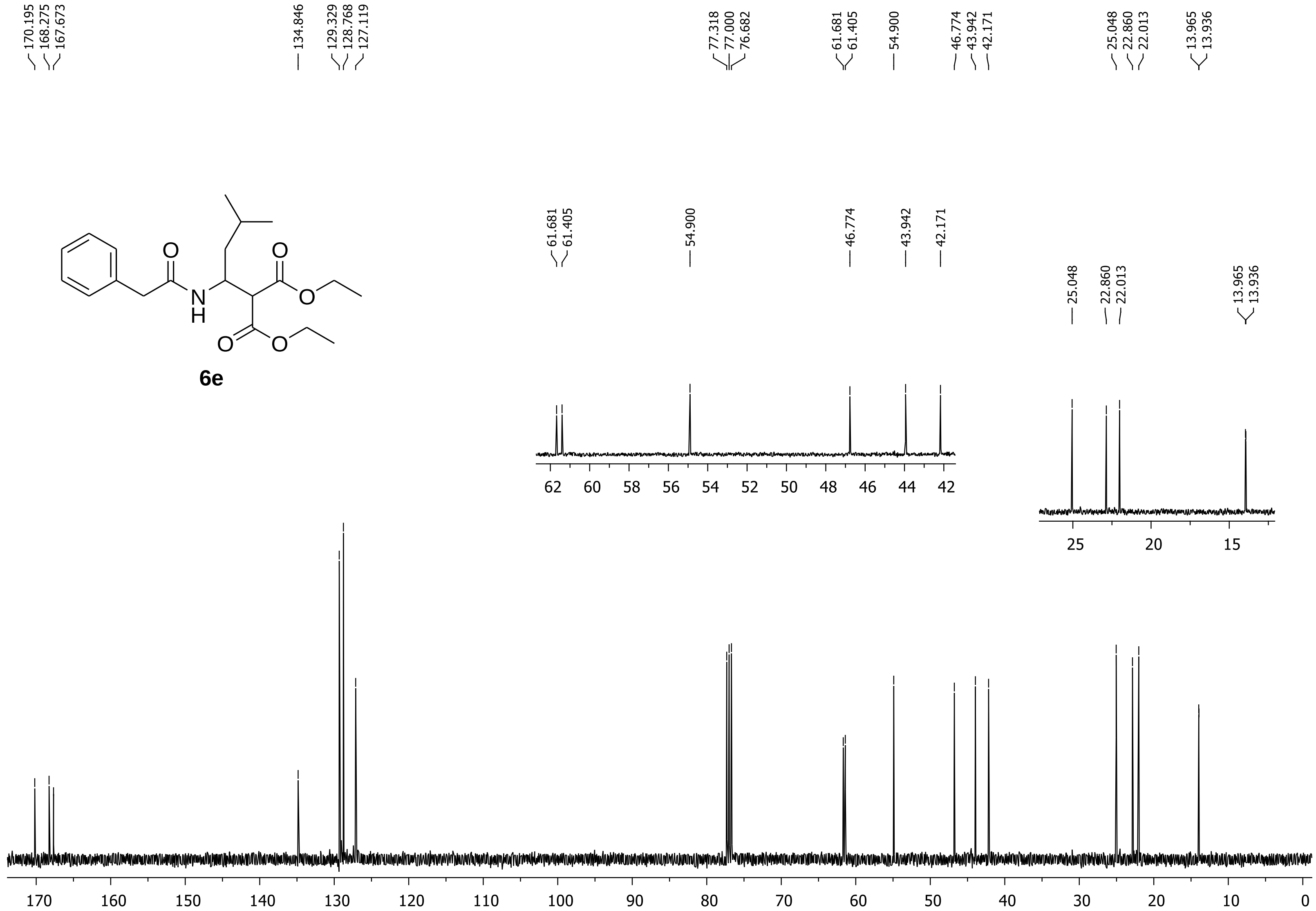

^{13}C NMR spectrum of diethyl 3-methyl-1-(phenylacetylamino)butylpropanedioate (**6e**); 100 MHz/$CDCl_3$/TMS; δ (ppm).

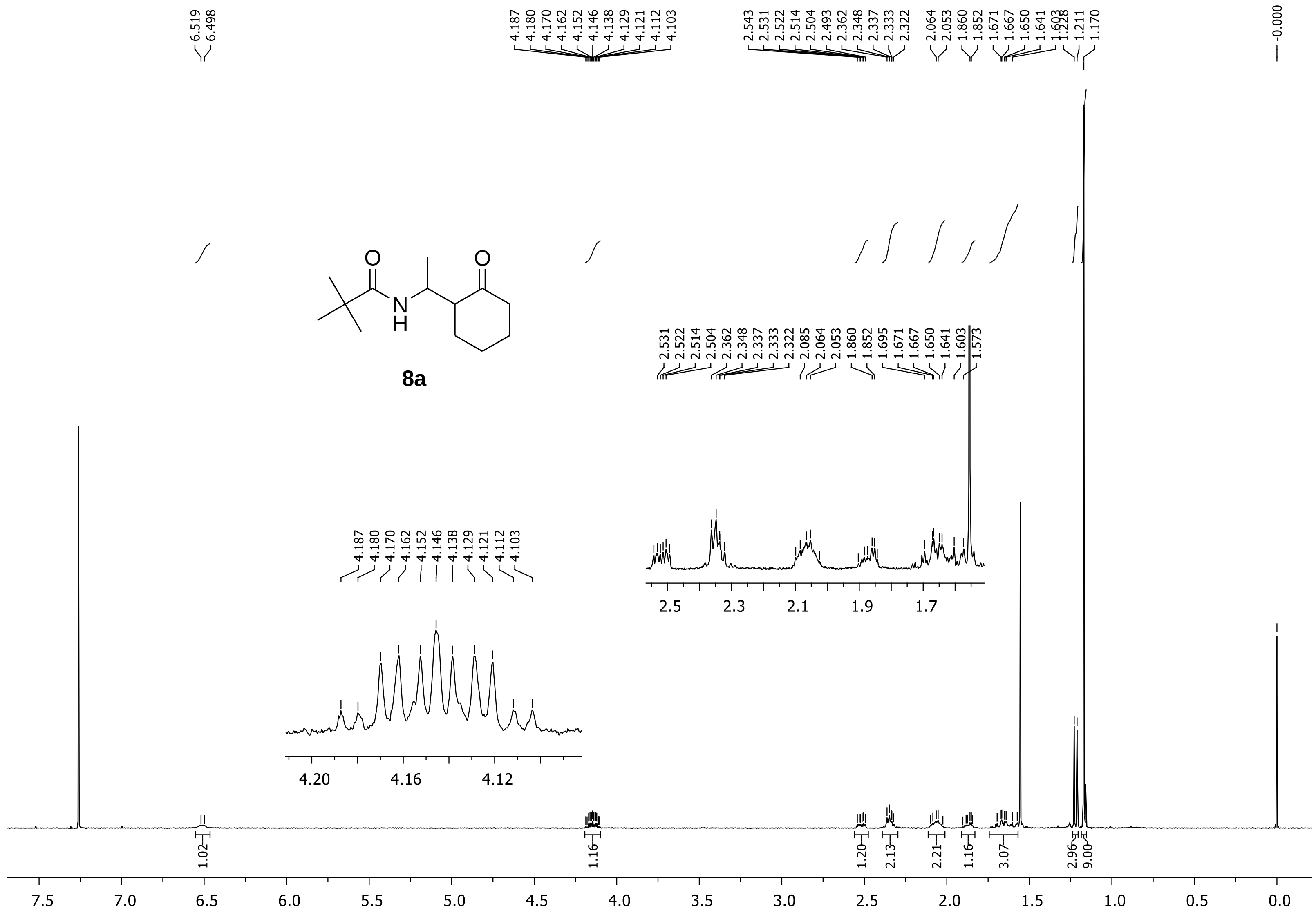

^{1}H NMR spectrum of *N*-[1-(2-oxocyclohexyl)ethyl]pivalamide (**8a**) – the major disatereoisomer; 400 MHz/$CDCl_3$/TMS; δ (ppm).

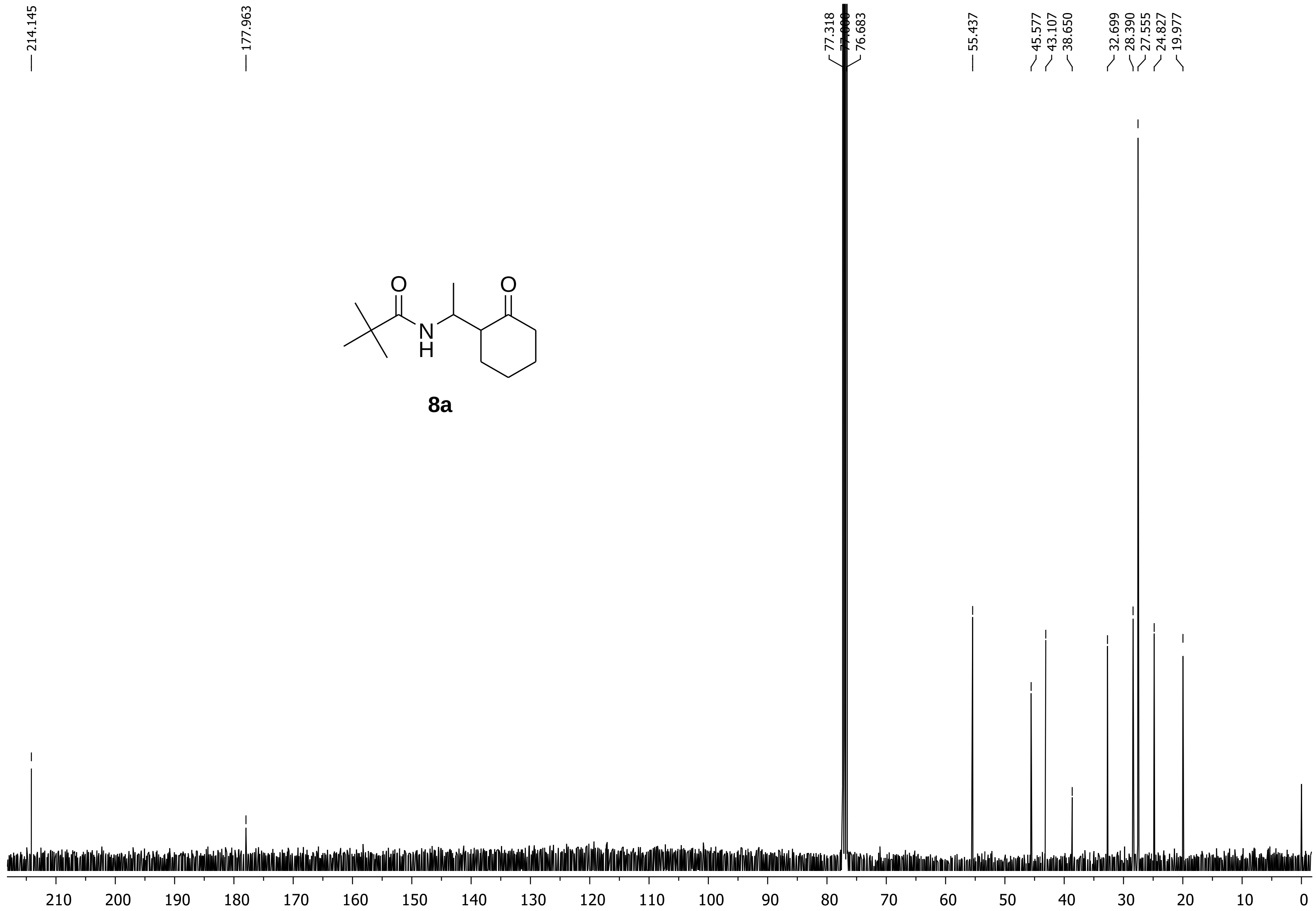

^{13}C NMR spectrum of *N*-[1-(2-oxocyclohexyl)ethyl]pivalamide (**8a**) - the major disatereoisomer; 100 MHz/$CDCl_3$/TMS; δ (ppm).

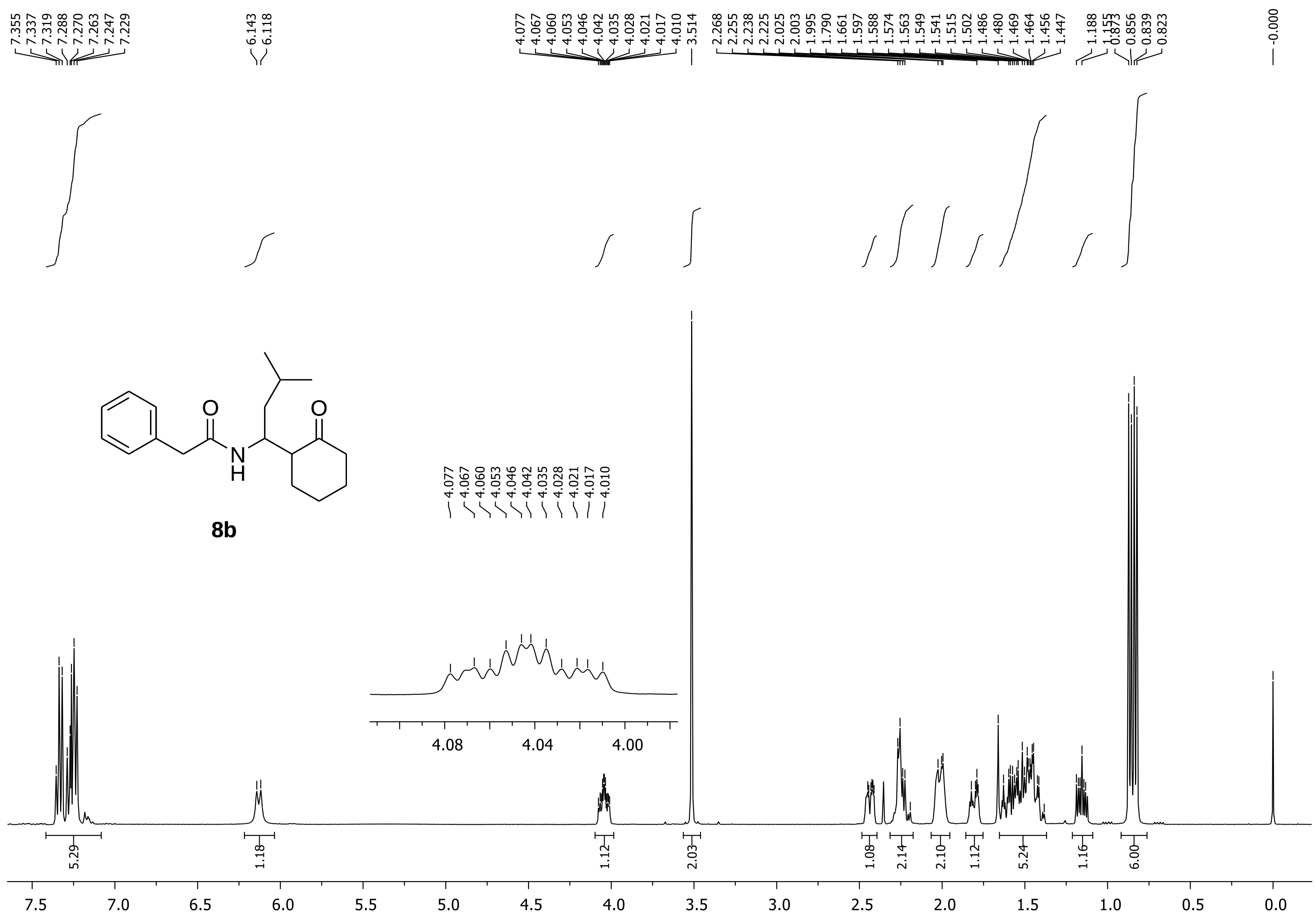

^{1}H NMR spectrum of *N*-[1-(2-oxocyclohexyl)-3-methylbutyl]phenylacetamide (**8b**) – the major disatereoisomer; 400 MHz/$CDCl_3$/TMS; δ (ppm).

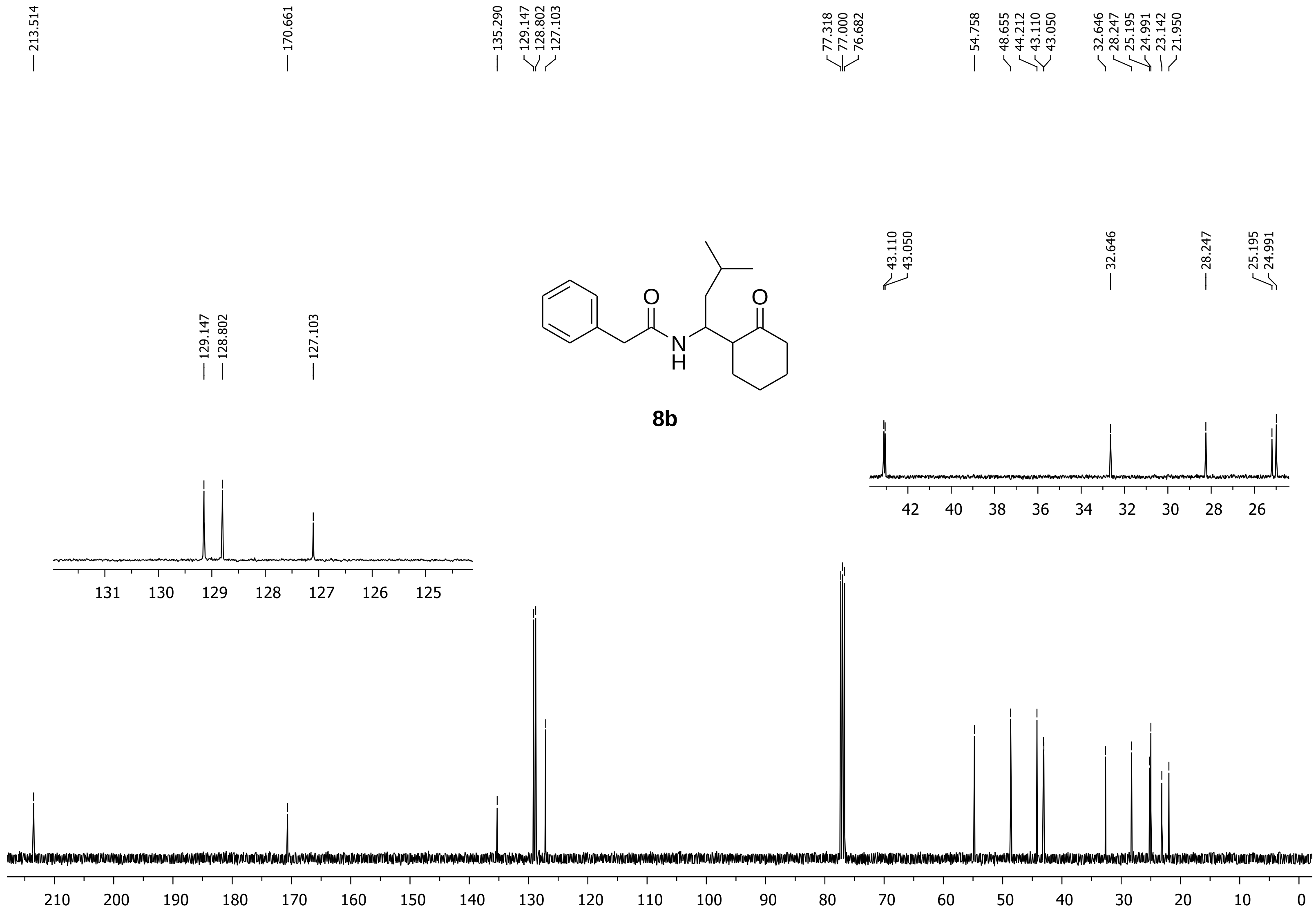

^{13}C NMR spectrum of *N*-[1-(2-oxocyclohexyl)-3-methylbutyl]phenylacetamide (**8b**) – the major disatereoisomer; 100 MHz/$CDCl_3$/TMS; δ (ppm).

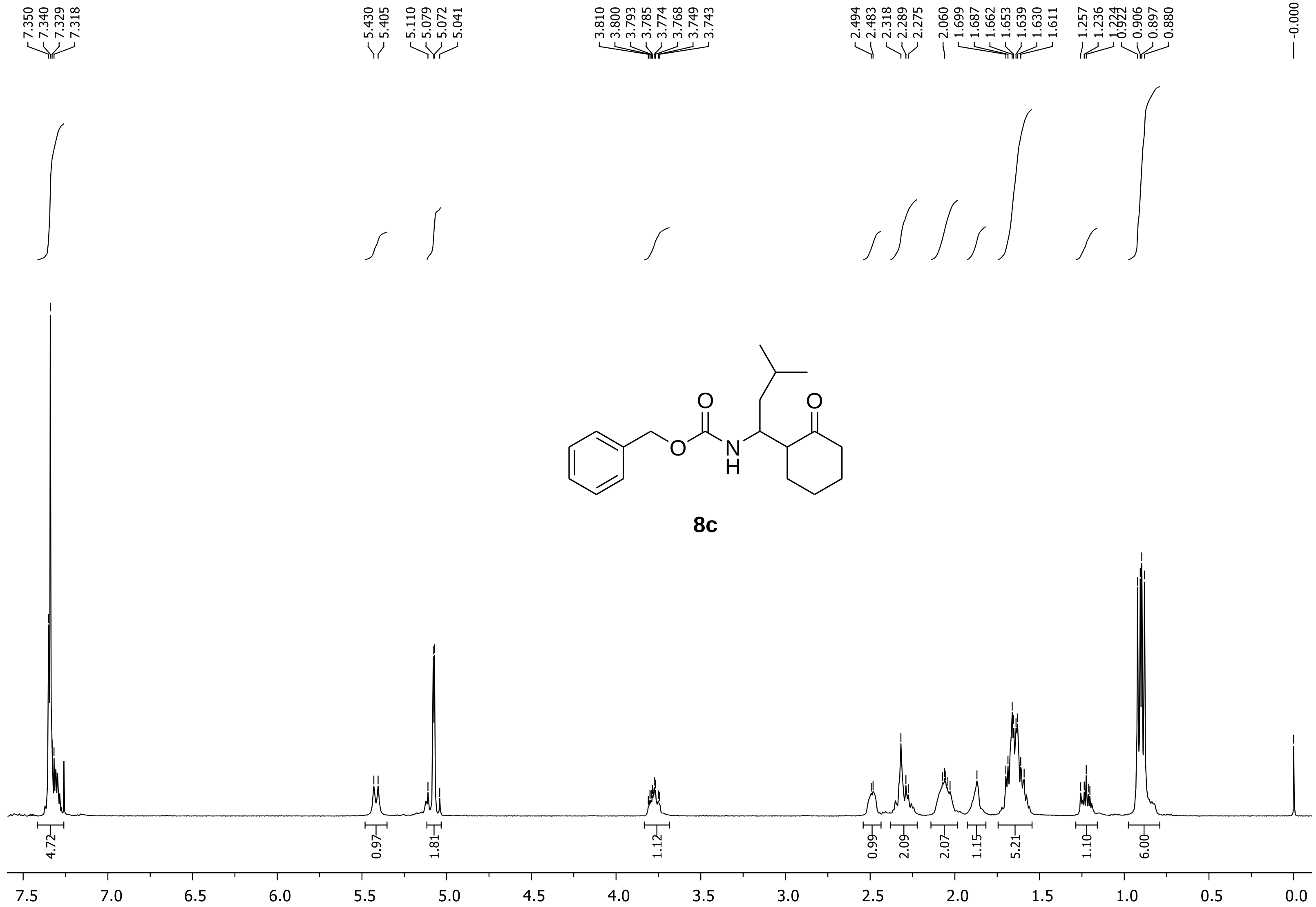

^{1}H NMR spectrum of benzyl *N*-[1-(2-oxocyclohexyl)-3-methylbutyl]carbamate (**8c**); 400 MHz/$CDCl_3$/TMS; δ (ppm).

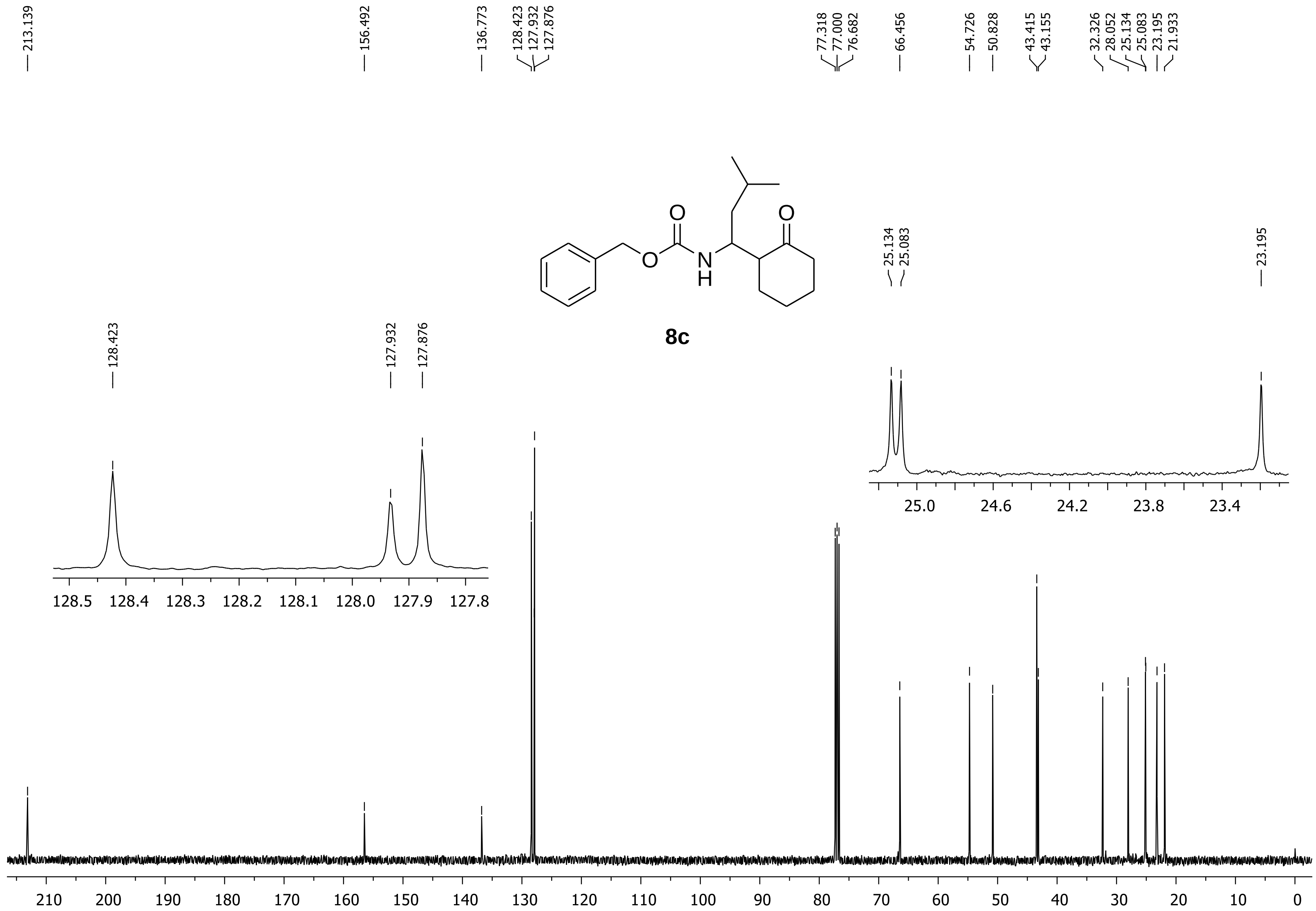

^{13}C NMR spectrum of benzyl *N*-[1-(2-oxocyclohexyl)-3-methylbutyl]carbamate (**8c**); 100 MHz/$CDCl_3$/TMS; δ (ppm).

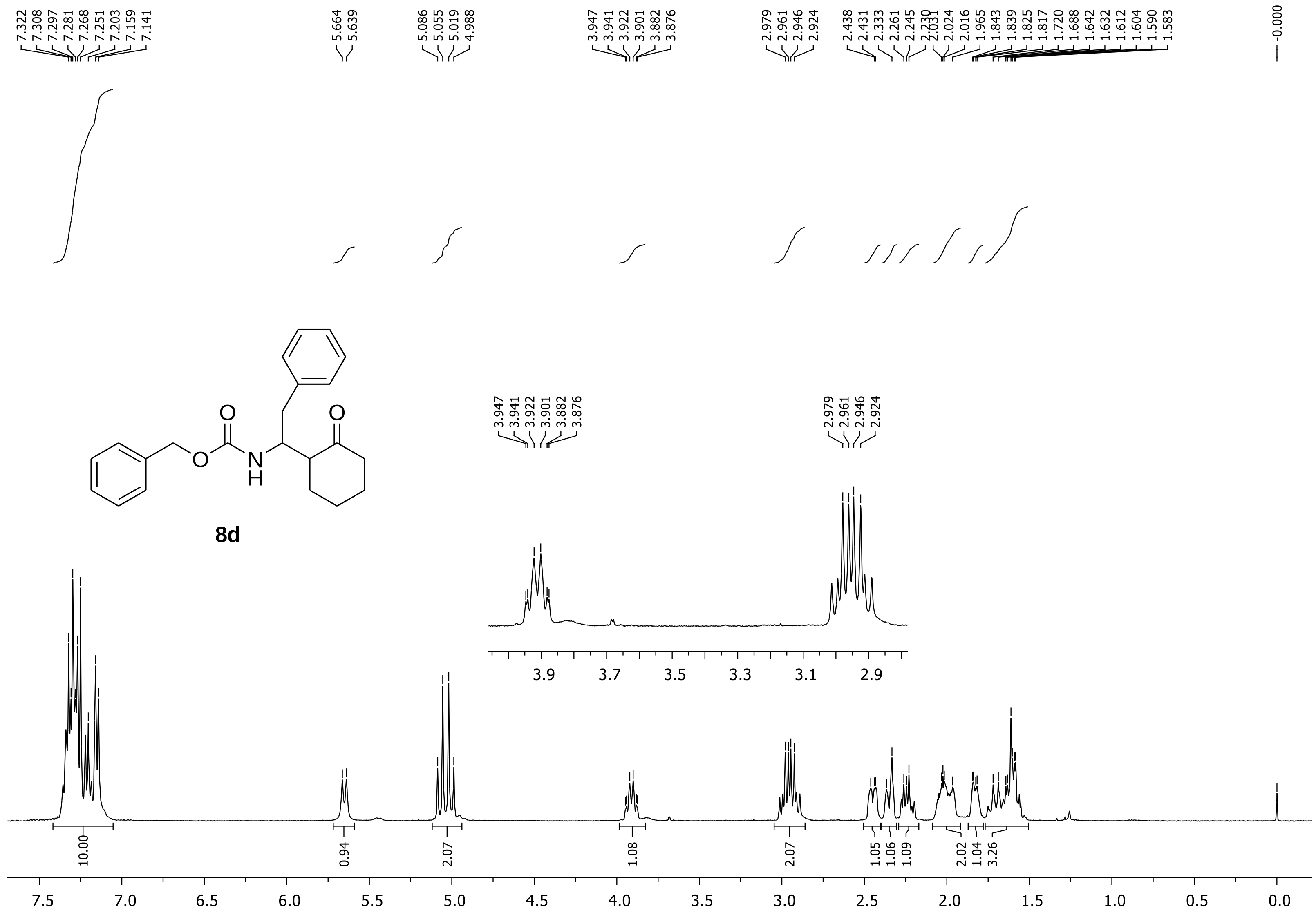

^{1}H NMR spectrum of benzyl *N*-[1-(2-oxocyclohexyl)-2-phenylethyl]carbamate (**8d**) – the major disatereoisomer; 400 MHz/$CDCl_3$/TMS; δ (ppm).

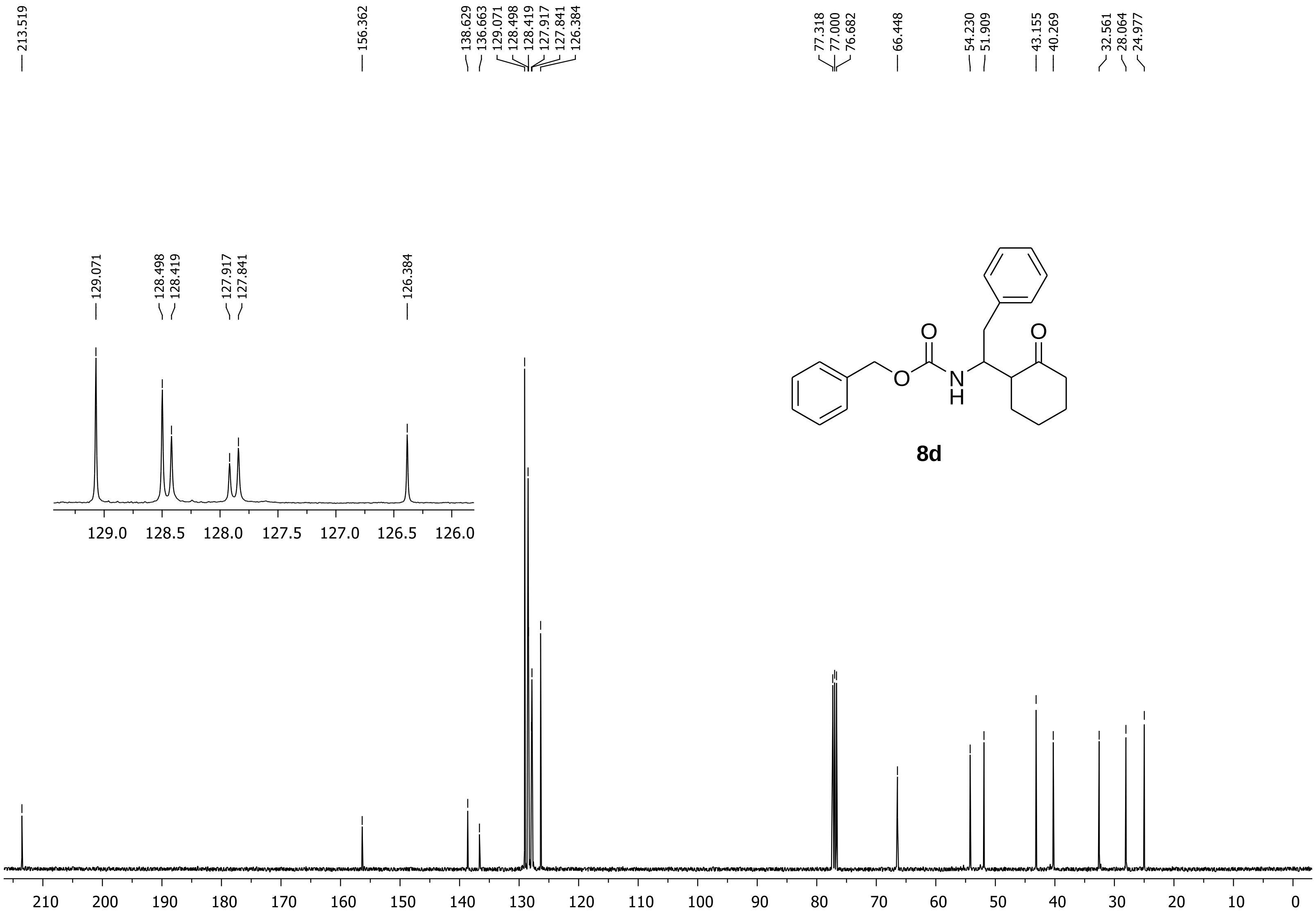

8d

^{13}C NMR spectrum of benzyl *N*-[1-(2-oxocyclohexyl)-2-phenylethyl]carbamate (**8d**) – the major disatereoisomer; 100 MHz/$CDCl_3$/TMS; δ (ppm).

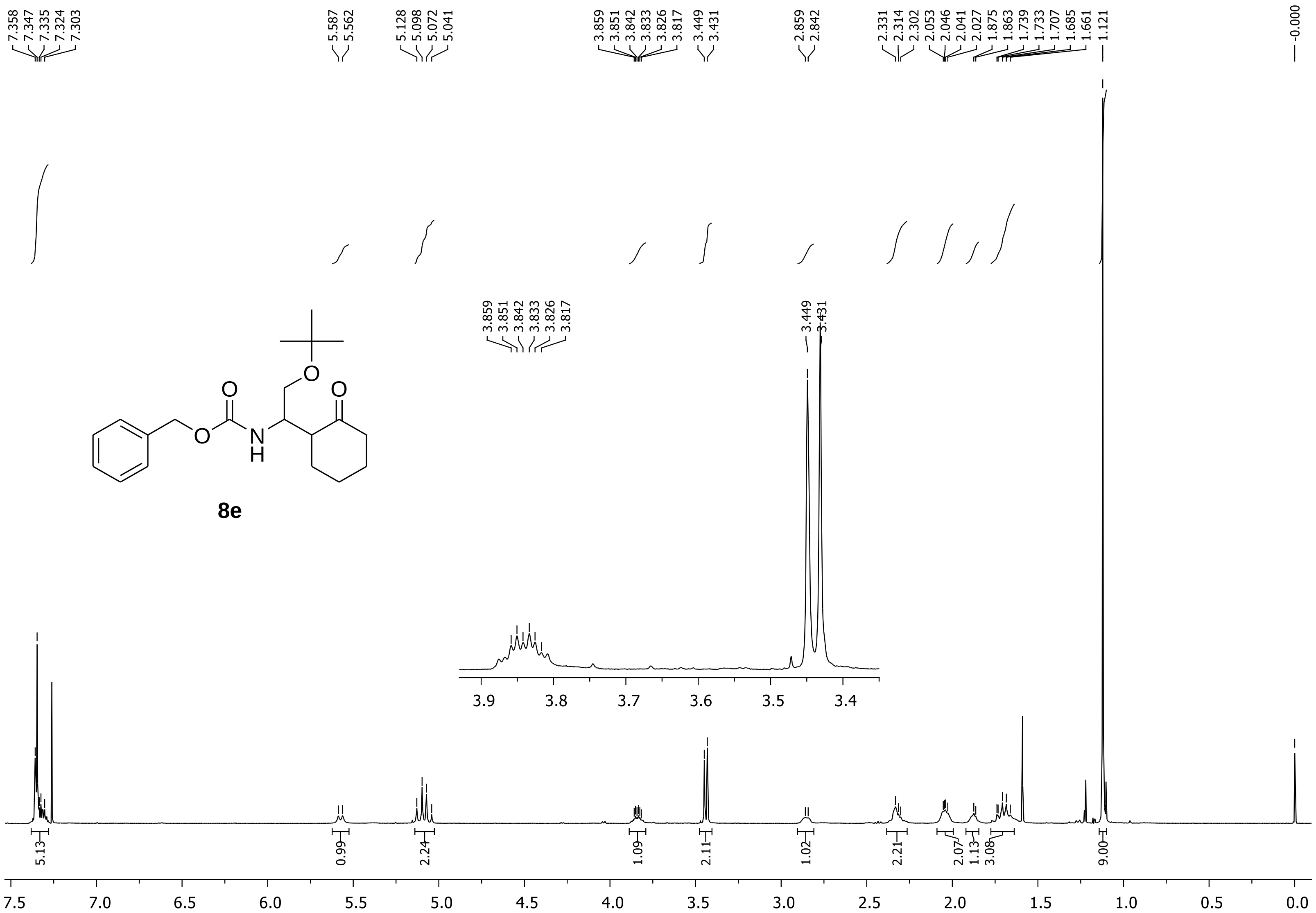

^{1}H NMR spectrum of benzyl *N*-[1-(2-oxocyclohexyl)-2-*tert*-butoxyethyl]carbamate (**8e**); 400 MHz/$CDCl_3$/TMS; δ (ppm).

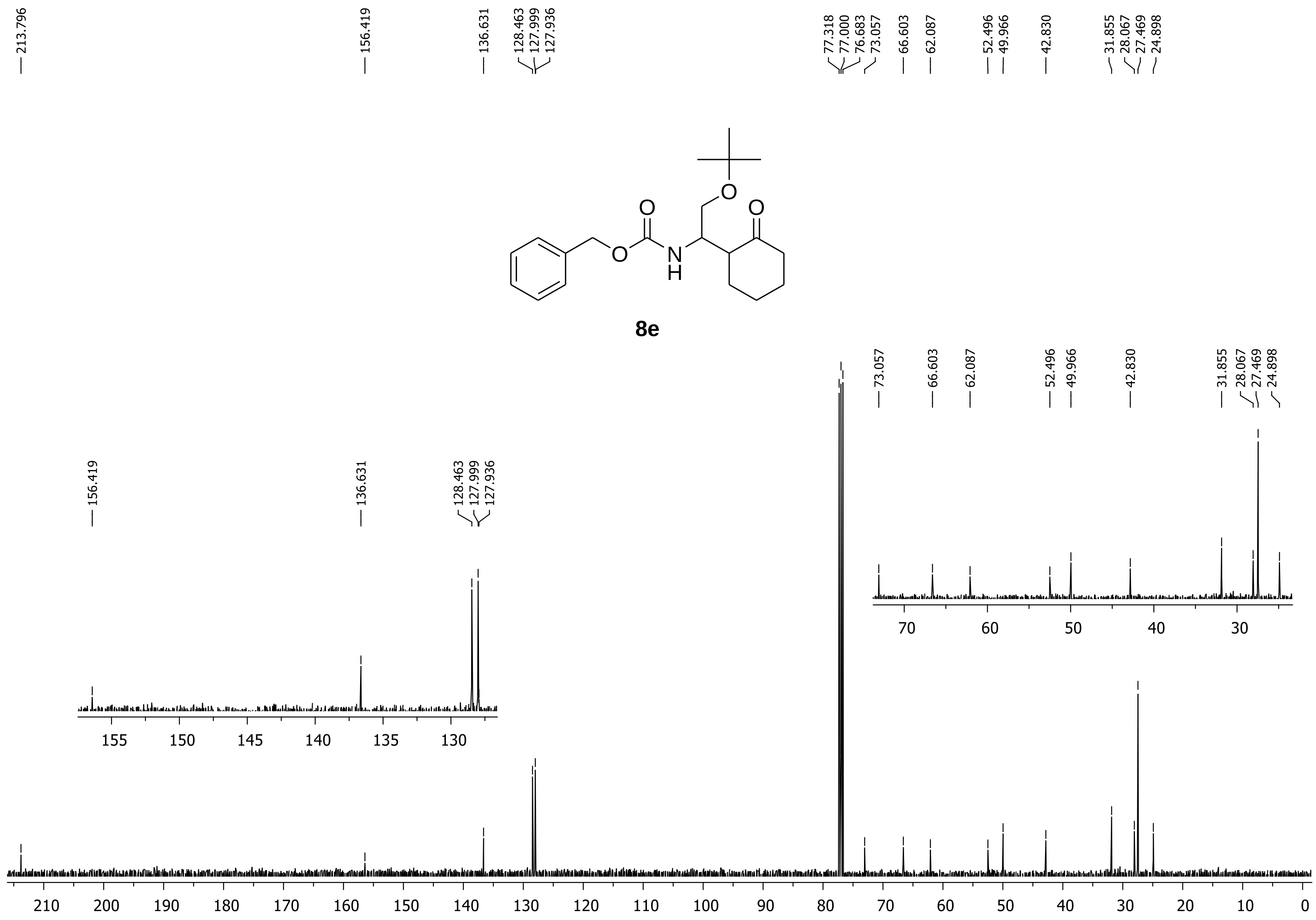

^{13}C NMR spectrum of benzyl *N*-[1-(2-oxocyclohexyl)-2-*tert*-butoxyethyl]carbamate (**8e**); 100 MHz/$CDCl_3$/TMS; δ (ppm).

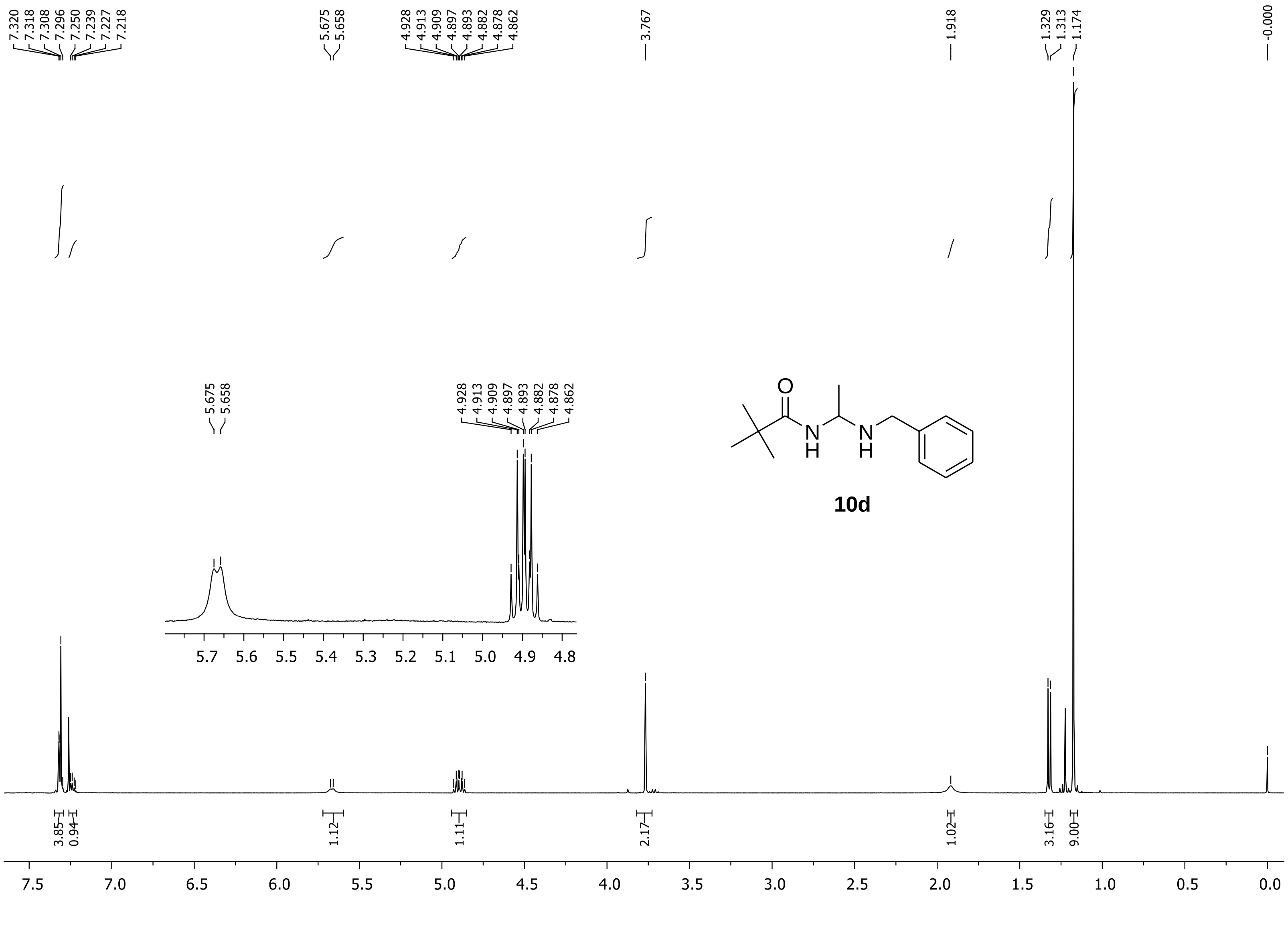

^{1}H NMR spectrum of *N*-[1-(benzylamino)ethyl]pivalamide (**10d**); 400 MHz/$CDCl_3$/TMS; δ (ppm).

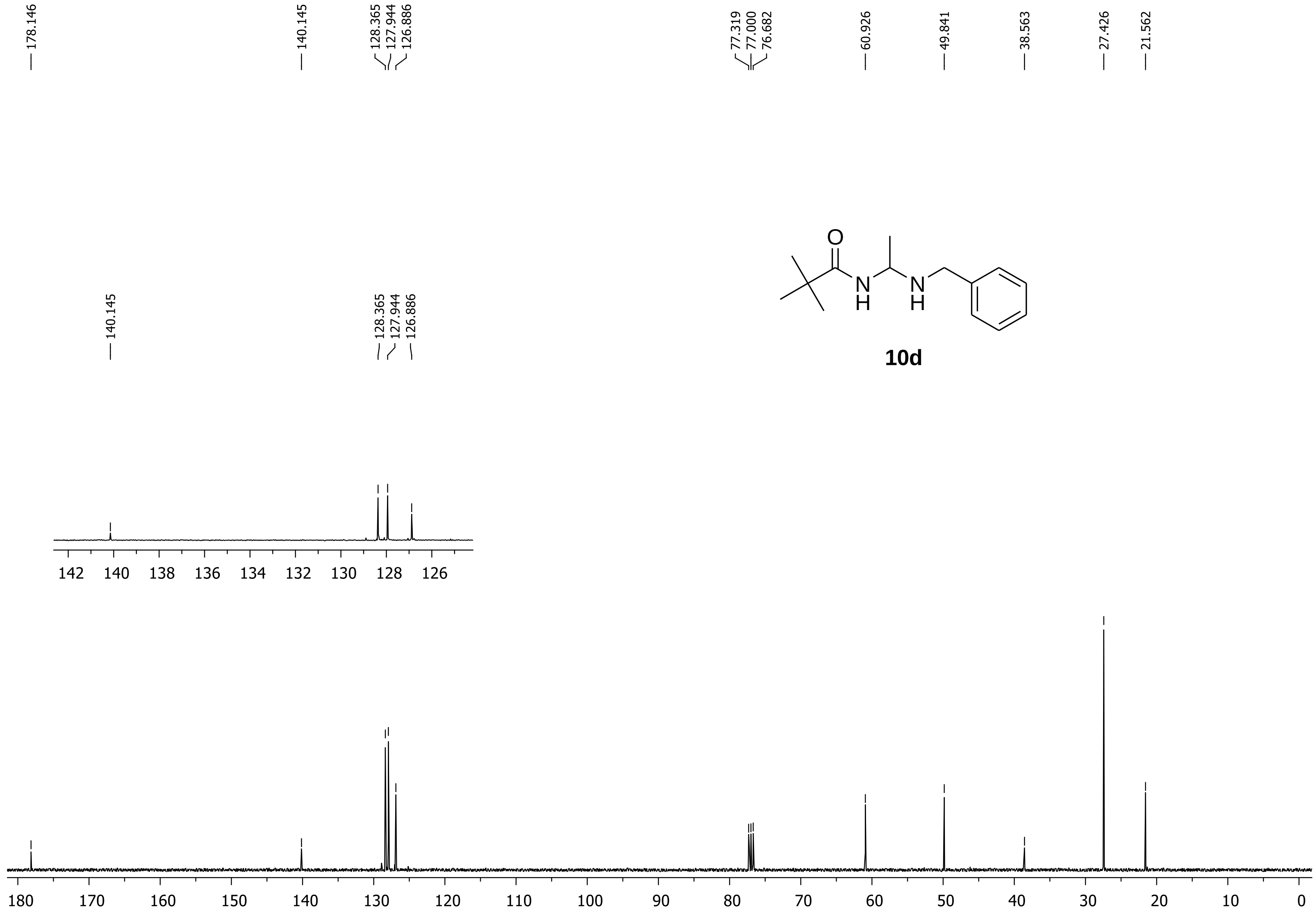

^{13}C NMR spectrum of *N*-[1-(benzylamino)ethyl]pivalamide (**10d**); 100 MHz/$CDCl_3$/TMS; δ (ppm).

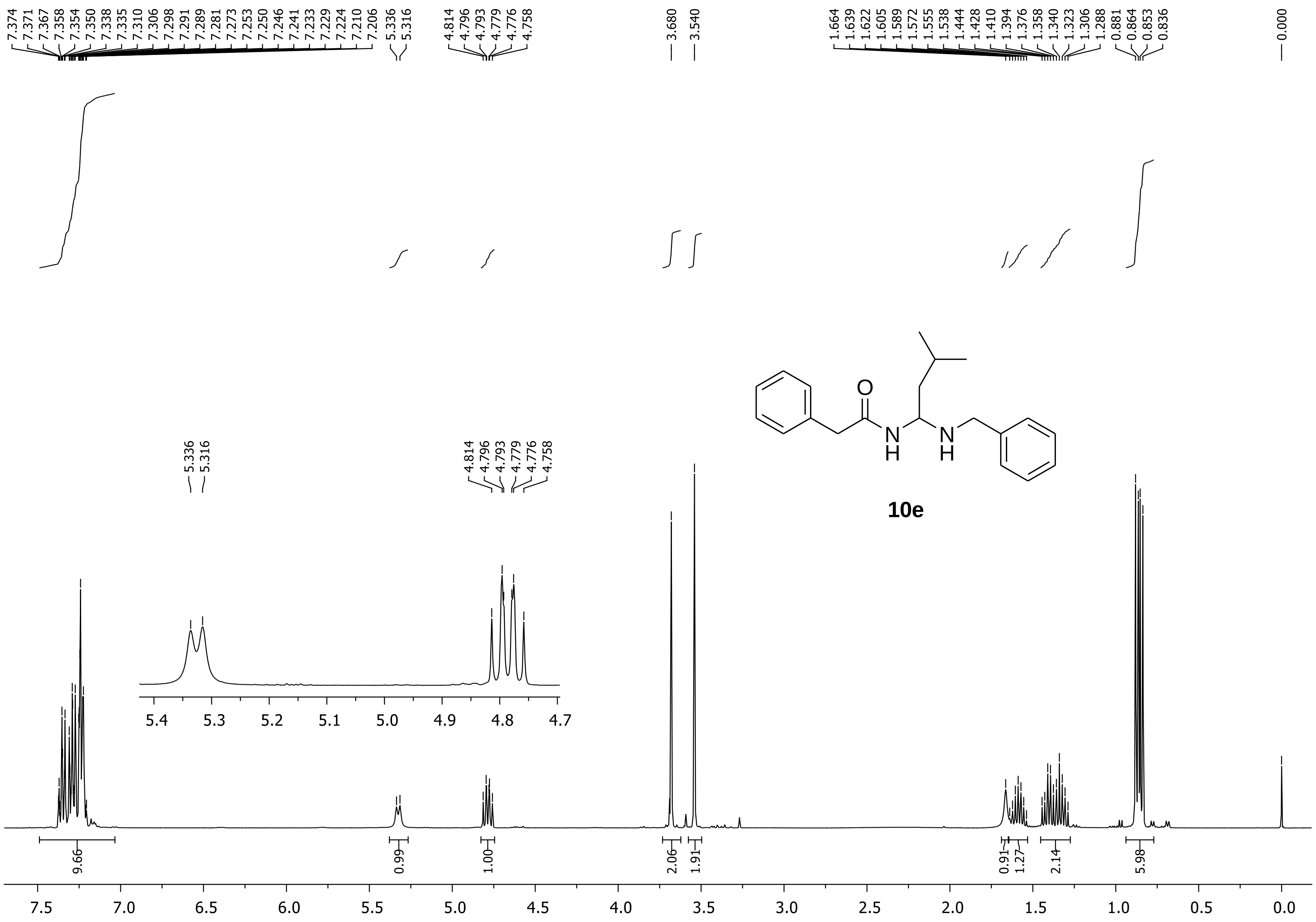

10e

^{1}H NMR spectrum of *N*-[1-(benzylamino)-3-methylbutyl]phenylacetamide (**10e**); 400 MHz/$CDCl_3$/TMS; δ (ppm).

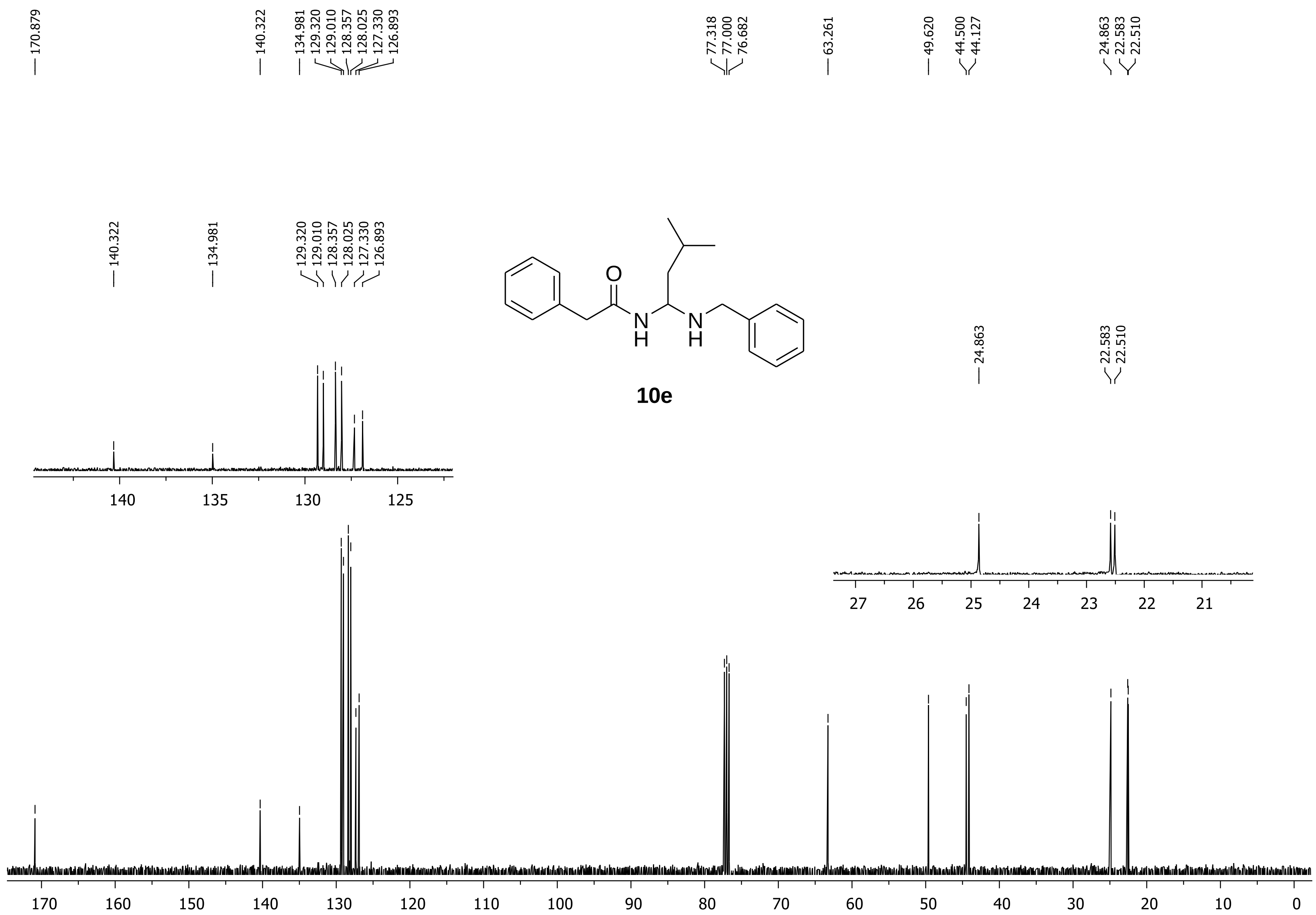

^{13}C NMR spectrum of *N*-[1-(benzylamino)-3-methylbutyl]phenylacetamide (**10e**); 100 MHz/$CDCl_3$/TMS; δ (ppm).

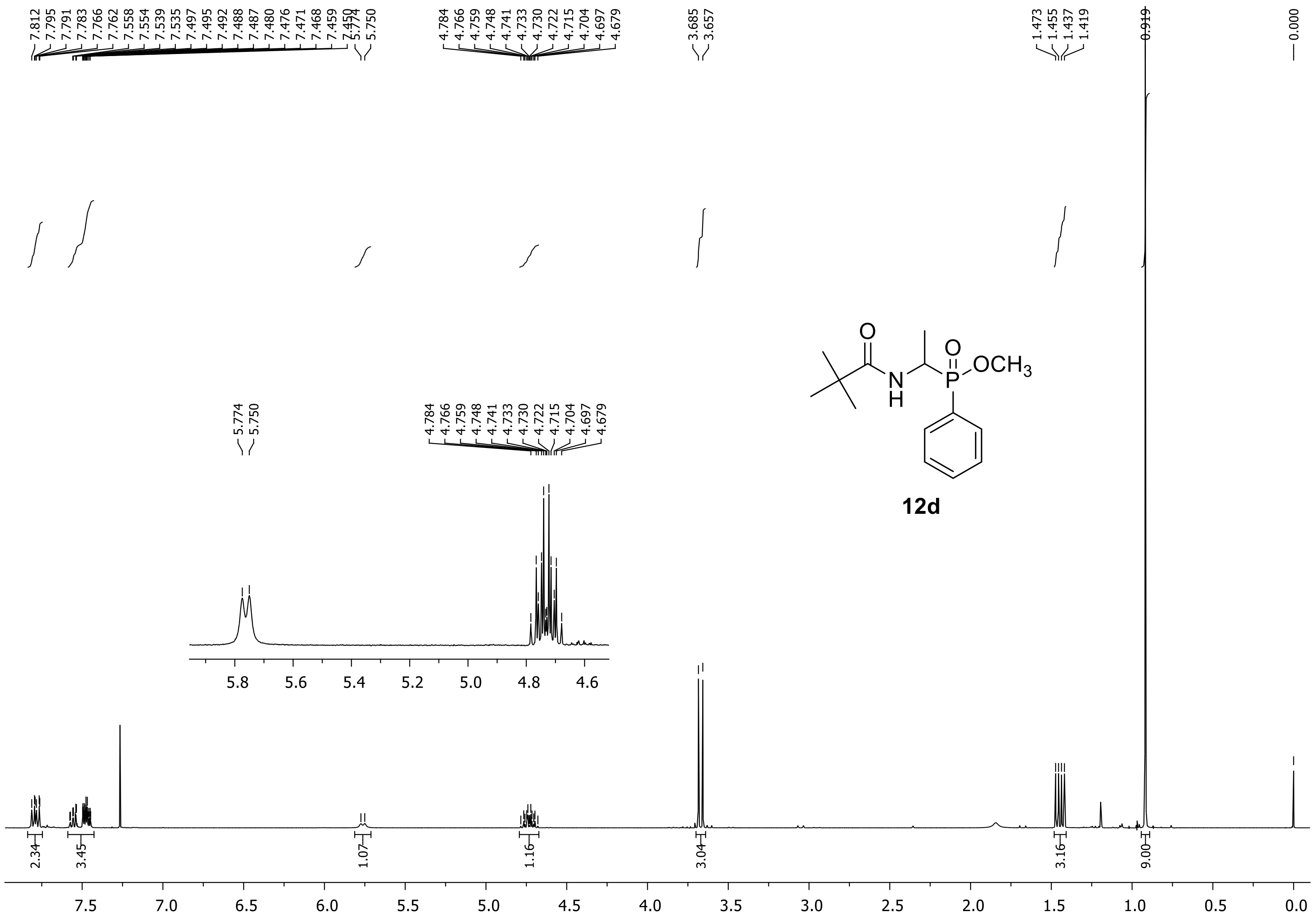

^{1}H NMR spectrum of methyl phenyl(1-pivaloylaminoethyl)phosphinate (**12d**); 400 MHz/$CDCl_3$/TMS; δ (ppm).

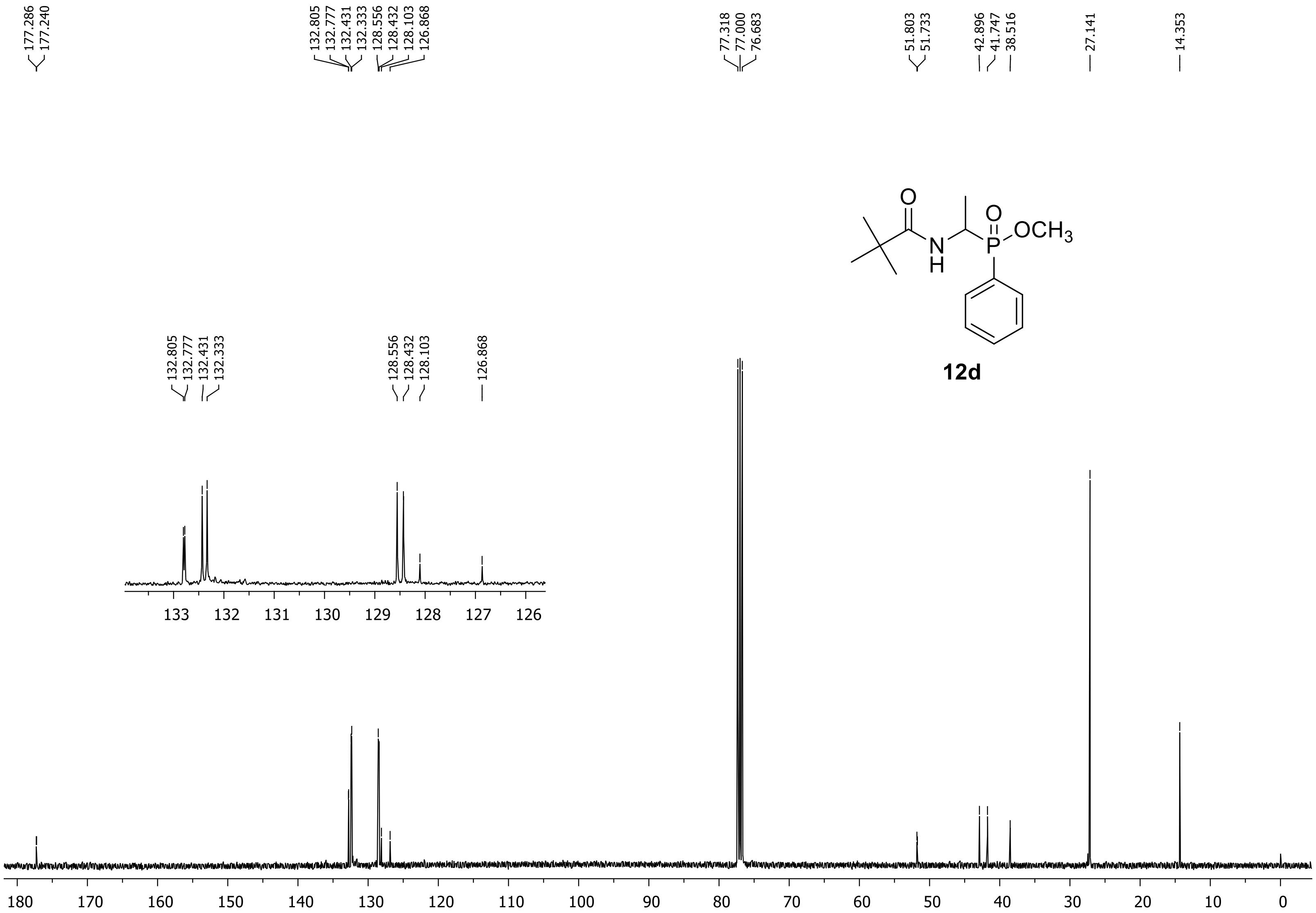

^{13}C NMR spectrum of methyl phenyl(1-pivaloylaminoethyl)phosphinate (**12d**); 100 MHz/$CDCl_3$/TMS; δ (ppm).

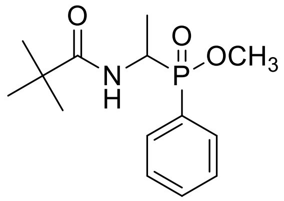

12d

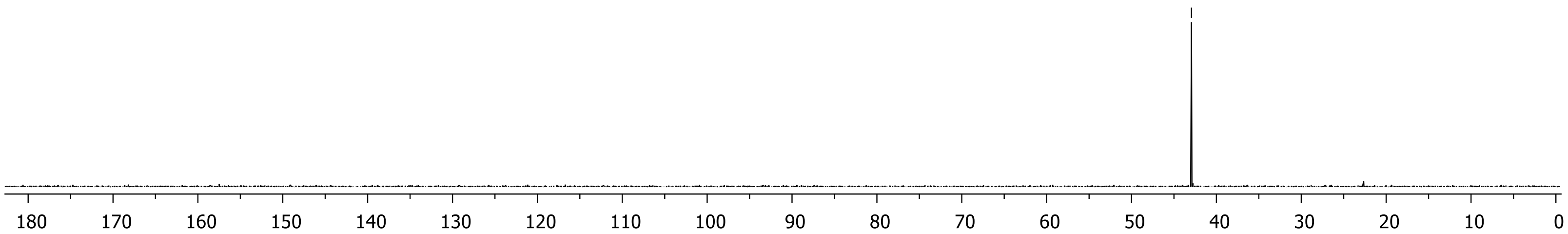

^{31}P NMR spectrum of methyl phenyl(1-pivaloylaminoethyl)phosphinate (**12d**); 161.9 MHz/$CDCl_3$; δ (ppm).

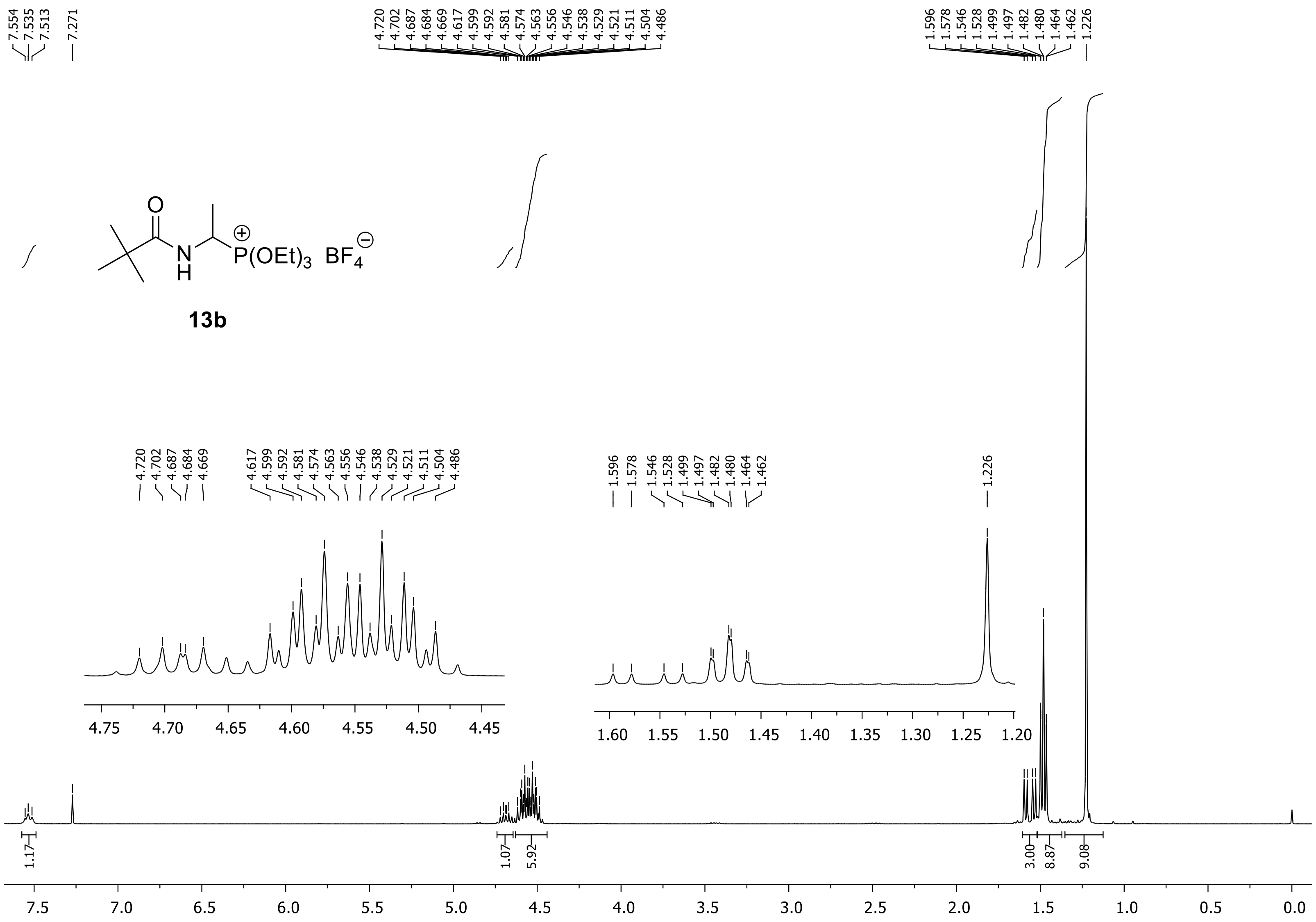

^{1}H NMR spectrum of 1-(*N*-pivaloylamino)ethyltriethoxyphosphonium tetrafluoroborate (**13b**); 400 MHz/$CDCl_3$/TMS; δ (ppm).

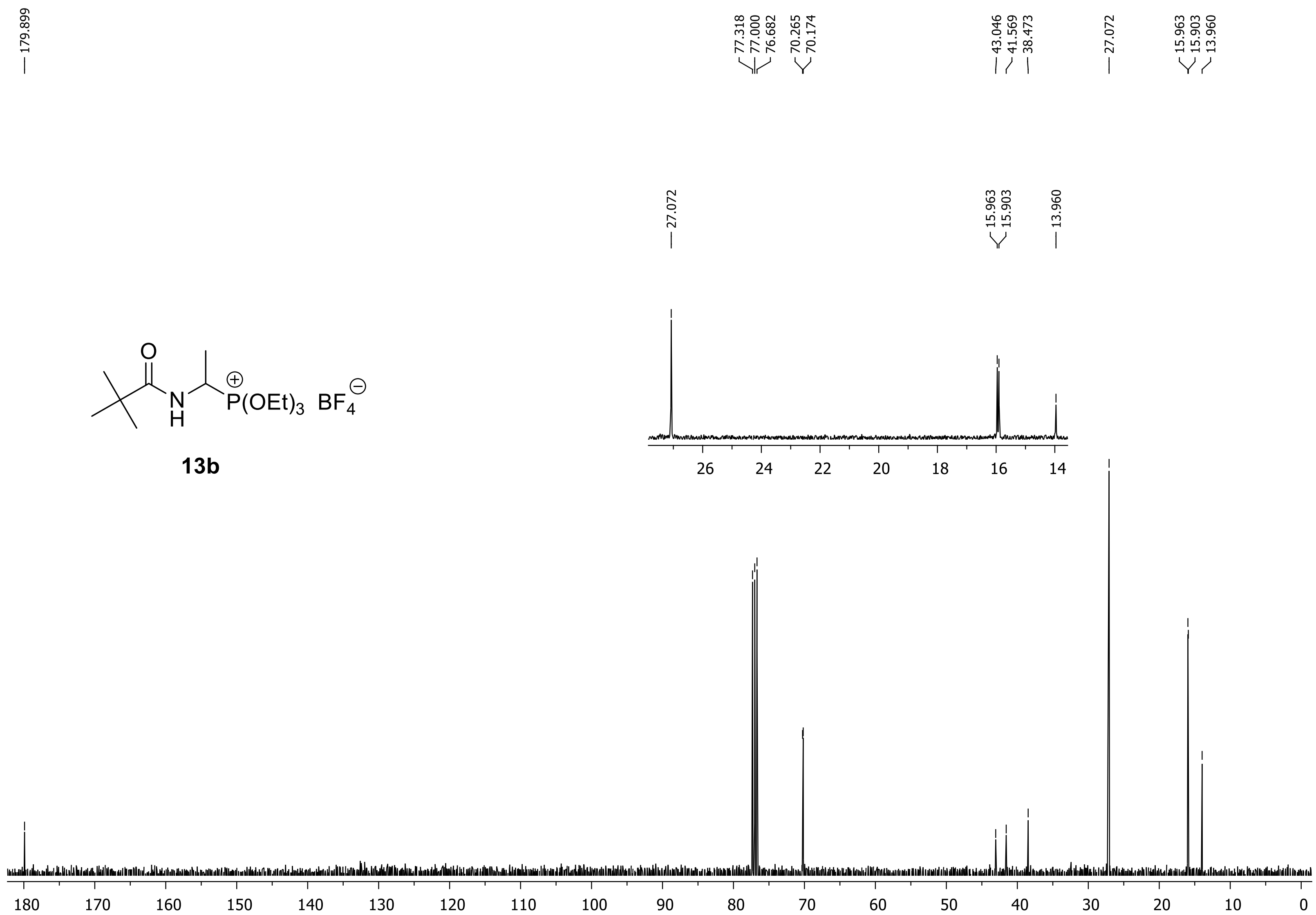

^{13}C NMR spectrum of 1-(*N*-pivaloylamino)ethyltriethoxyphosphonium tetrafluoroborate (**13b**); 100 MHz/$CDCl_3$/TMS; δ (ppm).

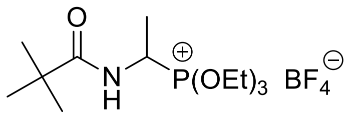

13b

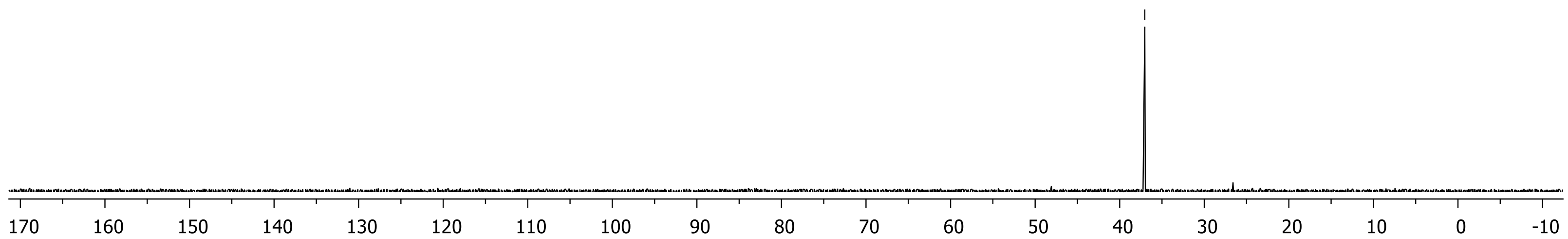

^{31}P NMR spectrum of 1-(*N*-pivaloylamino)ethyltriethoxyphosphonium tetrafluoroborate (**13b**); 161.9 MHz/$CDCl_3$; δ (ppm).

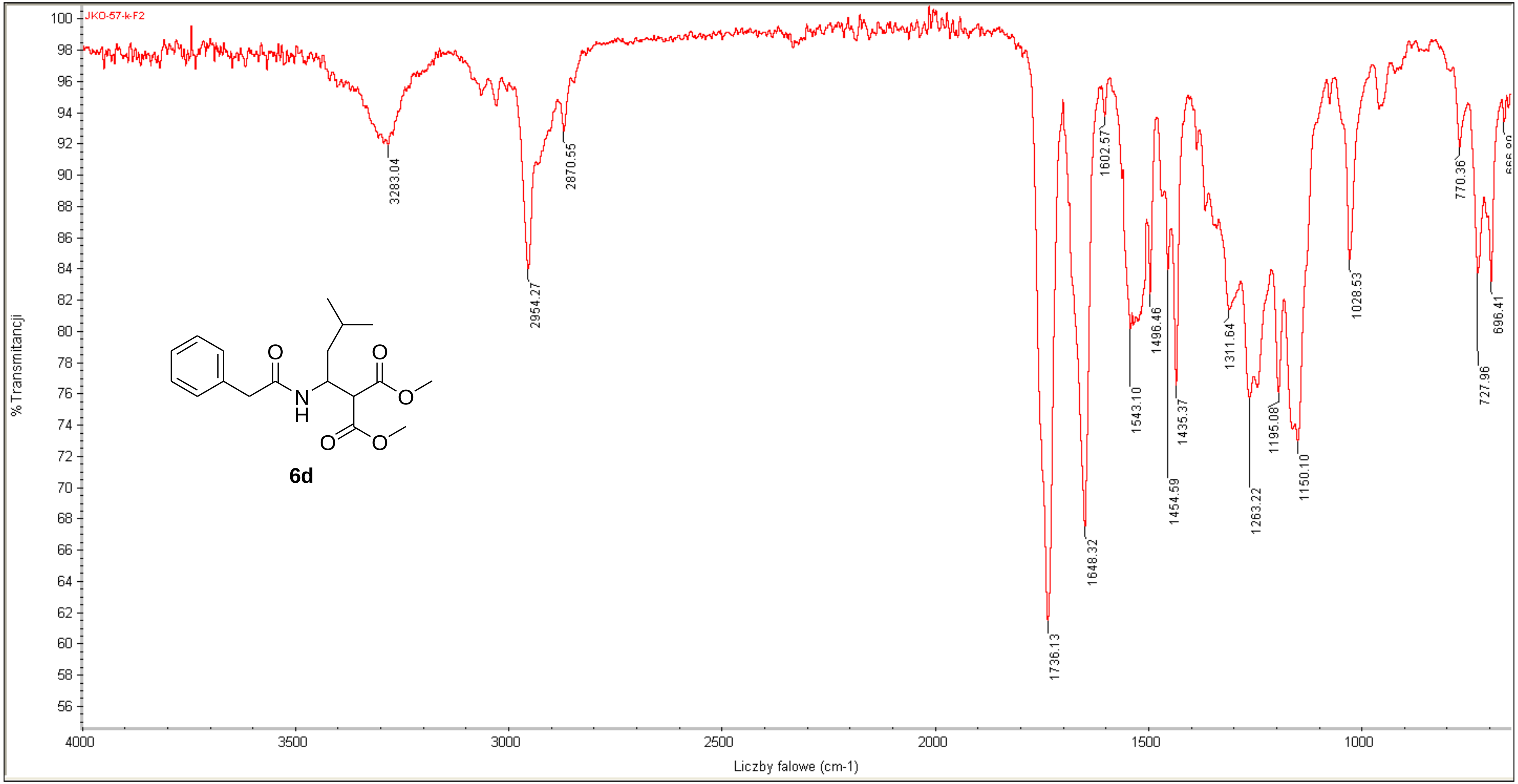

IR spectrum of dimethyl 3-methyl-1-(phenylacetylamino)butylpropanedioate (**6d**); ATR (cm^{-1}).

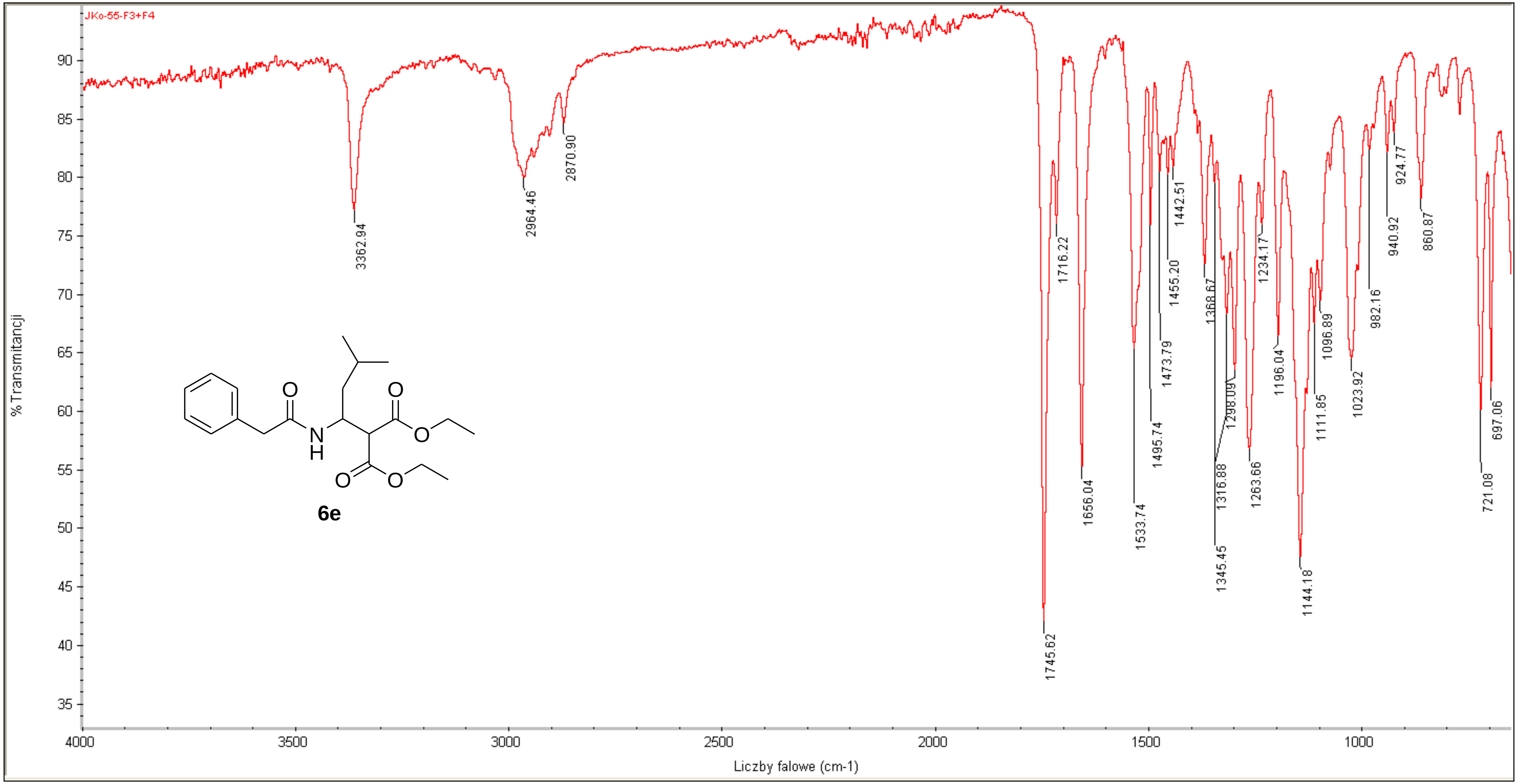

IR spectrum of diethyl 3-methyl-1-(phenylacetylamino)butylpropanedioate (**6e**); ATR (cm^{-1}).

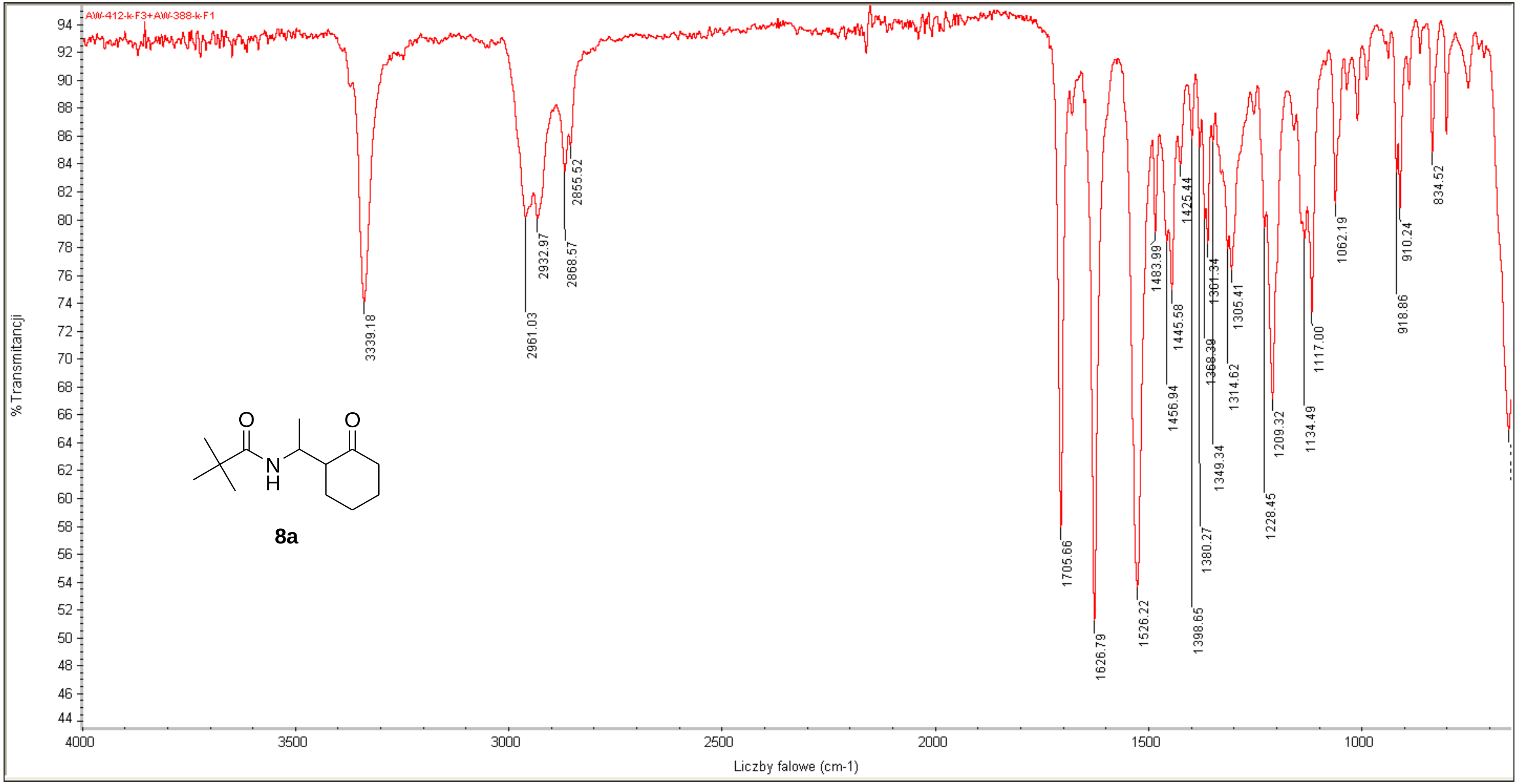

IR spectrum of *N*-[1-(2-oxocyclohexyl)ethyl]pivalamide (**8a**); ATR (cm^{-1}).

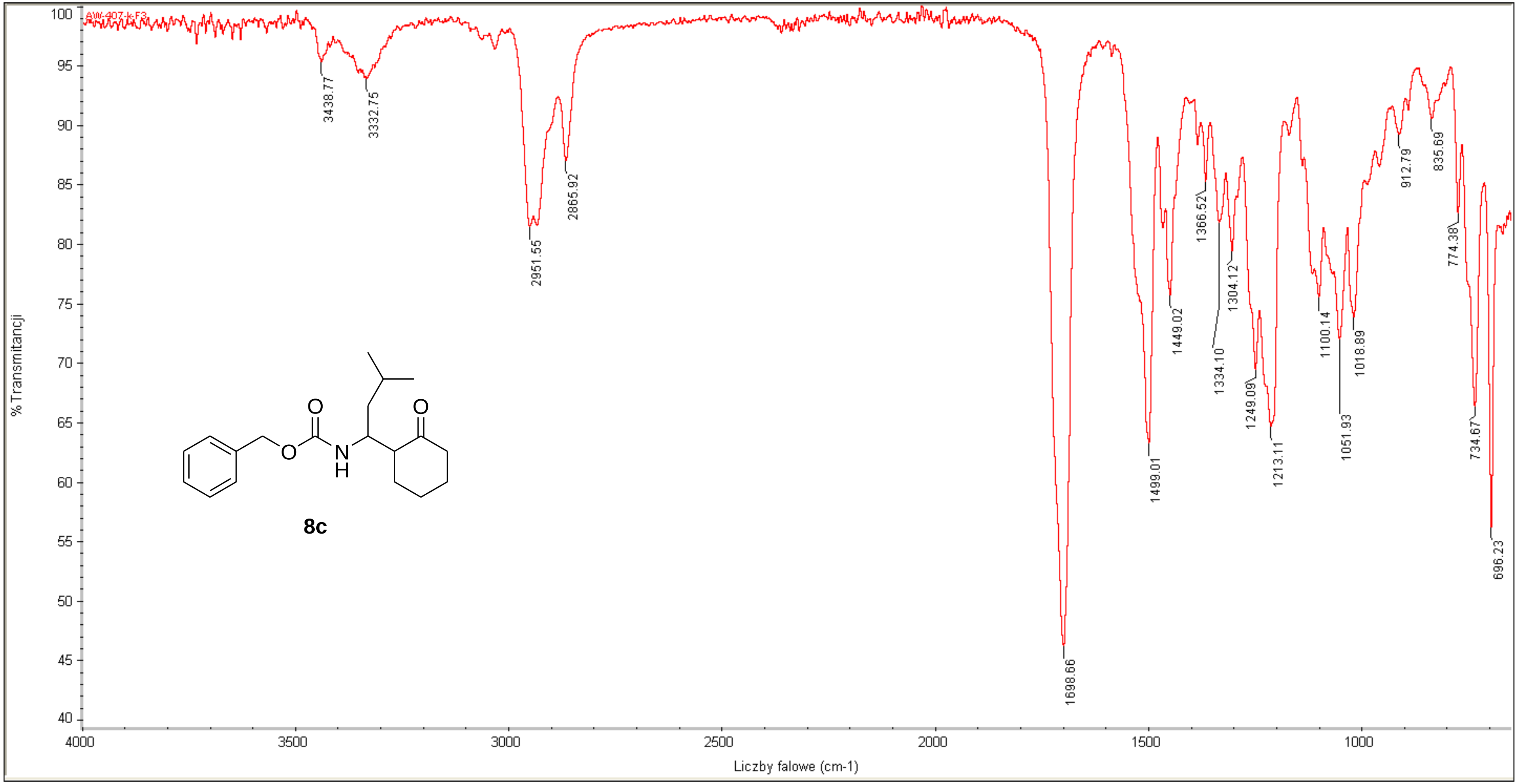

IR spectrum of benzyl *N*-[1-(2-oxocyclohexyl)-3-methylbutyl]carbamate (**8c**); ATR (cm^{-1}).

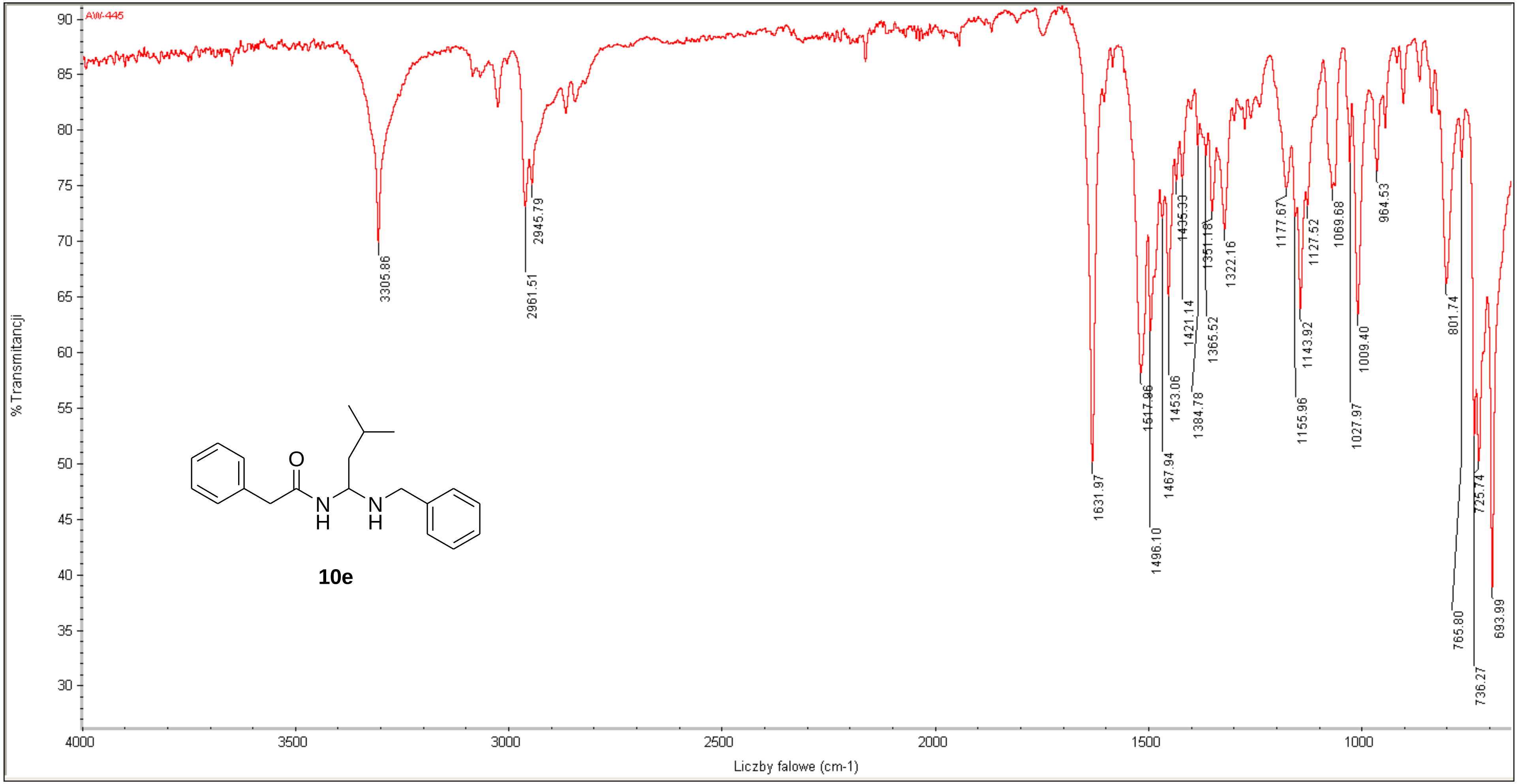

IR spectrum of *N*-[1-(benzylamino)-3-methylbutyl]phenylacetamide (**10e**); ATR (cm^{-1}).

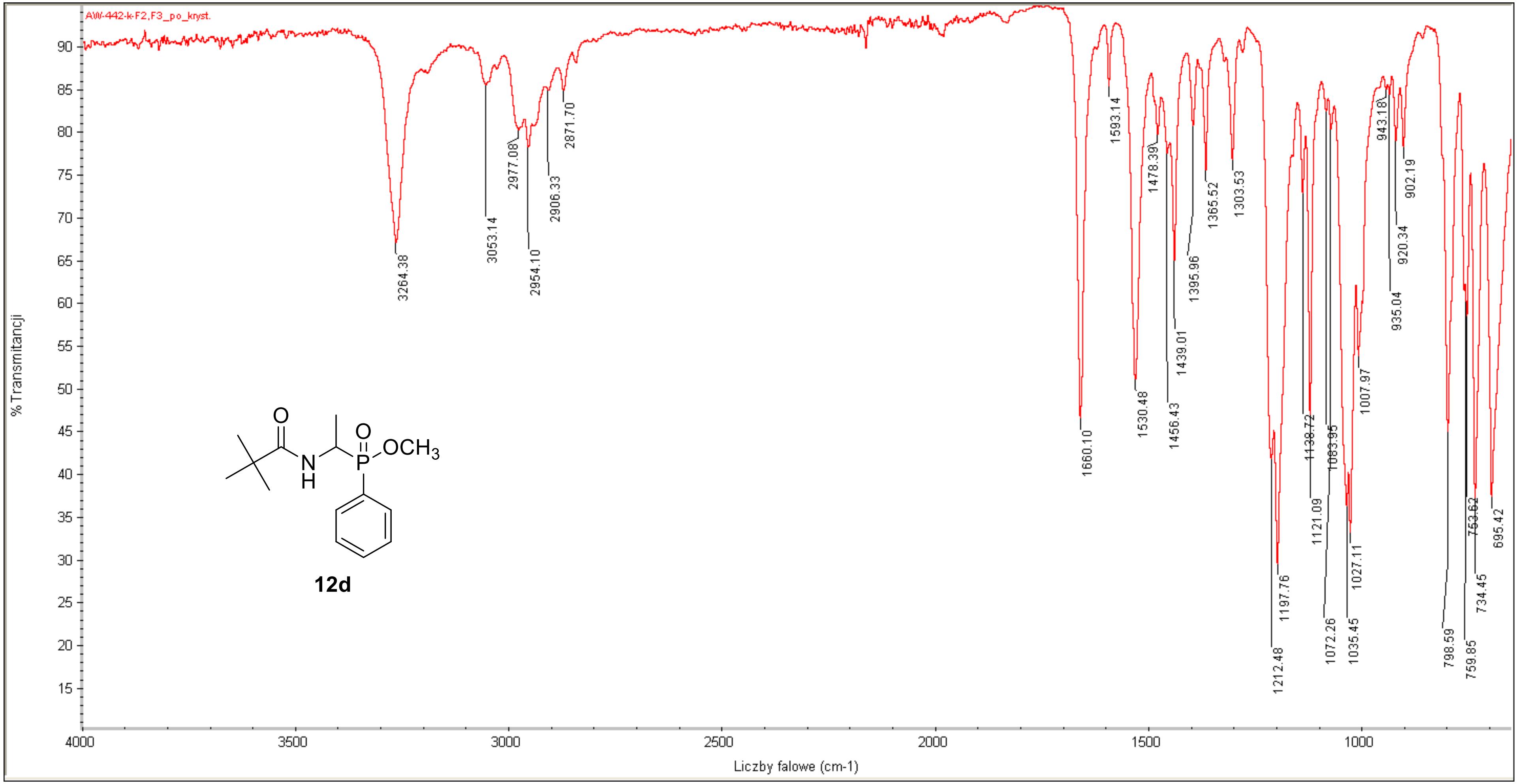

IR spectrum of methyl phenyl(1-pivaloylaminoethyl)phosphinate (**12d**); ATR (cm^{-1}).

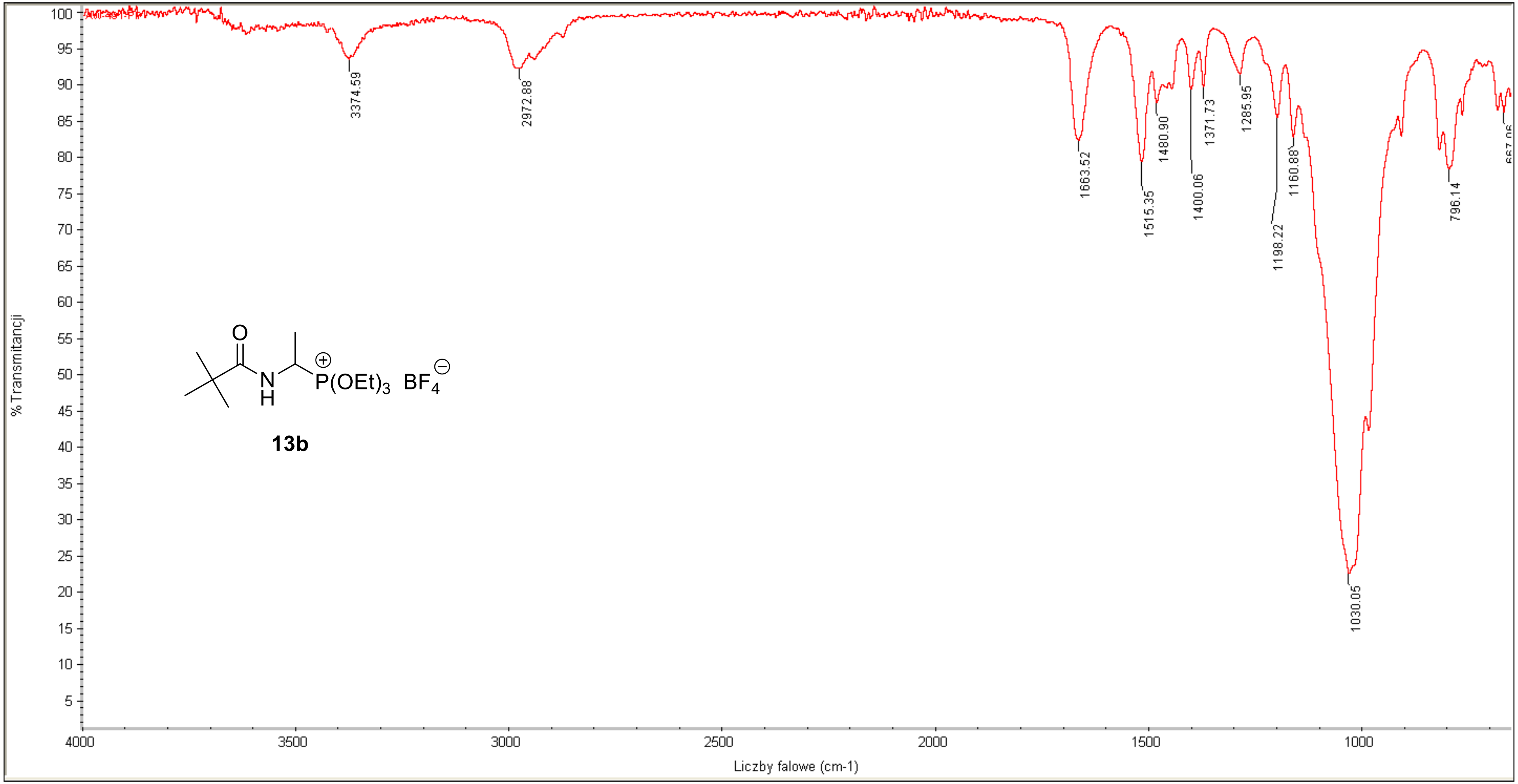

IR spectrum of 1-(*N*-pivaloylamino)ethyltriethoxyphosphonium tetrafluoroborate (**13b**); ATR (cm^{-1}).

Examples of the measurements of the changes in concentrations for the reaction of 1-(*N* pivaloylamino)ethyltris(3-chlorophenyl)phosphonium tetrafluoroborate **4c** with trimethylphosphite at 26°C

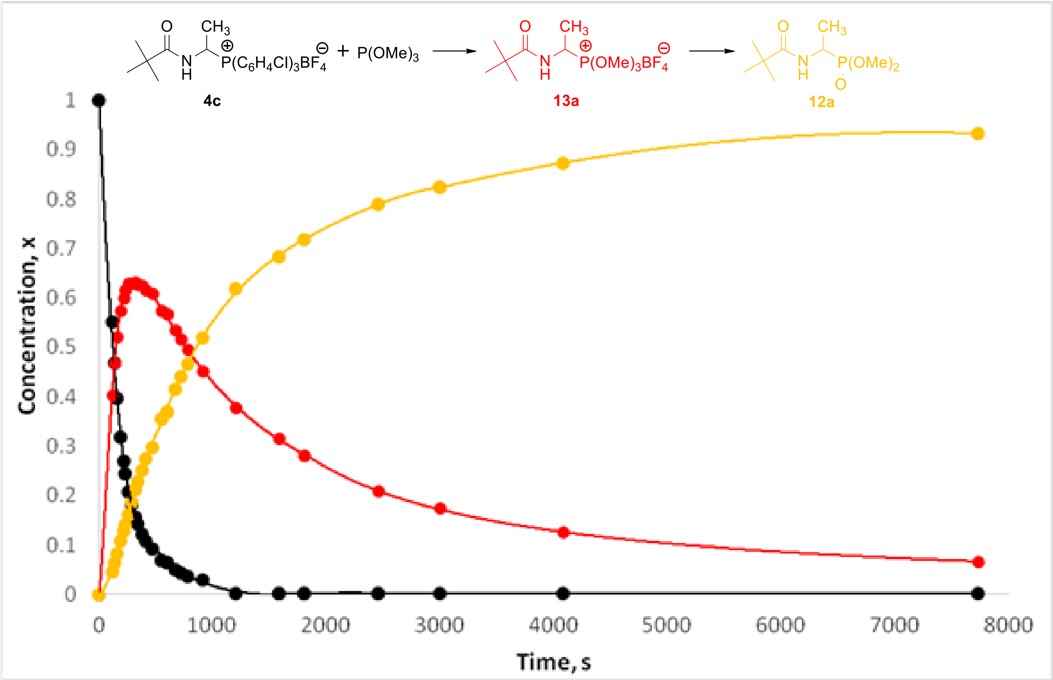

Figure 1. Concentration of the substrate **4c**, intermediate **13a** and product **12a** as a function of time for the reaction of 1-(*N*-pivaloylamino)ethyltris(3-chlorophenyl)phosphonium tetrafluoroborate **4c** with trimethylphosphite at 26°C. [1]

H NMR spectrum of 1-(*N*-pivaloylamino)ethyltris(3-chlorophenyl)phosphonium tetrafluoroborate before adding trimethylphosphite (the characteristic range: 2.0-0.0 ppm); 400 MHz/$CDCl_3$/TMS; δ (ppm).

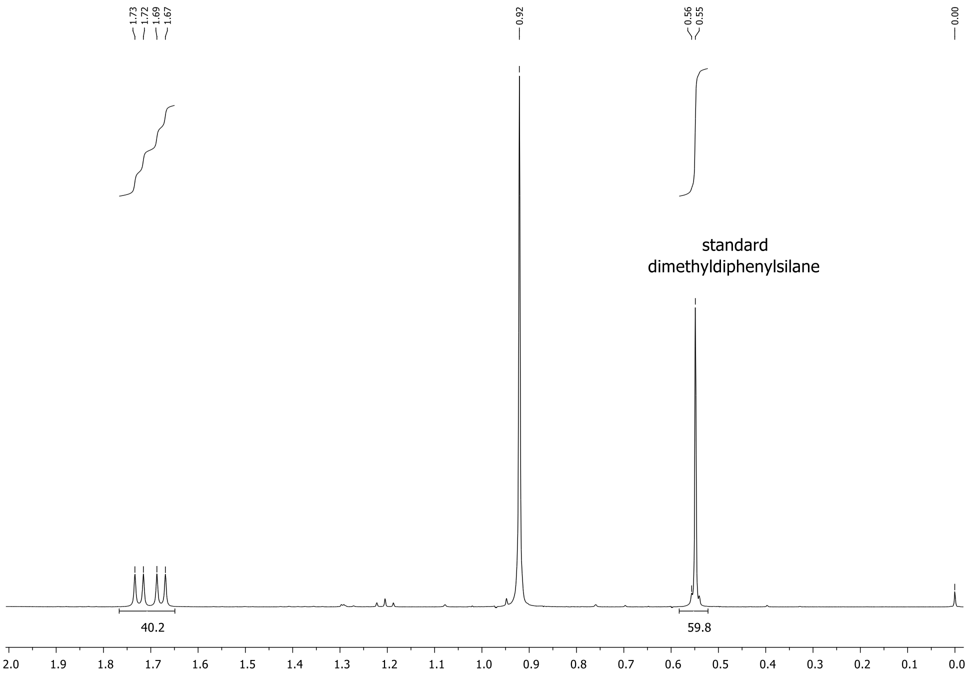

^{1}H NMR spectra of the reaction mixture after 115, 322, 668 and 7729 seconds

(the characteristic range: 2.0-0.0 ppm); 400 MHz/$CDCl_3$/TMS; δ (ppm).

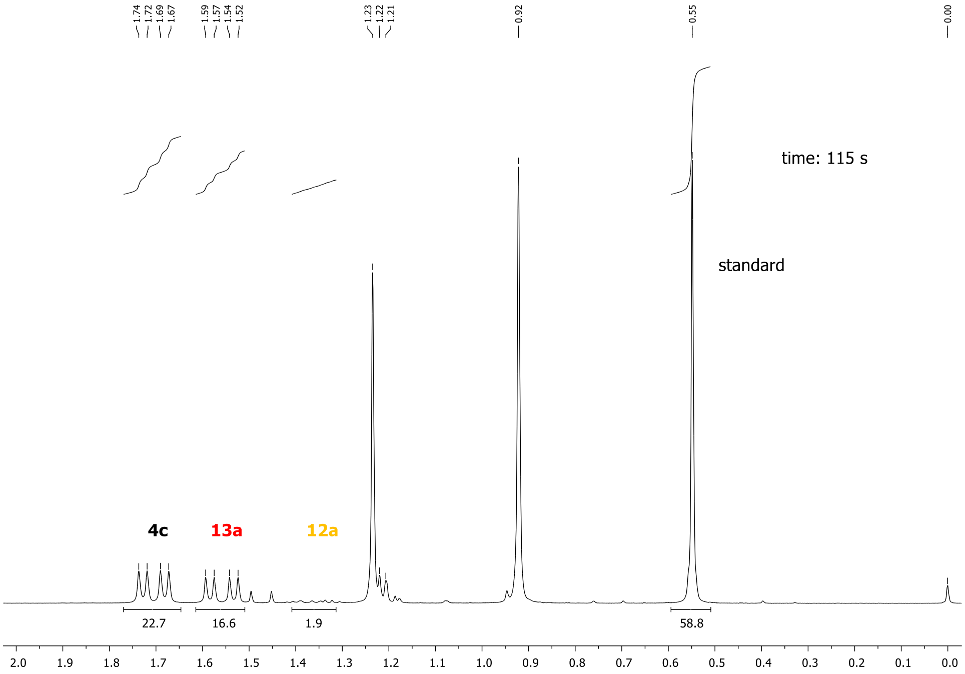

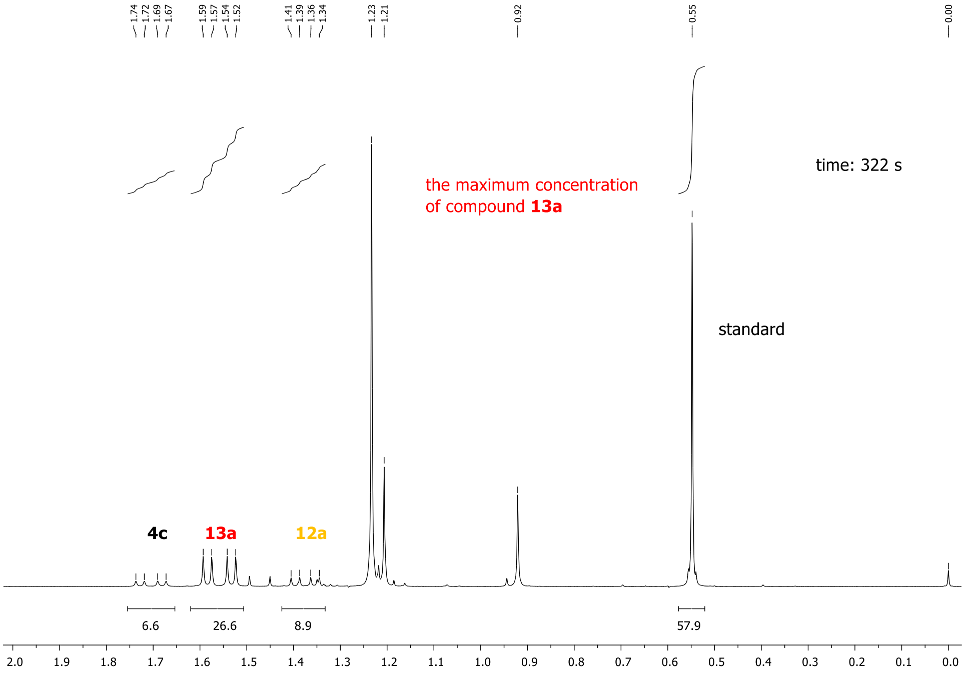

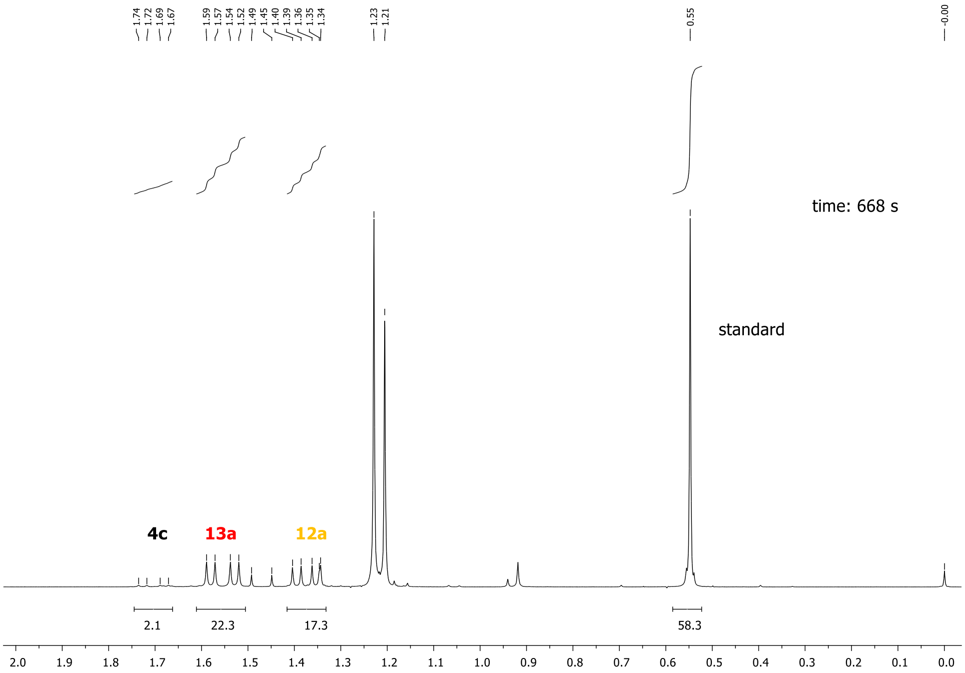
time: 668 s
standard
4c
13a
12a
2.1
22.3
17.3
58.3

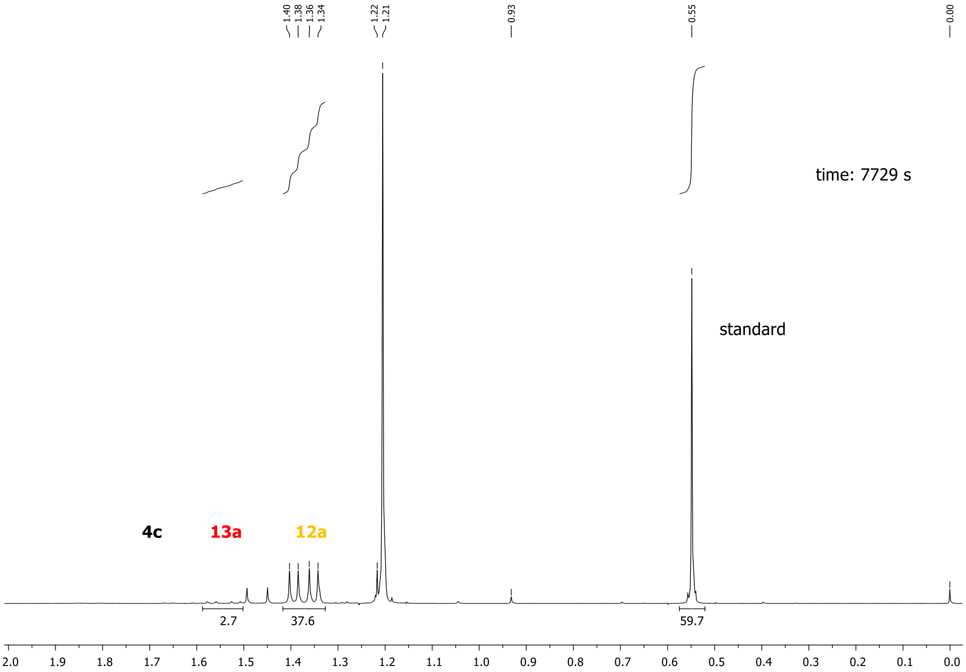
time: 7729 s
standard
4c
13a
12a
2.7
37.6
59.7

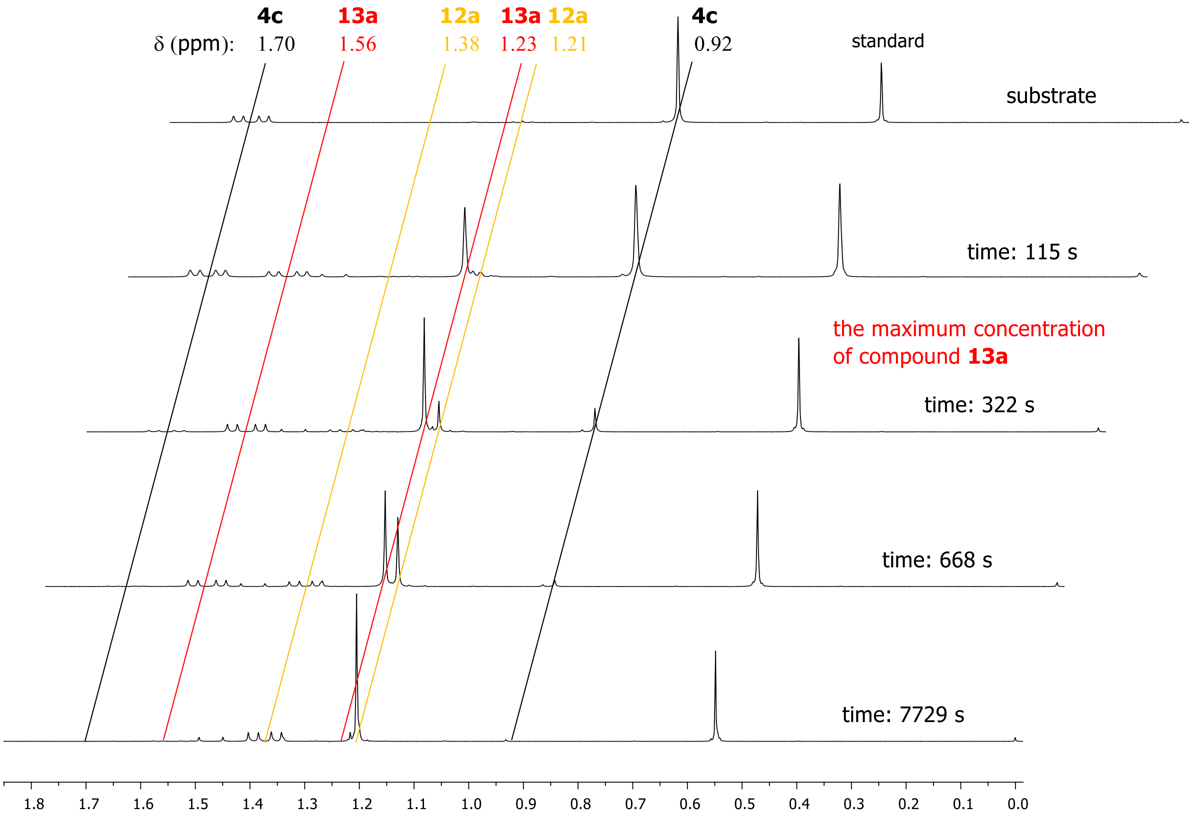
4c
13a
12a
13a
12a
4c
δ (ppm): 1.70
1.56
1.38
1.23
1.21
0.92
standard
substrate
time: 115 s
the maximum concentration of compound 13a
time: 322 s
time: 668 s
time: 7729 s
1.8 1.7 1.6 1.5 1.4 1.3 1.2 1.1 1.0 0.9 0.8 0.7 0.6 0.5 0.4 0.3 0.2 0.1 0.0
